# Largest known Mesozoic multituberculate from Eurasia and implications for multituberculate evolution and biology

**DOI:** 10.1038/srep14950

**Published:** 2015-10-22

**Authors:** Li Xu, Xingliao Zhang, Hanyong Pu, Songhai Jia, Jiming Zhang, Junchang Lü, Jin Meng

**Affiliations:** 1 Henan Geological Museum, Zhengzhou, Henan 450016, China; 2 Institute of Geology, Chinese Academy of Geological Sciences, Beijing 10037, China; 3 Division of Paleontology, American Museum of Natural History, Central Park West at 79th Street, New York, New York 10024, USA

## Abstract

A new multituberculate, *Yubaartar zhongyuanensis* gen. and sp. nov., is reported from the Upper Cretaceous of Luanchuan County, Henan Province, China. The holotype of the new taxon is a partial skeleton with nearly complete cranium and associated lower jaws with in situ dentitions. The new species is the southern-most record of a Late Cretaceous multituberculate from outside of the Mongolian Plateau in Asia and represents the largest known Mesozoic multituberculate from Eurasia. The new specimen displays some intriguing features previously unknown in multituberculates, such as the first evidence of replacement of the ultimate upper premolar and a unique paleopathological case in Mesozoic mammals in which the animal with a severely broken right tibia could heal and survive in natural condition. The phylogenetic analysis based on craniodental characters places *Yubaartar* as the immediate outgroup of Taeniolabidoidea, a group consisting of a North American clade and an Asian clade. This relationship indicates at least a faunal interchange of multituberculates before the K-Pg transition. The new evidence further supports the hypothesis that disparity in dental complexity, which relates to animal diets, increased with generic richness and disparity in body size, and that an adaptive shift towards increased herbivory across the K-Pg transitional interval.

Multituberculates are extinct mammals that lived in the Mesozoic and early Cenozoic^1^,^2^, ranging from the Middle or Late Jurassic^3^,^4^,^5^,^6^,^7^,^8^ to the Late Eocene^9^,^10^,^11^,^12^. Multituberculates were also geographically widely distributed, known from all main landmasses except for Antarctic, although identifications of some species from the southern continents remain uncertain^13^,^14^,^15^,^16^. The majority of the multituberculate species are known from isolated teeth and partial upper and lower jaws with teeth. The best-known multituberculates, represented by skulls and skeletal elements, primarily came from the Upper Cretaceous of central Asia^2^,^17^. Compared to the rich Gobi records, Mesozoic multituberculates from eastern Asia are relatively rare, being occasionally reported from Japan^18^ and China^19^,^20^,^21^.

Here we report a new genus and species of multituberculates from the Upper Cretaceous of Henan, China. This is the first record of Late Cretaceous multituberculates out of (south to) the Mongolian Plateau in Asia and the largest known Mesozoic multituberculate in Eurasia. The well-preserved specimen offers significant new evidence about the craniodental and postcranial morphologies as well as body size of multituberculates. Given that nearly all multituberculates were named on the basis of dental and/or craniodental features, our description will focus on the cranial and dental morphologies of the specimen. In addition to establishing the new taxon, we discuss replacement of the ultimate premolar as evidenced by the new specimen, a condition previously unknown in multituberculates. We also present a rare, if not the only known, case of paleopathology in Mesozoic mammals based on the healed tibia preserved in the specimen. With the new morphological data, we conduct phylogenetic analyses of a selected group of multituberculates that recover the new taxon lying basal to the clade consisting of North American and Asian taeniolabidoideans. Detailed description of the craniodental and postcranial morphology, together with more comprehensive phylogenetic analyses, will be provided in a separate study.

## Results

### Geological Setting

**Figure 1:**
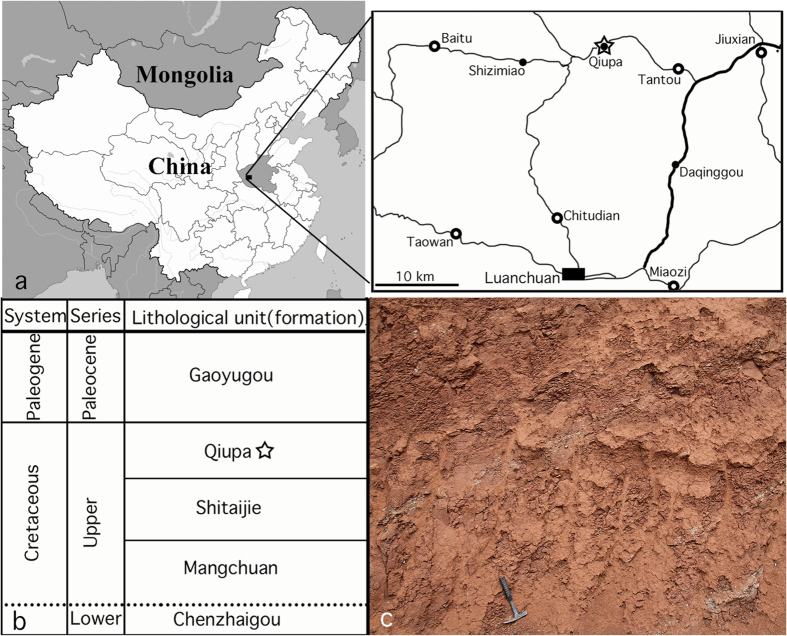
Locality and stratigraphic occurrence of *Yubaatar zhongyuanensis* gen. and sp. nov. (**a**) Locality map of the holotype in Luanchuan of Henan Province, China. (**b**) Stratigraphic position of the Upper Cretaceous Qiupa Formation. (**c**) The lithology (brownish siltstone) of the Qiupa Formation where the holotype specimen was collected. The locality map (**a**), including the close-up view, and the stratigraphic column (**b**) were created using the program of Adobe Photoshop CS6 in association with a drawing tablet by J.M. Photograph (**c**) taken by J.M.

The holotype specimen of the new multituberculate was collected from the Upper Cretaceous Qiupa Formation at Yankan village in Luanchuan County, Tantou Basin of the Henan Province in central China (Fig. 1a). The formation is dominated by brownish siltstones^22^ and is considered to be of late Late Cretaceous in age, based on stratigraphic correlation^22^,^23^ (Fig. 1b,c). The terrestrial sediments in the basin were once assigned to the “Paleocene” until several dinosaur teeth, identified as *Tyrannosaurus luanchuanensis*, were discovered^24^. Since then, numerous vertebrate fossils and dinosaur eggs have been collected from the Qiupa Formation at several exposures in the basin^23^,^25^, of which a new metatherian mammal, *Lotheridium mengi*, was recently reported^26^. The dinosaur and mammal fossils were found in beds about 30-50 m below the Cretaceous-Paleogene (K-Pg) boundary in sections where the Paleogene Gaoyugou Formation and Late Cretaceous Qiupa Formation were thought to be in conformable contact^22^. Non-mammalian taxa reported from Tantou Basin are represented by the lizard *Tianyusaurus zhengi*^27^, the dromaeosaurid *Luanchuanraptor henanensis*^28^ and the ornithomimid *Qiupalong henanensis*^23^. The Luanchuan fauna is characterized by high diversity of theropoids and was considered different from the Maastrichtian Nemegt fauna of Mongolia^25^. *Q. henanensis*, for instance, is different from any ornithomimids from the Nemegt fauna of Mongolia, supporting Luanchuan fauna as a new vertebrate assemblage^23^. Other vertebrate fossils (unpublished material) from Qiupa Formation include lizards, turtles, birds, non-avian dinosaurs (oviraptorids, troodontids, ankylosaurids, ornithopods, and ornithomimids) and dinosaur eggshells^25^. The new species reported here is the first Late Cretaceous multituberculate from outside of the Mongolian Plateau and represents the southern-most occurrence of Late Cretaceous multituberculates from Asia. The metatherian *Lotheridium* and the new multituberculate from the Luanchuan fauna of Tantou Basin probably represent the youngest known assemblage within the Mesozoic mammals of China^29^.

### Systematic Paleontology

Mammalia Linnaeus, 1758

Multituberculata Cope, 1884

Cimolodonta McKenna, 1975

*Yubaatar* gen. nov.

Type species: *Yubaatar zhongyuanensis* gen. et sp. nov., the only species.

**Figure 2:**
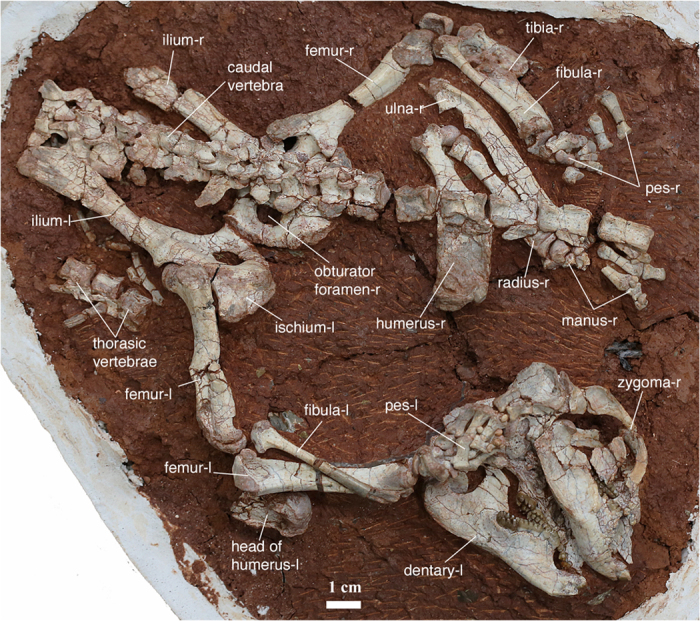
The holotype specimen of *Yubaatar zhongyuanensis* gen. and sp. nov. The holotype (41HIII0111) is housed at the Henan Geological Museum, Zhengzhou, Henan Province, China. The skull (with the lower jaws) and forelimbs were displaced, whereas the preserved posterior skeleton appears in original articulation. The suffixes -l and -r represent left and right, respectively. Photograph taken by S.J.

**Figure 3:**
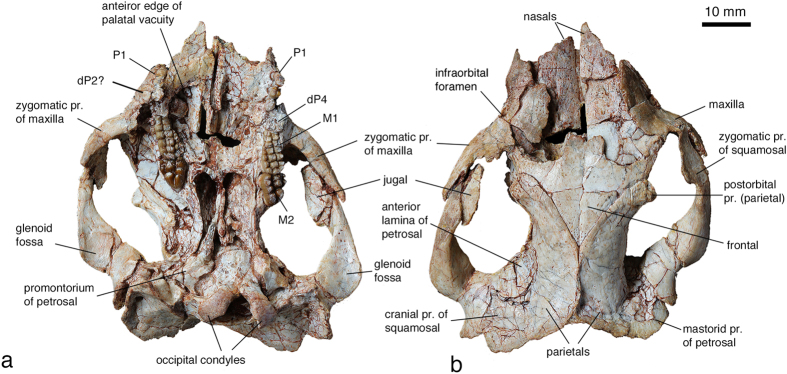
The skull of *Yubaatar zhongyuanensis* gen. and sp. nov. (**a**) Ventral view of the skull. (**b**) Dorsal view of the skull. The skull is partly compressed and distorted (see the text for description). Photographs taken by J.M.

**Figure 4:**
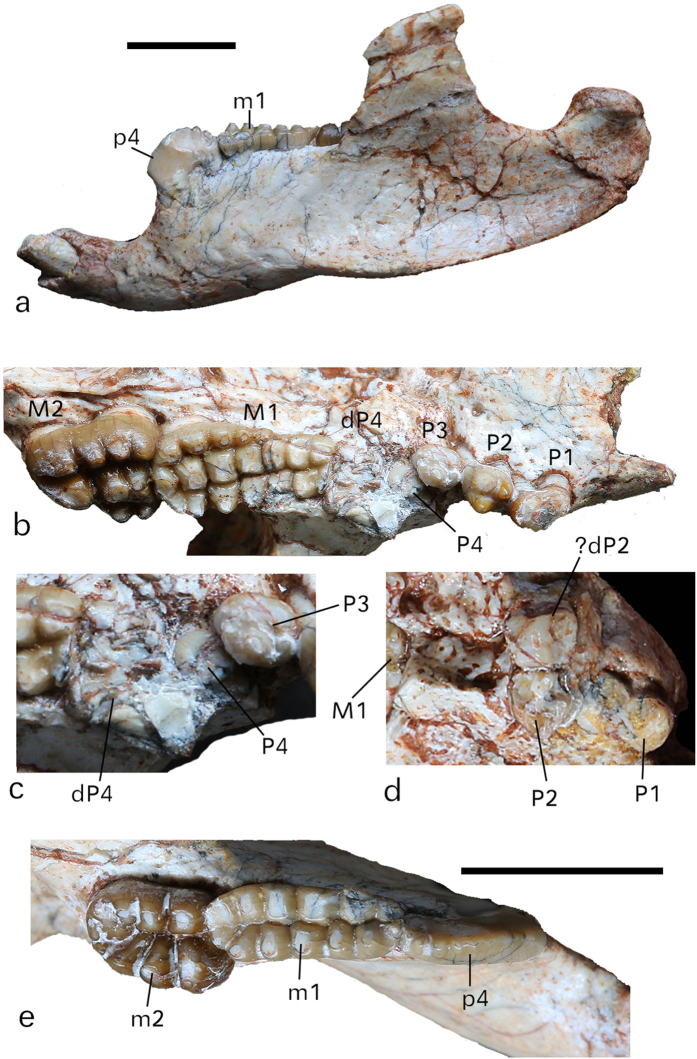
Dentition and mandible of *Yubaatar zhongyuanensis* gen. and sp. nov. (**a**) Left upper dentition in occlusal view. (**b**) The close-up view of the left dP4, P4 and P3. (**c**) The close-up view of the right P1, P2 and dP2. (**d**) Lateral view of the left lower mandible in which the incisor was worn (not broken). (**e**) The occlusal view of the left lower cheek teeth. Tooth measurements (Length/width in mm): P1: 3.25/2.16; P2: 2.95/2.27; P3: 2.35/2.16; DP4: 4.85/4.49; P4: 2.68/?; M1: 9.5/4.75; M2: 6.8/5.6; i1: 3.8/2.95; p4: 6.97/2.23; m1: 9.2/3.58; m2: 6.97/5.14. Scales are 10 mm and A and C are on the same scale. The close-up view of the dP4 is not to scale. Photographs taken by J.M.

Diagnosis: *Yubaatar zhongyuanensis* differs from other multituberculates in having the following combination of derived and primitive features: dentary length 56.6 mm and estimated skull length at least 70 mm (Figs 2 and 3); tooth formula I ? -C0-P4-M2/i1-c0-p1-m2 (see Fig. 4 caption for tooth measurements); cheek teeth lacking cusp ornamentation and coalescence; P4 small and similar to P3 in shape (provided that the identification of the molariform dP4 is correct); M1 and M2 cusp formulae 6-7:7:5 and 2:3:4, respectively, with wear facets on top of the cusps; the lingual row of M1 extending mesially more than half of the tooth length; lower incisor slender with restricted enamel primarily on the labial side of the tooth; p4 arcuate in lateral view and having eight weak serrations without or with very weak ridges on the lingual side of the tooth; p4 lacking any lingual cusp but having a small distolabial cusp labial to serration 8; the mesiolabial surface of p4 convex and ventrally extended but not forming a triangular inflated lobe (=exodaenodont lobe); m1-2 cusp formulae 6:7 and 4:4, respectively; frontals having a square-shaped anterior process that inserts between the nasals, and a posterior process projecting distally in a triangular shape between the parietals; frontal contributing anterolaterally to the medial rim of the orbital; long parietal accounting for nearly half of the skull length, with its anterior extremity forming a blunt postorbital process at the posterior rim of the small orbit; long zygomatic process of the squamosal reaching to the lateral side of the orbit; jugal abutting the medial side of the maxilla-squamosal junction on the zygomatic arch; pterygopalatine ridge of the pterygoid joining the anterior ridge of the promontorium that has an irregular and uneven surface; petrosal forming a substantial part of the nuchal crest; vestibule not inflated.

Etymology: “*Yu*”, pinyin spelling of the Chinese character that is the short form for Henan Province; “-*baatar*”, hero in Mongolian, a common suffix for generic names of many Asian Cretaceous multituberculates.

*Yubaatar zhongyuanensis* sp. nov.

**Figure 5:**
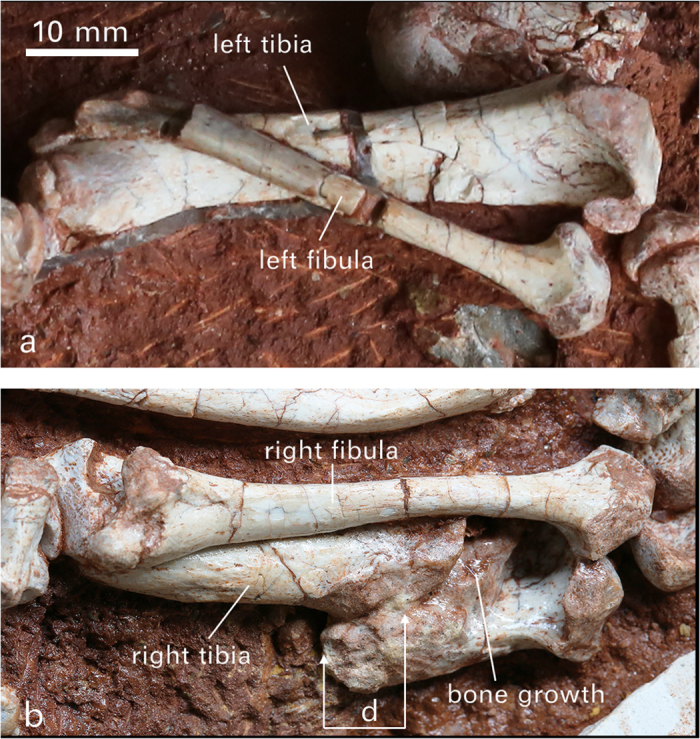
Comparison of normal and broken tibiae of *Yubaatar zhongyuanensis* gen. and sp. nov. (**a**) The normal (left) tibia and fibula. (**b**) The normal (right) fibula and the abnormal (broken but healed) tibia. The diaphysis of the right tibia was broken and displaced but fully healed (**b**). The breakage is at about two fifth of the midshaft closer to the proximal end of the bone and has a simple and oblique fracture plane. The length of the intact left tibia measures 56.4 mm, whereas the broken right one is 49.6 mm. Thus, the breakage resulted in a 6.8 mm shortening of the diaphysis (as shown by “d” in **b**), about 12% of the length of the tibia. As preserved, the proximal end is distally shifted, resulting in a gap between the proximal end of the tibia and the distal end of the femur. The two fractured portions of the tibia are not positioned in parallel, which must have increased the difficulty of the healing process. The broken surface on the proximal portion of the tibia is still visible and the areas of the two segments were surrounded with rugged ossified callus. In addition to the rugged ossification that juxtaposed the two segments of the tibia, there is a new bone outgrowth on the proximal segment that is fused to the proximal fracture surface of the distal segment of the tibia. Photographs taken by J.M.

Holotype: A partial skeleton with nearly complete cranium, associated dentary bones and partial postcranium (catalog number: 41HIII0111; field number: L08-61; Figs 2 and 5). The specimen is housed in the Henan Geological Museum, Zhengzhou, Henan Province, China.

Diagnosis: Same as for the genus.

Type locality and age: Qiupa Formation at Yankan village in Luanchuan County, Tantou Basin, Henan Province, China; late Late Cretaceous^22^,^23^.

Etymology: The species name is after Zhongyuan, ancient name of the geographic area of today’s Henan Province.

### Description

The holotype of *Yubaatar zhongyuanensis* shows the diagnostic craniodental features of multituberculates, such as cheek teeth with multiple cusp rows, molar cusps subequal in height and arranged in at least two longitudinal rows, M2 positioned one cusp row lingual relative to M1, a single pair of enlarged lower incisors, ultimate lower premolar with serrated dorsal margin and oblique fine ridges on its lingual surfaces, low and wide skull with the jugal on the medial side of the zygomatic arch (Figs 3 and 4). *Yubaatar zhongyuanensis* differs from tritylodontids^30^ in having only two upper and lower molars that have pyramidal cusps and in lacking the postdentary trough, thus the postdentary bones, on the medial side of the dentary. It differs from non-allotherian mammaliaforms in having only two upper and lower molars with at least two rows of multiple cusps. It differs from “haramiyidan” allotherians^31^,^32^,^33^,^34^,^35^,^36^ in having molar cusps of nearly equal heights and M2 displaced one cusp row lingual relative to M1.

The Late Cretaceous *Yubaatar* is unquestionably a member of Cimolodonta that contain some Early Cretaceous (Aptian-Albian) and all Late Cretaceous and Paleogene multituberculates^1^,^2^,^6^. *Yubaatar* possesses the common cimolodont dental formula: 2.0.1-4.2/1.0.1-2.2. Its p4 is arcuate (plesiomorphic) with weak oblique enamel ridges (shared with Arginbaataridae), rather than being rectangular as in ‘Plagiaulacida’. Among cimolodontans *Yubaatar* is similar to several advanced taxa in having only one lower premolar, such as some microcosmodontids (*Acheronodon, Pentacosmodon*), eucosmodontids (*Stygimys, Eucosmodon*), taeniolabidoideans (*Catopsalis*, *Taeniolabis, Lambdopsalis*, *Sphenopsalis* and *Prionesus*), *Buginbaatar* and Kogaionidae (*Barbatodon*)^1^,^2^,^6^,^37^,^38^.

Cranial morphology **—** The rostrum of the skull is crashed and the premaxilla and upper incisors are not preserved, but it is still discernible that the rostrum was broad and probably short in relation to the mandible (Figs 2 and 3). In ventral view (Fig. 3a), the palatal process of the maxilla is broken, but the preserved portion of the palate shows that a pair of large palatal vacuities is present. The alisphenoid forms the lateral wall of the lateral pterygopalatine trough within the choana that has the anterior border levels with the M1-M2 junction. Posteriorly, the alisphenoid extends to the ear region, but the suture delimiting its posterior end is unclear. The zygomatic process of the squamosal anterior to the glenoid fossa is strong and subequal in length to the zygomatic process of the maxilla. The glenoid fossa is slightly concave. The exoccipital and basioccipital appear fused. The axial portion of the basioccipital is rectangular and slightly convex. The occipital condyles have well defined and convex articular facets and are separated by a narrow odontoid notch. In the ear region, the most conspicuous feature is the promontorium of the petrosal. The surface of the promontorium is irregular and bulged ventrally into a blunt ridge that extends anteromedially. This bony ridge may serve as the supporting site for the medial sides of the malleus and ectotympanic. There is no sign of any groove for either the internal carotid or stapedial artery on the ventral surface of the promontorium. Two apertures, the fenestra vestibuli and the perilymphatic foramen, separated by a narrow crista interfenestralis, characterize the posterior end of the promontorium.

In dorsal view (Fig. 3b), the nasal is large and forms most of the roof of the nasal cavity; it has an extensive contact with the maxilla laterally and the frontal posteriorly, and bears 4-5 vascular foramina. The lacrimal occupies the anteromedial corner of the orbit and has a narrow exposure on the skull roof. The anterior portion of the frontal is broad, with its lateral wing forming the dorsomedial rim of the orbit. Anteriorly, the frontals form a bilobed square-shaped process that inserts between the nasals. The frontal narrows posteriorly into a triangular process that wedges between the parietals. The facial and zygomatic processes of the maxilla are large. The facial process occupies the preorbital region lateral to the nasal and contributes to the anterior rim of the orbit. The single infraorbital foramen opens on the rostrum within the maxilla at the level of P4. The zygomatic process of the maxilla is strong; it tapers posteriorly and is overlapped by the long zygomatic process of the squamosal. On the medial side of the conjunction between the maxilla and squamosal there is the plate-like jugal. The parietal is extensive and roofs most of the cranial cavity. Anteriorly, the parietals contact the frontals in a broad V-shaped suture. The anterior portion of the parietal extends to the orbit and forms a blunt postorbital process that has a rough surface and defines the posterior rim of the small orbit. Posteriorly, the parietals form the medial portion of the nuchal crest. In lateral view, the most prominent feature of the petrosal is a large anterior lamina that contacts the parietal dorsally, the frontal, orbitosphenoid, and the alisphenoid anteriorly, and the squamosal posteriorly. The dorsal flange of the squamosal is extensive and sutures with the anterior lamina of the petrosal anteromedially, the parietal medially, and the dorsal exposure of the petrosal posteriorly.

Mandible **—** The mandible is 56.6 mm long, measured from the mesial edge of the incisor alveolus to the distal end of the mandibular condyle (Fig. 4a). The depth of the mandible is 13.1 mm on the labial side at the mesial part of m1. The horizontal ramus is robust and the ascending ramus is proportionally small. The diastema between the incisor and p4 measures 9.6 mm, about 19% of the total mandibular length. The masseteric fossa is gently concave, with the deepest area below the coronoid process, and extends mesially to the mid-length of m1. The coronoid process is distolabial to m2, with its mesial base placed below the alveolar margin of m2. The mandibular condyle does not have a neck and extends above the occlusal surface of the molars. The mandibular foramen lies below the distal part of m2 and opens into a concave area that continues distally to the deep pterygoid fossa.

Upper dentition **—** From the worn P1 to the erupting P4, it appears that the premolar eruption occurred in a mesiodistal sequence (Figs 3a and 4b). All premolars have two roots and smooth enamel. The crown of left P1 is damaged and that of the right one is deeply worn, but it is clear that P1 crown is significantly longer than wide with its distal half being wider than the mesial one. P2 has a major labial cusp and two lingual cusps; it is smaller and mesiodistally shorter than P1. In the right side dentition, a deeply worn and double-rooted tooth is positioned lateral to P2 (Figs 3a and 4d). This unusual location does not seem to be caused by distortion of the maxilla, although the maxilla was fractured in this region. This tooth is tentatively interpreted as an unshed deciduous premolar (?dP2). Distal to the right P2, P3 and P4 (dP4) are not preserved. Because of distortion of the skull, the right maxilla bearing M1-2 was mesially pushed so that the space between P2 and M1 is shortened. A concavity distal to P2 is presumably the alveolus for P3; it is not long enough as the alveoli for both P3 and P4 (dP4). The left P3 has two lingual cusps that are worn to form a continuous wear facet facing ventrolingually. The labial side of P3 also bears two major cusps, with the distal one being the largest of the tooth that is followed by a conule at its distal base. The tooth denoted as the left P4 sits distal to P3. It is not fully erupted and is only partly visible so that its complete morphology cannot be ascertained (Figs 3a and 4b,c). From the exposed portion, the crown of P4 appears similar to that of P3. The occlusal surface of P4 is lower than that of P3, but it already has a notable wear facet on its lingual side. This wear facet is continuous with that of the dP4. The tooth identified as dP4 is rectangular; it is shorter than M1 and wider than the mesial portion of M1. The tooth is deeply worn but its ridge-like distal cusps are still discernable. The mesiolingual part of dP4 was worn off or broken; otherwise dP4 would completely block P4 in occlusal view. The general outline and multicuspate crown of dP4 indicate a molariform tooth. The cusp formula of M1 is 6-7:7:5; it is narrow mesially and broad distally due to addition of the lingual row of cusps. The cusps of M1 are subequal, separated by narrow furrows, and have an opposing arrangement in which a lingual cusp aligns transversely with a labial cusp. After wear the labial cusps are taller than those of the medial and lingual rows. The lingual cusp row extends mesially to about two thirds of the tooth length. The cusp formula of M2 is 2:3:4. As in all multituberculates, M2 is distolingually displaced relative to M1.

Lower dentition **—** Relative to the robust mandible the lower incisor is slender with a subcircular cross-section of which the depth is slightly greater than the width. The enamel covers the labial surface of the tooth and wraps around to the medial and lateral sides, with a greater lateral extension. The incisor tip appears to be broken, but careful examination reveals that the tooth was intact but deeply worn so that the tip became blunt (Fig. 4a). This morphology indicates that the incisors were not sharpened at least during the late stage of life in this individual. The only lower premolar, p4, is blade-like, with an arcuate dorsal edge that is nearly at the same level with the occlusal surface of m1 (Fig. 4a,e). The p4 cusps and ridges on its lingual side are poorly developed and are more distinct on the distal half of the tooth. On the labial side, an oval wear facet extends from serration 2 to 8 on the distal half of p4. A small accessory cusp exists labial to serration 8 and bears a wear facet that is confluent mesially with the main wear facet. The cusp formula of m1 is 6:7. In contrast to M1, the m1 cusp arrangement is in an alternating fashion in which a lingual cusp aligns transversely with the furrow between successive labial cusps. The m1 labial cusp wear is more advanced than that of the lingual ones so that the lingual cusps are higher than the labial ones after wear. The m2 has a cusp formula 4:4., and is located on the lingual side of the coronoid process. Cusps on both rows of m2 decrease in size distally.

## Discussion

P4 Diphyodonty **—** *Yubaatar* presents the first evidence for diphyodonty of the ultimate upper premolar in multituberculates. The heavily worn tooth, denoted as dP4, is molariform and differs from any known ultimate upper premolar in multituberculates^2^. Because the ultimate premolar (P4) was most likely the last one to erupt, the new specimen favors a backward sequential replacement of premolars for Cretaceous-Paleogene multituberculates^20^,^39^. In multituberculates where the deciduous premolar and premolar are both known, such as *Nemegtbaatar gobiensis*^40^ and *Sinobaatar fuxinensis*^20^, the upper premolars are positioned immediately dorsal to the corresponding deciduous premolars and the morphologies of the two sets of teeth are generally similar^20^. In *Yubaatar*, however, the erupting P4 is positioned dorsal to the dP4 but its morphology is considerably different from the latter in being premolariform and much smaller.

A deciduous precursor for the ultimate upper premolar was unknown in multituberculates^5^,^17^,^39^. A deciduous p4 was reported for only one form, *Kuehneodon dietrichi*^3^, based on a single broken lower jaw that did not contain p4. Therefore, Wible and Rougier^17^, following others^39^,^41^,^42^, considered it possible that the ultimate upper and lower premolars of multituberculates are actually molars, i.e., unreplaced permanent teeth. If our interpretation of the dP4 is correct, the new specimen of *Yubaatar* shows that the ultimate premolars in multituberculates are probably deciduous teeth that are not replaced in lifetime. This would support the traditional view that multituberculates differ from other mammals in having only two molars. However, the possibility that the tooth we identified as P4 is “P3”, whereas P3 is “dP3”, cannot be ruled out. Under such an assumption, however, it is difficult to explain the deeply worn state of dP4 and its position ventral to “P3”. Also because the upper and lower molars in the holotype of *Yubaatar* bear considerable wear, the specimen must represent an adult individual; thus, it seems unlikely that at this stage of ontogeny there exist the erupting “P3” and “dP3” that bear similar wear. Moreover, given the extreme rarity of such cases presented so far only in *Yubaatar*, it is also possible that the condition reported here represents an abnormal tooth replacement in this individual.

Paleopathology **—** As shown in Fig. 5 and description therein, the holotype of *Yubaatar* displays an interesting and rare paleopathological incident, in which the right tibia was completely broken, dislocated, and then healed in natural condition. Compared to the total number of fossils, the percentage of vertebrate specimens possessing pathological abnormity is low^43^,^44^. Among Mesozoic vertebrates there were paleopathological studies on dinosaurs, pterosaurs and marine reptiles^44^, but there is no such report from mammals. At the level of a single individual, as the case of the specimen reported here, the paleopathological evidence provides a partial life history and adds a unique dimension to paleobiological reconstructions in understanding disease and injury in fossil organisms^45^. The completely healed tibia suggests that the trauma probably took place when the animal was young, because the young heal at a much greater rate than the old^46^ and fracturing of limbs in adult animals usually leads to death^47^. Healed fractures of bones are not uncommon among fossil vertebrates^47^,^48^,^49^, but a severe injury as reported here was rare and unknown in Mesozoic mammals. A similar case of limb bone fracture and fusion in fossil vertebrates was from the crocodilian *Toyotamaphimeia*^48^ in which the trauma was interpreted as being resulted from a single bite inflicted by a large carnivorous animal. In contrast, our examination revealed no bite or scratch mark on the broken tibia or other parts of the skeleton of *Yubaatar.* The broken tibia in the holotype of *Yubaatar* was probably due to an accident. The bone fracture of *Yubaatar* is oblique, suggesting that the force causing the fracture may be delivered at a right angle to the tibia, as so described in other studies^46^. Although causes vary, the consequences of bone fracture are similar^46^, including at least blood loss and displacement of bone fragments. Blood loss is from the damaged bone and periosteum and from overlying soft tissues that may have been injured, whereas displacement of bone fragments is caused by muscle spasm around the fracture. In the holotype of *Yubaatar*, even though the right fibula is normal, it did not prevent the displacement of the broken fragments of the tibia.

Healing of the broken tibia in the holotype of *Yubaatar* is evidenced by the rugged callus surrounding the fracture and by complete fusion of the broken elements. In addition to the fusion, the bone outgrowth (Fig. 5b) could have helped to reestablish the weight-supporting function of the tibia. Nonetheless, because of the shortening of the tibia the leg would not have worked normally. As an extinct group of mammals, it is difficult to know how long it took a multituberculate to heal the broken tibia, but a remodeled fracture around the dislocated fragments of the tibia would have taken a considerable time. Given that the tibia is the primary weight-bearing element, compared to the slim fibula, the animal may not have been able to use the right hind limb to support the body weight during the healing process. Although the individual survived the injury, the hind limb injury may have caused difficulty in escaping from natural predators when the animal was alive. However, unlike carnivorans in which fast and swift moves as essential predatory abilities affect hunting success^50^, an injured leg may not be so fatal for multituberculates that are generally considered as omnivorous or herbivorous^2^,^51^,^52^.

**Figure 6:**
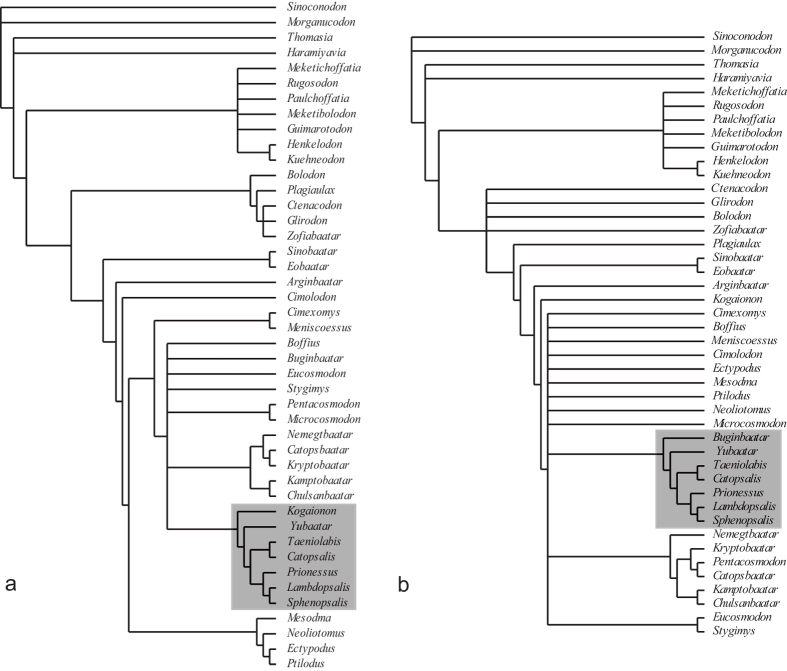
Phylogenetic relationship of *Yubaatar zhongyuanensis* within multituberculates. (**a**) The strict consensus of 18 equally most parsimonious trees (EMTs) with all characters unordered. Tree length (TL) = 349; consistency index (CI) = 0.4556; homoplasy index (HI) = 0.5444; retention index (RI) = 0.7552; rescaled consistency index (RC) = 0.3440. (**b**) Strict consensus of 69 EMTs with 19 characters ordered. TL = 421; CI = 0.4276; HI = 0.6223; RI = 0.7217; RC = 0.3086. See Material and Methods and the Supplementary Information for details of character list and phylogenetic analyses.

Phylogeny **—** Fig. 6 displays two strict consensus trees resulted from our phylogenetic analyses: one with all characters unordered (Fig. 6a), which is our preferred phylogeny, and the other with 19 characters ordered (Fig. 6b; see Methods and SI). All phylogenetic analyses placed *Yubaatar* as a taxon basal to Taeniolabidoidea (Fig. 6), a super-family that previously included only a monotypic family Taeniolabididae^2^,^6^,^53^ and consists of primarily five genera: *Taeniolabis*, *Catopsalis*, *Lambdopsalis*, *Prionessus* and *Sphenopsalis*. In the preferred phylogeny (Fig. 6a), the node for *Yubaatar* and taeniolabidoideans is supported by several characters, such as the ultimate upper premolar being small (transversely narrow) relative to M1, the posterior edge of the anterior zygomatic base distally positioned (lateral to M1), presence of a short postorbital process, and the anterior processes of the frontals deeply inserting between the nasals. In a recent study, Mao *et al.*^37^ re-established the family Lambdopsalidae, which was considered a subjective junior synonym of Taeniolabididae^54^,^55^, to include the Asian *Lambdopsalis* and *Sphenopsalis*, and possibly *Prionessus*. Our analyses support Lambdopsalidae as a valid taxon and further opt for including *Prionessus* in Lambdopsalidae. As the immediate outgroup of taeniolabidoideans, *Yubaatar* possesses primitively P1-3 and a blade-like p4, differing from all taeniolabidoideans. There is yet no evidence of a transitional condition between the four primitive upper premolar and the derived one upper premolar condition in taeniolabidoidean multituberculates. In addition, the phylogenies (Fig. 6) show that loss of p3 probably represents a derived condition within Cimolodonta and may have evolved independently more than once.

Because *Yubaatar* is latest Cretaceous in age, its phylogenetic relationship with taeniolabidoideans suggests a faunal interchange event of multituberculates between Asian and North American before the K-Pg boundary. The two clades, lambdopsalids and taeniolabidids, became specialized independently with their own lifestyles, as reflected by their dentitions (incisor and molar patterns) and cranial structures^37^,^56^. Among Eurasian multituberculates, *Yubaatar* is the largest known Mesozoic taxon, only next to the Paleocene *Sphenopsalis*^37^,^56^ in body size, contrasting most Mesozoic multituberculates that are usually of shrew to rat size^51^. Along with the new material of *Sphenopsalis*^37^, *Yubaatar* shows again that multituberculate dental complexity rose with generic richness and body mass across the K-Pg transition and that an adaptive shift towards increased herbivory was little affected by the K-Pg mass extinction^51^.

## Methods

In the description, we follow the convention to use I, C, P, M/ i, c, p, m for upper and lower incisors, canine, premolars, and molars respectively. Traditionally, the upper premolars of Late Cretaceous and Tertiary multituberculates are designated as P1-P4^2^, which is followed here, although these teeth may be homologous to P1–P3 and P5 of “Plagiaulacida”, respectively^57^. In coding premolar characters, we treat the only lower premolar in *Yubaatar* as p4. Tooth measurements were made using a Mitutoyo digital caliber and presented in Fig. 4 caption.

For phylogenetic analyses, we adopted the data matrix from Mao *et al.*^37^, which was built up from several studies (see SI). To make our results comparable to previous work, we run the phylogenetic analyses using the same methods employed by other researchers^8^,^37^ (see SI). Because many multituberculate species are based on fragmentary material, the terminal taxa used for phylogenetic analyses of multituberculates have to be at the generic level^6^,^37^,^58^,^59^. The phylogenetic analyses were performed using PAUP^60^ with a heuristic search criterion. The search settings for the preferred tree (Fig. 6a) are as the following: The optimality criterion is parsimony. All characters are unordered and equally weighted. Gaps are treated as “missing” and multistate taxa interpreted as polymorphism. A random stepwise addition sequence was used with 1000 replicates. Number of trees held at each step during stepwise addition is 10 and the branch-swapping algorithm is tree-bisection-reconnection (TBR). The strict consensus with 19 characters being ordered is presented in Fig. 6b. Other results (50% majority consensus trees) are presented in the Supporting Information (SI). The data matrix, the character list and the PAUP logs for all the four trees are provided in SI.

## Additional Information

**How to cite this article**: Xu, L. *et al.* Largest known Mesozoic multituberculate from Eurasia and implications for multituberculate evolution and biology. *Sci. Rep.* **5**, 14950; doi: 10.1038/srep14950 (2015).

## Supplementary Material

Supplementary Information
