# Supplementary material for: Largest known Mesozoic multituberculate from Eurasia and implications for multituberculate evolution and biology
Source: Sci Rep. 2015 Oct 22;5:14950. doi: 10.1038/srep14950 (PMC4615031; doi:10.1038/srep14950)
Supplement: Supplementary Information [file srep14950-s1.pdf]

Supplementary Information

Largest known Mesozoic multituberculate from Eurasia and implications for multituberculate evolution and biology

Li Xu[1], Xingliao Zhang[1], Hanyong Pu[1], Songhai Jia[1], Jiming Zhang[1], Junchang Lü[2], Jin Meng[3*]

[1]Henan Geological Museum, Zhengzhou, Henan 450016, China
[2]Institute of Geology, Chinese Academy of Geological Sciences, Beijing 10037, China
[3]Division of Paleontology, American Museum of Natural History, Central Park West at 79th Street, New York, New York 10024, USA.

[*]To whom correspondence should be addressed. E-mail: jmeng@amnh.org.

I. Data Matrix

The primary data matrix is from Mao et al. (in press) with addition of *Yubaatar zhongyuanensis* (see more introduction in Methods and Material and in Phylogenetic Analyses).

Taxa	1	2	3	4	5	6	7	8	9	10	11	12	13	14	15	16	17	18	19	20	21	22	23
Sinoconodon	0	0	0	0	0	0	0	0	0	0	0	0	0	0	0	0	0	0	0	0	0	0	0
Morganucodon	0	0	0	0	0	0	0	0	0	0	0	0	0	0	0	0	0	0	0	0	0	0	0
Thomasia	?	?	?	?	?	?	?	?	?	?	0	1	?	?	0	0	0	?	0	0	?	?	?
Haramiyavia	0	0	0	?	1	0	0	0	0	0	0	1	0	0	0	0	0	1	0	0	0	1	0
Meketichoffatia	?	?	?	?	?	?	?	?	?	?	?	?	?	?	?	?	?	?	?	?	?	1	1
Henkelodon	?	?	?	?	?	?	?	?	?	?	?	?	?	?	?	?	1	0	1	2	0	2	?
Rugosodon	1	1	1	?	1	?	0	1	0	1	1	1	1	1	0	0	1	0	1	1	0	1	1
Paulchoffatia	1	1	1	?	1	0	0	1	0	1	1	1	0	1	0	0	?	?	?	?	?	?	?
Meketibolodon	1	1	1	1	1	0	0	1	0	1	1	1	0	1	0	0	?	?	?	?	?	?	?
Guimarotodon	1	1	1	1	1	0	0	1	0	1	1	1	0	1	0	0	?	?	?	?	?	?	?
Kuehneodon	1	1	1	0	1	0	0	1	0	1	1	1	1	1	0	0	1	0	1	2	0	1	1
Ctenacodon	1	1	1	1	0	?	1	1	1	1	1	1	1	1	0	0	1	0	1	1	0	1	?
Glirodon	1	1	1	1	0	0	1	1	1	1	1	1	1	1	0	1	1	0	1	1	0	1	0
Bolodon	1	1	1	1	1	?	1	1	?	?	1	1	1	?	0	0	?	?	?	?	?	1	?
Plagiaulax	1	1	1	1	0	0	1	1	1	1	1	1	1	1	0	0	?	?	?	?	?	?	?
Zofiabaatar	1	1	1	1	0	0	1	1	1	1	1	1	?	1	0	?	?	?	?	?	?	?	?
Sinobaatar	1	1	1	1	0	?	1	1	0	1	1	1	1	1	0	0	2	1	0	1	?	2	?
Eobaatar	1	1	?	1	?	?	?	1	?	1	1	1	1	1	0	2	?	?	?	?	?	?	?
Arginbaatar	?	1	2	1	0	?	?	1	?	?	1	1	1	1	0	0	?	?	?	?	?	?	?
Cimexomys	1	1	2	1	?	?	?	?	?	1	1	1	1	1	0	0	?	?	?	?	?	?	?
Boffius	?	?	?	?	?	?	?	?	?	?	?	?	?	?	0	1	2	?	?	?	?	?	?
Meniscoessus	1	1	2	1	1	?	0	1	?	1	1	1	1	1	0	0	2	1	1	1	1	2	?
Buginbaatar	1	1	1	1	1	0	0	1	?	?	1	1	?	1	0	0	?	?	?	?	?	?	?
Cimolodon	1	1	2	1	0	?	0	1	?	1	1	1	1	1	?	0	?	?	?	?	?	2	?
Ectypodus	1	1	2	1	0	?	0	1	1	1	1	1	1	1	1	0	2	1	0	1	1	2	?
Mesodma	1	1	2	1	1	?	0	1	?	1	1	1	1	1	1	0	2	?	1	1	1	2	?
Ptilodus	1	1	2	1	0	0	0	1	0	1	1	1	1	1	1	0	2	1	0	1	1	2	?
Neoliotomus	1	1	2	1	1	?	0	1	?	1	1	1	1	1	0	2	?	?	0	?	?	?	?
Pentacosmodon	1	1	2	?	0	0	1	1	1	1	1	1	1	1	0	1	?	?	?	?	?	?	?
Catopsbaatar	1	1	2	1	0	0	1	1	1	1	1	1	1	1	0	2	2	1	0	1	2	2	?
Kamptobaatar	1	1	2	1	1	1	0	1	1	1	1	1	1	1	0	1	2	1	0	1	2	2	?
Chulsanbaatar	1	1	2	1	1	0	1	1	0	1	1	1	1	1	0	2	2	1	0	1	2	2	?
Kryptobaatar	1	1	2	1	1	0	0	1	0	1	1	1	1	1	0	2	2	1	0	1	2	2	?
Nemegtbaatar	1	1	2	1	1	0	0	1	0	1	1	1	1	1	0	2	2	1	0	1	2	2	?
Eucosmodon	1	1	2	1	1	?	1	1	0	1	1	1	1	1	0	2	2	?	1	1	?	2	?
Stygimys	1	1	1	1	1	0	0	1	0	1	1	1	1	1	0	2	2	1	1	1	1	2	?
Microcosmodon	1	1	2	?	0	?	0	1	1	1	1	1	1	1	0	1	2	1	1	1	1	2	?
Kogaionon	?	?	?	?	?	?	?	?	?	?	?	?	?	?	?	?	2	1	0	1	1	2	?
Taeniolabis	1	1	2	1	0	0	0	1	1	1	1	1	1	1	0	2	2	0/1	1	0	0	2	?
Lambdopsalis	1	1	2	1	0	1	1	1	1	1	1	1	1	1	0	2	2	0	0	0	0	2	?
Sphenopsalis	1	1	?	1	?	?	?	1	?	1	1	1	1	1	0	2	2	0	0	0	0	2	?
Catopsalis	1	1	2	1	0	0	0	1	0/1	1	1	1	1	1	0	2	2	0/1	1	0	0	2	?
Prionessus	1	1	2	1	?	?	?	1	?	1	1	1	1	1	0	2	2	?	?	?	?	2	?
Yubaatar	1	1	1	1	0	0	0	1	1	1	1	1	1	1	0	2	?	?	?	?	?	2	?

Taxa	24	25	26	27	28	29	30	31	32	33	34	35	36	37	38	39	40	41	42	43	44	45	46
Sinoconodon	0	0	0	0	0	2	?	0	0	0	0	0	0	0	0	?	0	0	0	?	0	0	1
Morganucodon	0	0	0	0	0	2	?	0	0	0	0	0	0	0	0	?	0	0	0	?	0	0	1
Thomasia	?	?	3	0	0	2	0	0	1	?	?	0	0	0	0	?	0	0	1	?	0	0	1
Haramiyavia	0	?	?	?	?	?	?	?	?	0	0	0	0	0	0	?	0	0	0	?	0	0	?
Meketichoffatia	?	0	0	0	1	0	0	0	0	?	?	?	?	?	?	?	?	?	?	?	?	?	?
Henkelodon	?	0	0	0	1	0	0	0	0	?	?	?	?	?	?	?	?	?	?	?	?	?	?
Rugosodon	1	0	0	0	?	0	0	?	?	0	0	0	1	?	1	?	0	1	1	0	0	1	?
Paulchoffatia	1	?	?	?	?	?	?	?	?	0	0	0	1	0	1	0	0	1	1	0	0	1	0
Meketibolodon	1	?	?	?	?	?	?	?	?	0	0	0	1	0	1	0	0	1	1	0	0	1	0
Guimarotodon	1	?	?	?	?	?	?	?	?	0	0	0	1	0	1	0	0	1	1	0	0	1	0
Kuehneodon	1	0	1	0	1	0	0	0	0	0	0	0	1	0	1	0	0	1	1	0	0	1	0
Ctenacodon	1	0	0	0	1	0	1	0	0	0	0	0	1	0	1	1	0	1	1	0	0	1	1
Glirodon	1	0	0	0	1	0	1	0	0	0	0	0	?	0	1	1	0	1	1	0	0	1	1
Bolodon	1	?	0	0	1	0	1	0	0	0	0	0	1	0	2	1	0	1	1	0	0	1	1
Plagiaulax	1	?	?	?	?	?	?	?	?	1	0	0	1	0	2	1	0	1	1	1	0	?	1
Zofiabaatar	?	?	?	?	?	?	?	?	?	0	0	0	1	0	1	1	0	1	1	0	0	1	1
Sinobaatar	1	0	0	0	0	0	2	0	1	1	0	0	1	1	2	1	0	1	2	1	1	1	2
Eobaatar	1	?	0	0	0	0	0	0	1	1	0	0	1	0	2	1	0	1	2	1	1	?	2
Arginbaatar	1	?	0	0	0	0	0	0	1	1	0	0	0	1	2	1	1	1	2	2	1	1	2
Cimexomys	1	?	1	0	0	1	2	1	2	1	0	0	0	1	3	1	1	1	2	1	1	0	3
Boffius	?	?	?	?	?	?	?	1	1	?	?	?	?	?	?	?	?	?	?	?	?	?	?
Meniscoessus	1	2	1	0	0	2	2	0	2	1	1	0	0	1	3	1	1	1	2	1	1	0	3
Buginbaatar	?	?	?	?	0	?	2	1	3	1	1	1	?	?	?	?	?	1	2	0	0	0	?
Cimolodon	1	?	1	0	0	1	2	1	1	1	1	0	0	1	3	1	1	1	2	2	1	1	2
Ectypodus	1	0	1	0	0	1	2	2	1	1	1	0	0	1	3	1	1	1	2	2	1	1	3
Mesodma	1	?	1	0	0	?	2	1	1	1	1	0	0	1	3	1	1	1	2	2	1	1	3
Ptilodus	1	0	1	0	0	0	2	2	1	1	1	0	0	1	3	1	1	1	2	2	1	1	3
Neoliotomus	1	?	1	0	0	1	2	2	1	1	1	0	0	1	3	1	1	1	2	2	1	1	3
Pentacosmodon	1	?	?	?	?	?	?	0	1	1	1	1	0	?	?	?	1	1	2	0	1	0	?
Catopsbaatar	1	0	2	0	0	2	2	1	1	1	1	0	0	1	3	1	1	1	1	0	0	0	3
Kamptobaatar	1	0	1	0	0	1	2	1	1	1	1	0	0	1	3	1	1	1	2	1	1	0	3
Chulsanbaatar	1	0	1	0	0	1	2	1	1	1	1	0	0	1	3	1	1	1	2	1	1	0	3
Kryptobaatar	1	0	1	0	0	1	2	1	1	1	1	0	0	1	3	1	1	1	2	1	1	0	3
Nemegtbaatar	1	1	1	0	0	1	2	1	1	1	1	0	0	1	3	1	1	1	2	1	1	0	3
Eucosmodon	1	?	2	0	0	?	2	?	1	1	1	1	?	?	?	?	?	1	2	2	1	0	?
Stygimys	1	1	1	0	0	1	2	2	1	1	1	1	?	?	?	?	?	1	2	2	1	0	?
Microcosmodon	1	0	1	0	0	1	2	1	1	1	1	0	0	1	2	1	1	1	2	0	1	0	?
Kogaionon	?	1	1	0	0	0	3	0	1	?	?	?	?	?	?	?	?	?	?	?	?	?	?
Taeniolabis	1	2	3	1	0	2	2/3	0	1	1	1	1	?	?	?	?	?	0	3	?	0	0	?
Lambdopsalis	1	2	3	1	0	2	3	0	1	1	1	1	?	?	?	?	?	0	3	?	0	0	?
Sphenopsalis	1	2	3	1	0	2	3	0	1	1	1	1	?	?	?	?	?	0	3	?	0	0	?
Prionessus	1	?	3	0/1	0	2	3	0	1	1	1	1	?	?	?	?	?	0	3	?	0	0	?
Catopsalis	1	2	3	1	0	2	2/3	1	1	1	1	1	?	?	?	?	?	0	3	?	0	0	?
Yubaatar	1	?	1	0	0	1	2/3	0	2/3	1	1	1	?	?	?	?	?	1	0	2	1	0	0

Taxa	47	48	49	50	51	52	53	54	55	56	57	58	59	60	61	62	63	64	65	66	67	68	69
Sinoconodon	0	0	?	?	0	0	0	0	?	0	?	?	?	0	1	0	0	?	?	0	0	0	0
Morganucodon	0	0	0	0	0	0	0	0	?	0	?	?	?	0	1	0	0	?	?	0	0	0	0
Thomasia	0	0	0	0	?	?	1	0	?	0	0	?	?	0	1	1	3	0	0	0	0	?	2
Haramiyavia	0	0	0	0	0	0	1	0	?	0	0	?	?	0	1	1	3	0	0	1	0	1	2
Meketichoffatia	?	?	?	1	1	?	?	?	0	0	0	?	?	1	?	1	1	?	?	?	?	?	?
Henkelodon	?	?	?	1	1	?	?	?	0	1	0	?	?	1	?	1	2	?	?	?	?	?	?
Rugosodon	?	1	1	1	1	1	1	0	0/1	1	0	?	?	1	0	1	1	1	1	2	0	2	2
Paulchoffatia	2	1	1	?	?	1	1	0	0	?	?	?	?	?	?	?	?	?	?	?	0	2	2
Meketibolodon	3	?	?	?	?	?	?	?	0	?	?	?	?	?	?	?	?	?	?	?	?	2	?
Guimarotodon	3	1	1	?	?	1	1	0	?	?	?	?	?	?	?	?	?	?	?	?	0	2	2
Kuehneodon	2	1	1	1	2	1	1	0	0	1	0	?	?	1	0	1	2	1	1	2	0	2	2
Ctenacodon	2	1	1	1	1	1	0	1	1	1	1	0	0	1	0	2	3	0	0	1	0	1	1
Glirodon	2	1	1	1	1	1	0	1	0	1	0	?	?	1	0	2	3	0	0	1	0	1	1
Bolodon	2	?	1	1	1	1	0	1	0	1	1	0	0	1	0	2	3	0	0	1	0	?	1
Plagiaulax	2	?	1	?	?	1	0	1	?	?	?	?	?	?	?	?	?	?	?	1	0	1	1
Zofiabaatar	2	3	1	?	?	1	0	1	?	?	?	?	?	?	?	?	?	?	?	1	0	?	1
Sinobaatar	1	2	1	1	1	2	0	1	1	1	1	1	0	1	1	2	3	0	0	1	0	1	1
Eobaatar	1	?	1	1	1	2	0	1	0	1	1	1	0	1	1	2	3	0	0	1	0	1	1
Arginbaatar	0	3	1	1	1	2	0	1	0	1	1	0	0	1	0	2	3	0	0	1	0	1	1
Cimexomys	0	2	1	1	2	2	0	1	2	1	1	2	2	1	1	2	3	0	0	1	1	1	1
Boffius	?	?	1	?	?	?	?	?	3	?	1	2	3	1	?	2	3	?	?	1	?	?	?
Meniscoessus	0	0	1	1	2	2	0	1	2/3	1	1	2	3	1	2	2	3	0	0	1	1	?	1
Buginbaatar	0	0	1	1	?	2	0	1	3	1	1	1	3	1	2	2	3	0	0	1	2	?	1
Cimolodon	0	1	1	1	2	2	0	1	2	1	1	2	2	1	2	2	3	0	0	1	2	1	1
Ectypodus	0	3	1	1	2	2	0	1	2/3	1	1	1	3	1	1	2	3	1	0	1	2	1	1
Mesodma	0	1	1	1	2	2	0	1	2	1	1	1	3	1	1	2	3	0	0	1	2	1	1
Ptilodus	0	2	1	1	2	2	0	1	2/3	1	1	1	3	1	1	2	3	1	0	1	2	1	1
Neoliotomus	0	3	1	1	2	2	0	1	3	1	1	2	3	1	1	2	3	0	0	1	2	1	1
Pentacosmodon	1	0	1	1	?	2	0	1	?	?	?	1	?	?	?	?	?	?	?	?	0	1	1
Catopsbaatar	0	2	1	1	3	2	0	1	2	1	1	2	3	1	0	2	3	0	0	1	0	1	1
Kamptobaatar	0	1	1	1	2	2	0	1	1	1	1	0	1	1	1	2	3	0	0	1	0	1	1
Chulsanbaatar	0	1	1	1	2	2	0	1	1	1	1	0	1	1	1	2	3	0	0	1	0	1	1
Kryptobaatar	0	1	1	1	2	2	0	1	1	1	1	0	2	1	2	2	3	0	0	1	0	1	1
Nemegtbaatar	0	1	1	1	2	2	0	1	1/2	1	1	2	2	1	2	2	3	0	0	1	0	1	1
Eucosmodon	0	1	1	1	2	2	0	1	2	1	1	?	?	1	?	2	3	?	?	1	1	1	1
Stygimys	1	1	1	1	2	2	0	1	2	1	1	1	2/3	1	1	2	3	0	0	1	2	1	1
Microcosmodon	1	2	1	1	2	2	0	1	2	1	1	1	3	1	1	2	3	0	0	1	2	1	1
Kogaionon	?	?	?	1	2	?	?	?	1	1	1	2	2	1	0	2	3	0	0	1	?	?	?
Taeniolabis	1	0	1	1	3	2	0	1	3	1	1	2	3	1	1/2	2	4	0	2	1	2	1	1
Lambdopsalis	0	0	1	1	3	2	2	1	3	1	1	2	3	1	0	1	3	0	0	1	0	2	2
Sphenopsalis	0	0	1	1	3	2	2	1	3	1	1	2	3	1	0	1	3	0	0	1	0	2	2
Prionessus	0	0	1	1	3	2	0	1	3	1	1	2	3	1	0	1/2	3	0	0	1	0	1	1
Catopsalis	1	0	1	1	3	2	0	1	3	1	1	2	3	1	1	2	4	0	1/2	1	0/1	1	1
Yubaatar	1	0	1	1	2	2	0	1	2	1	1	2	3	1	1	2	3	0	0	1	2	1	1

Taxa	70	71	72	73	74	75	76	77	78	79	80	81	82	83	84	85	86	87	88	89	90	91	92
Sinoconodon	**?**	**?**	**?**	**?**	**?**	**?**	**0**	**0**	**0**	**0**	**0**	**0**	**0**	**0**	**1**	**0**	**0**	**0**	**0**	**1**	**0**	**0**	**0**
Morganucodon	**?**	**?**	**?**	**?**	**?**	**?**	**0**	**0**	**1**	**1**	**0**	**0**	**0**	**0**	**0**	**0**	**0**	**0**	**0**	**1**	**0**	**0**	**0**
Thomasia	**0**	**0**	**2**	**0**	**0**	**0**	**0**	**?**	**1**	**?**	**?**	**?**	**?**	**?**	**?**	**?**	**?**	**?**	**?**	**?**	**?**	**?**	**?**
Haramiyavia	**0**	**0**	**2**	**0**	**0**	**0**	**0**	**1**	**1**	**?**	**?**	**?**	**?**	**?**	**?**	**?**	**?**	**0**	**?**	**?**	**?**	**?**	**?**
Meketichoffatia	**?**	**?**	**?**	**?**	**?**	**?**	**1**	**?**	**1**	**?**	**0**	**0**	**0**	**0**	**?**	**?**	**0**	**0**	**?**	**0**	**0**	**?**	**?**
Henkelodon	**?**	**?**	**?**	**?**	**?**	**?**	**1**	**?**	**1**	**1**	**0**	**?**	**?**	**?**	**?**	**?**	**?**	**?**	**?**	**?**	**?**	**?**	**?**
Rugosodon	**1**	**1**	**0**	**0**	**0**	**1**	**1**	**1**	**1**	**1**	**0**	**?**	**?**	**?**	**?**	**0**	**?**	**?**	**?**	**0**	**?**	**?**	**?**
Paulchoffatia	**1**	**1**	**0**	**0**	**0**	**2**	**1**	**1**	**1**	**?**	**?**	**?**	**?**	**?**	**?**	**?**	**?**	**?**	**?**	**?**	**?**	**?**	**?**
Meketibolodon	**?**	**?**	**0**	**?**	**?**	**2**	**1**	**?**	**1**	**?**	**?**	**?**	**?**	**?**	**?**	**?**	**?**	**?**	**?**	**?**	**?**	**?**	**?**
Guimarotodon	**1**	**1**	**0**	**0**	**0**	**2**	**1**	**1**	**1**	**?**	**?**	**?**	**?**	**?**	**?**	**?**	**?**	**?**	**?**	**?**	**?**	**?**	**?**
Kuehneodon	**1**	**1**	**1**	**0**	**0**	**1/2**	**1**	**1**	**1**	**1**	**0**	**?**	**?**	**?**	**?**	**?**	**?**	**0**	**?**	**?**	**?**	**?**	**?**
Ctenacodon	**0**	**0**	**1**	**1**	**0**	**0**	**0**	**0**	**1**	**?**	**0**	**?**	**?**	**0**	**?**	**?**	**0**	**?**	**?**	**?**	**?**	**?**	**?**
Glirodon	**0**	**0**	**1**	**1**	**0**	**0**	**0**	**0**	**1**	**?**	**0**	**0**	**1**	**0**	**1**	**1**	**0**	**0**	**?**	**?**	**0**	**?**	**0**
Bolodon	**?**	**?**	**1**	**1**	**0**	**0**	**1**	**0**	**1**	**?**	**0**	**0**	**?**	**?**	**?**	**1**	**?**	**?**	**?**	**?**	**?**	**?**	**?**
Plagiaulax	**0**	**0**	**1**	**1**	**?**	**0**	**0**	**0**	**1**	**?**	**?**	**?**	**?**	**?**	**?**	**?**	**?**	**?**	**?**	**?**	**?**	**?**	**?**
Zofiabaatar	**0**	**0**	**1**	**1**	**0**	**0**	**0**	**0**	**1**	**?**	**?**	**?**	**?**	**?**	**?**	**?**	**?**	**?**	**?**	**?**	**?**	**?**	**?**
Sinobaatar	**1**	**0**	**1**	**1**	**0**	**0**	**1**	**0**	**1**	**1**	**1**	**0**	**1**	**?**	**1**	**0**	**1**	**0**	**?**	**?**	**?**	**?**	**?**
Eobaatar	**1**	**0**	**1**	**1**	**0**	**0**	**1**	**1**	**1**	**?**	**?**	**?**	**?**	**?**	**?**	**?**	**?**	**?**	**?**	**?**	**?**	**?**	**?**
Arginbaatar	**0**	**0**	**1**	**1**	**0**	**0**	**0**	**1**	**1**	**?**	**0**	**?**	**?**	**?**	**?**	**?**	**?**	**?**	**?**	**?**	**?**	**?**	**?**
Cimexomys	**0**	**0**	**1**	**1**	**0**	**0**	**1**	**1**	**1**	**?**	**1**	**?**	**?**	**?**	**1**	**0**	**1**	**?**	**?**	**?**	**?**	**?**	**?**
Boffius	**?**	**?**	**?**	**1**	**?**	**0**	**0**	**1**	**1**	**?**	**?**	**?**	**?**	**?**	**?**	**?**	**?**	**?**	**?**	**?**	**?**	**?**	**?**
Meniscoessus	**0**	**0**	**2**	**1**	**1**	**0**	**1**	**1**	**1**	**?**	**0**	**2**	**1**	**0**	**?**	**0**	**1**	**?**	**?**	**?**	**?**	**?**	**?**
Buginbaatar	**0**	**0**	**1**	**1**	**0**	**0**	**0**	**?**	**1**	**?**	**?**	**?**	**?**	**?**	**?**	**?**	**?**	**?**	**?**	**?**	**?**	**?**	**?**
Cimolodon	**0**	**0**	**2**	**1**	**0**	**0**	**1**	**1**	**1**	**1**	**?**	**?**	**?**	**?**	**?**	**?**	**?**	**?**	**?**	**?**	**?**	**?**	**?**
Ectypodus	**0**	**0**	**2**	**1**	**0**	**0**	**1**	**2**	**1**	**1**	**1**	**1**	**?**	**2**	**?**	**0**	**1**	**0**	**?**	**0**	**?**	**?**	**0**
Mesodma	**0**	**0**	**1**	**1**	**0**	**0**	**1**	**2**	**1**	**1**	**1**	**?**	**?**	**?**	**?**	**?**	**?**	**?**	**?**	**?**	**?**	**?**	**?**
Ptilodus	**0**	**0**	**2**	**1**	**0**	**0**	**1**	**2**	**1**	**1**	**1**	**1**	**1**	**0**	**2**	**0**	**0**	**0**	**?**	**0**	**0**	**0**	**0**
Neoliotomus	**0**	**0**	**2**	**1**	**1**	**0**	**1**	**2**	**1**	**?**	**?**	**?**	**?**	**?**	**?**	**?**	**?**	**?**	**?**	**?**	**?**	**?**	**?**
Pentacosmodon	**0**	**0**	**1**	**1**	**0**	**0**	**0**	**1**	**1**	**?**	**?**	**?**	**2**	**?**	**?**	**?**	**?**	**?**	**?**	**?**	**?**	**?**	**?**
Catopsbaatar	**0**	**0**	**1**	**1**	**0**	**0**	**0**	**1**	**1**	**?**	**1**	**0**	**2**	**2**	**2**	**0**	**1**	**1**	**1**	**1**	**1**	**1**	**0**
Kamptobaatar	**0**	**0**	**1**	**1**	**0**	**0**	**0**	**1**	**1**	**?**	**1**	**0**	**2**	**0**	**3**	**2**	**0**	**1**	**0**	**0**	**1**	**1**	**0**
Chulsanbaatar	**0**	**0**	**1**	**1**	**0**	**0**	**0**	**1**	**1**	**1**	**1**	**0**	**2**	**0**	**2**	**2**	**0**	**1**	**0**	**0**	**1**	**1**	**0**
Kryptobaatar	**0**	**0**	**1**	**1**	**0**	**0**	**0**	**1**	**1**	**1**	**1**	**0**	**2**	**2**	**2**	**1**	**0**	**1**	**1**	**1**	**1**	**1**	**0**
Nemegtbaatar	**0**	**0**	**1**	**1**	**0**	**0**	**0**	**1**	**1**	**1**	**1**	**2**	**2**	**0**	**3**	**1**	**0**	**1**	**0**	**0**	**1**	**1**	**0**
Eucosmodon	**0**	**0**	**2**	**1**	**0**	**0**	**0**	**1**	**1**	**?**	**1**	**?**	**?**	**?**	**?**	**?**	**?**	**?**	**?**	**?**	**?**	**?**	**?**
Stygimys	**0**	**0**	**1**	**1**	**0**	**0**	**0**	**1**	**1**	**?**	**1**	**2**	**1**	**0**	**?**	**2**	**0**	**?**	**?**	**?**	**?**	**?**	**?**
Microcosmodon	**0**	**0**	**1**	**1**	**0**	**0**	**0**	**?**	**1**	**1**	**1**	**0**	**1**	**1**	**?**	**0**	**0**	**0**	**?**	**?**	**?**	**?**	**?**
Kogaionon	**?**	**?**	**?**	**?**	**?**	**?**	**0**	**?**	**1**	**?**	**1**	**0**	**1**	**1**	**3**	**0**	**0**	**0**	**0**	**0**	**0**	**0**	**0**
Taeniolabis	**0**	**0**	**2**	**0**	**2**	**0**	**0**	**1**	**1**	**1**	**1**	**0**	**1**	**1**	**3**	**0**	**?**	**0**	**?**	**0**	**2**	**0**	**1**
Lambdopsalis	**0**	**0**	**1**	**1**	**1**	**0**	**0**	**1**	**1**	**1**	**1**	**0**	**1**	**1**	**3**	**0**	**1**	**0**	**1**	**0**	**2**	**0**	**1**
Sphenopsalis	**0**	**0**	**1**	**1**	**1**	**0**	**0**	**1**	**1**	**?**	**?**	**0**	**1**	**?**	**?**	**?**	**1**	**?**	**1**	**?**	**2**	**0**	**?**
Prionessus	**0**	**0**	**1**	**0**	**0**	**0**	**0**	**1**	**1**	**?**	**?**	**0**	**1**	**?**	**?**	**?**	**1**	**?**	**?**	**?**	**?**	**?**	**?**
Catopsalis	**0**	**0**	**1**	**0/1**	**2**	**0**	**0**	**1**	**1**	**?**	**?**	**?**	**?**	**?**	**?**	**?**	**?**	**?**	**?**	**?**	**?**	**?**	**?**
Yubaatar	**0**	**0**	**2**	**0**	**0**	**0**	**0**	**?**	**1**	**1**	**1**	**1/2**	**1**	**4**	**0**	**1**	**0**	**1**	**?**	**2**	**0**	**0**	**1**

Taxa	93	94	95	96	97	98	99	100	101	102
Sinoconodon	0	0	0	?	0	0	0	0	0	0
Morganucodon	0	0	0	?	0	0	0	0	0	0
Thomasia	?	?	?	?	?	?	?	?	?	?
Haramiyavia	?	?	?	?	?	?	?	?	?	?
Meketichoffatia	?	?	1	1	0	0	0	?	1	0
Henkelodon	?	?	?	?	?	?	?	?	?	?
Rugosodon	1	1	?	?	?	?	?	?	?	?
Paulchoffatia	?	?	?	?	?	?	?	?	?	?
Meketibolodon	?	?	?	?	?	?	?	?	?	?
Guimarotodon	?	?	?	?	?	?	?	?	?	?
Kuehneodon	?	?	?	?	?	?	?	?	?	?
Ctenacodon	?	?	?	?	?	?	?	?	?	?
Glirodon	?	?	1	?	?	?	?	?	?	?
Bolodon	?	?	?	?	?	?	?	?	?	?
Plagiaulax	?	?	?	?	?	?	?	?	?	?
Zofiabaatar	?	?	?	?	?	?	?	?	?	?
Sinobaatar	1	?	?	?	?	?	?	?	?	?
Eobaatar	?	?	?	?	?	?	?	?	?	?
Arginbaatar	?	?	?	?	?	?	?	?	?	?
Cimexomys	?	?	?	?	?	?	?	?	?	?
Boffius	?	?	?	?	?	?	?	?	?	?
Meniscoessus	?	0	1	?	?	?	?	?	?	?
Buginbaatar	?	?	?	?	?	?	?	?	?	?
Cimolodon	?	?	?	?	?	?	?	?	?	?
Ectypodus	?	?	1	1	0	0	1	?	1	0
Mesodma	?	?	?	?	?	?	?	?	?	?
Ptilodus	0	0	1	0	0	0	1	0	1	0
Neoliotomus	?	?	?	?	?	?	?	?	?	?
Pentacosmodon	?	?	?	?	?	?	?	?	?	?
Catopsbaatar	1	1	0	?	1	1	0	0	0	1
Kamptobaatar	1	1	1	0	1	0	0	0	1	0
Chulsanbaatar	1	1	1	1	1	0	0	0	1	0
Kryptobaatar	1	1	1	1	1	1	0	0	0	1
Nemegtbaatar	1	1	1	0	1	0	0	0	1	0
Eucosmodon	?	?	?	?	?	?	?	?	?	?
Stygimys	?	1	1	?	?	?	?	?	?	?
Microcosmodon	?	?	1	?	?	?	1	?	?	?
Kogaionon	?	?	1	?	1	?	1	?	1	0
Taeniolabis	0	0	?	?	?	?	?	1	1	1
Lambdopsalis	0	0	1	?	1	1	1	1	1	1
Sphenopsalis	?	?	1	?	?	1	?	?	?	?
Prionessus	?	?	?	?	?	?	?	?	?	?
Catopsalis	?	?	?	?	?	?	?	?	?	?
Yubaatar	1	0	?	?	1	1	1	1	?	?

III. Character list

We adopted the character list from Mao et al. (in press), which incorporated morphological characters from various sources concerning multituberculates and their relatives. The dataset of Mao et al. consists of 43 taxa and 102 characters and contains all taxa of Taeniolabidoidea, including Taeniolabididae Granger and Simpson, 1929 (*Taeniolabis* Cope, 1882; *Catopsalis* Cope, 1882), Lambdopsalidae Chow and Qi, 1978 (*Lambdopsalis* Chow and Qi, 1978; *Sphenopsalis* Matthew, Granger, and Simpson, 1928) and *Prionessus* (family incertae sedis). These taxa are highly relevant to *Yubaatar*, given the similarity in their morphologies. In addition, with the new data from *Sphenopsalis*, Mao et al. were able to modify, clarify and correct some characters and character codings for some taxa that were used in previous works. In the character list below, we will list our coding for *Yubaatar* in bold.

Following Mao et al. (in press), we have kept the original resources of characters compiled in the list, using the following abbreviations: KJ-H ## - characters from Kielan-Jaworowska and Hurum (2001), Luo## - characters from Luo et al. (2002)'s mammaliaform character list, R## - characters from Rougier et al. (1997), W## - characters from Weil (1998). C## - characters on M2 from Cifelli et al. (2013). Y## - characters introduced by Yuan et al. (2013). The primary reference for the work of Mao et al. was from Yuan et al. (2013).

To make the work comparable to previous work, we run the phylogenetic analyses using the same methods used by other workers (Yuan et al., 2013; Mao et al., in press). The results we obtained include the strict consensus and 50% majority consensus trees based on all characters unordered and on characters with19 ordered. The ordered characters are marked in the character list and explained in Mao et al. (in press).

Mandibular Features

1. (Luo1) Postdentary trough: (0) Present; (1) Absent.

***Yubaatar* = 1**

2. (Luo2) Meckel's sulcus presence: (0) Present; (1) Vestigial or absent.

***Yubaatar* = 1**

3. (Luo et al. 2002: 20) Anterior-ventral extension of masseteric fossa to mandibular body below m1: (0) Absent; (1) Present and extending to below m1; (2) Present and extending more anteriorly than the p4-m1 junction.

***Yubaatar* = 1**

4. Coronoid or coronoid scar on mandible: (0) Present; (1) Absent.

***Yubaatar* = 1**

5. (Luo33, KJ-H57 states are switched, distribution same) Angle of the coronoid anterior margin to the molar alveolar line on the mandibular body: (0) > 45 degrees (Luo33 states 1-2; Note: more steep in plesiomorphic taxa than in cimolodontans); (1) Low < 45 degrees (Luo33 states 2-3).

***Yubaatar* = 0**

6. (KJ-H 58) Coronoid process orientation in parasaggital plane: (0) Parallel to the rest of the outer wall of the dentary; (1) Flared laterally.

***Yubaatar* = 0**

7. (R67; KJ-H 35) Angle between the lower margin of the dentary and alveolar line of the lower p4 and molars: (0) 11-17 degrees; (1) 18 degrees or above.

***Yubaatar* = 0**

8. (Luo 31) Gracile and elongate dentary peduncle: (0) Present; (1) Absent.

***Yubaatar* = 1**

9. (KJ-H60) Mandibular condyle height to the m1-m2 alveolar line: (0) Opposite or below the level of the molar alveoli; (1) Above the level of the molars.

***Yubaatar* = 1**

10. Mandibular angle: presence vs. absence: (0) Present; (1) Absent.

***Yubaatar* = 1**

Incisor and Canine Features

11. (Luo142) Number of lower incisors: (0) Four or More; (1) One.

    ***Yubaatar* = 1**

12. (Luo166) Procumbency and enlargement of the lower anterior-most incisors: (0) Absent; (1) Present (at least 50% longer than the adjacent incisor).

    ***Yubaatar* = 1**

13. (R2) Lower incisor 1 root posterior extension: (0) Not extending below p3; (1) Extending posteriorly beyond p3-p4 junction.

    ***Yubaatar* = 1**

14. (Luo167) Enlarged diastema in lower incisor-canine region (More developed in older individuals): (0) Present and behind the canine; (1) Present and behind the posterior incisor.

    ***Yubaatar* = 1**

15. (KJ-H21) Lower incisor robustness: (0) Robust; (1) Gracile.

    ***Yubaatar* = 0**

16. (R3; KJ-H 20) Enamel covering of lower incisor of uniform thickness (0); thicker on labial surface than on lingual surface (1); or completely restricted to labial surface of tooth (2).

    ***Yubaatar* = 2**

17. (R18; Luo143) Number of upper incisors (ordered): (0) Four or more; (1) Three; (2) Two.

    ***Yubaatar* = ?**

18. (Y18) Diastema between Upper Incisor 2 and Incisor 3: (0) No large diastema between the second incisor and third upper incisors; (1) I2-I3 diastema.

    ***Yubaatar* = ?**

19. (R19; KJ-H3: states and distribution revised) Upper Incisor 2 (or penultimate incisor) morphology: (0) Peg-like or single cusp; (1) Two-cusped, or more.

    ***Yubaatar* = ?**

20. (R21; Modified from KJ-H4) Upper Incisor 3 (or ultimate upper incisor) morphology: (0) Single cusped or peg-like; (1) 2-cusped; (2) 3-4-cusped.

***Yubaatar* = ?**

21. (R22; KJ-H 13) Placement of posterior upper incisor (I3): (0) On the margin of premaxilla; (1) Medial to the margin-crest of facio-palatal faces of premaxilla; (2) More internal on the palatal part (close to the first premolar).

***Yubaatar* = ?**

22. (R23; KJ-H5-Luo148) Upper canine - presence vs. absence, and size: (0) Present and enlarged; (1) Present and small; (2) Absent.

***Yubaatar* = 2**

23. (R24) Upper canine – number of cusps: (0) Peg-like with single cusp; (1) Two or more cusps.

***Yubaatar* = ?**

24. (Luo150) Lower canine - presence vs. absence: (0) Present; (1) Absent.

***Yubaatar* = 1**

Premolar Features

25. (KJ-H15) Ratio [of the length] between I3 and first maxillary tooth vs. length of upper premolars and molars (ordered): (0) Below 0.09; (1) Between 0.1-0.19; (2) 0.2 or above.

***Yubaatar* = ?**

26. (KJ-H6; Luo152) Number of upper premolars (only applicable to taxa with premolar vs. molar differentiation) (ordered): (0) Five; (1) Four; (2) Three; (3) Two or one.

***Yubaatar* = 1**

27. (R31, R34; KJ-H14) Root(s) of posterior upper premolar(s): (0) Double-rooted; (1) Single-rooted.

***Yubaatar* = 0**

28. (KJ-H7) Labial cuspules on posterior upper premolars: (0) Absent; (1) Present.

***Yubaatar* = 0**

29. (KJ-H8) Upper premolars/upper molars length ratio (re-measured and revised) (ordered): (0) 1.5 or more; (1) 1.5-0.8; (2) 0.8 or less.

***Yubaatar* = 1**

30. (KJ-H9) Last upper premolar: number and length of cusp rows: (0) Two main and equal rows of cusps; (1) Two equal rows of cusps and a continuous row of labial cingular cuspules or cingulum: (2) One main row and a shorter buccal row (anterior or posterior); (3) One main row only on P4.

***Yubaatar* = 2/3**

31. P4 (or penultimate upper premolar) [cusp number] - the main labial cusp row (excluding the labial cingular cuspules) (KJ-H18, modified; herein the multituberculate labial row is considered the same row as the main row of upper premolar of *Morganucodon*) (ordered): (0) 1-4; (1) 5-8; (2) 9-10.

***Yubaatar* = 0**

32. (KJ-H19) Width ratio of ultimate upper premolar vs M1 (ordered): (0) More than 0.9; (1) 0.9-.07; (2) 0.69-0.4; (3) 0.39-0.2.

***Yubaatar* = 2/3**

33. (R5; KJ-H22, distribution revised) Lower p1: (0) Present; (1) Absent.

***Yubaatar* = 1**

34. (R6; KJ-H23) Lower p2: (0) Present; (1) Absent.

***Yubaatar* = 1**

35. (R7; KJ-H24, modified) Lower p3 (or penultimate premolar) presence vs. absence: (0) Present; (1) Absent.

***Yubaatar* = 1**

36. (Luo160) p3 (or penultimate lower premolar) laterally compressed to be bladelike: (0) No (including taxa with peg-like p3); (1) Yes.

***Yubaatar* =?**

37. (R8; KJ-H24, modified) Lower p3 (or penultimate premolar) cusp/serration count: (0) Present, with 3-5 cusps; (1) Present, with 1-2 cusps.

***Yubaatar* =?**

38. (KJ-H25) Shape of p3: (0) Single row of cusps; (1) Blade-like rectangular; (2) Blade-like triangular; (3) Peg-like.

***Yubaatar* =?**

39. (KJ-H 26) Labial basal cuspules on p3 (applicable to bladed lower premolars): (0) Present; (1) Absent.

***Yubaatar* =?**

40. Contact of penultimate and ultimate lower premolars: (0) Juxtaposition; (1) Staged: p4 overhanging p3.

***Yubaatar* =?**

41. (Luo160) Lower p4 (or ultimate lower premolar) laterally compressed to be bladelike: (0) No; (1) Yes.

***Yubaatar* = 0**

42. (R11; KJ-H27) Lower p4 (or ultimate premolar) profile in lateral view: (0) Tricuspate; (1) Rectangular; (2) Arcuate; (3) Triangular.

***Yubaatar* = 2**

43. (R9; KJ-H28) Blade-like p4 serration count (character state modified) (ordered): (0) 7 or less; (1) 8-10; (2) More than 10.

***Yubaatar* = 1**

44. (Y44) Lower p4 anterior root - Exoedaenodont at crown-root junction (Krause (1977), synonymous as the “triangular premolar lobe” of Kielan-Jaworowska et al. (2004)): (0) Absent; (1) Present.

***Yubaatar* = 0**

45. (KJ-H34) Dorsal margin of p4 to m1: (0) On the level of molars; (1) Protruding dorsally over molars.

***Yubaatar* = 0**

46. (KJ-H29) p3/p4 maximum length ratio (ordered): (0) Above 0.7; (1) 0.7 – 0.4; (2) 0.39-0.11; (3) 0.10 or less.

***Yubaatar* = ?**

47. (KJ-H30) Labial basal cuspules on p4 (ordered): (0) Absent; (1) Single cusp or a simple cingulid; (2) Several-single row; (3) Several-double rows.

***Yubaatar* = 1**

48. (W14; KJ-H31) Ratio of p4:m1 length (ordered): (0) Less than 0.99; (1) 1-1.49; (2) 1.5-1.99; (3) 2 or greater.

***Yubaatar* = 0**

Molar Features

49. (Luo154) Number of lower molars or molariform postcanines (ordered): (0) Three or more; (1) Two molars.

***Yubaatar* = 1**

50. (Luo155) Number of upper molars or molariform postcanines: (0) Three or more; (1) Two.

***Yubaatar* = 1**

51. (Luo156) Total number of upper postcanine loci (ordered): (0) More than 8 (including the loci plus the alveoli of shed anterior postcanines): (1) Seven loci; (2) Six; (3) Five or fewer.

***Yubaatar* = 2**

52. (Luo157) Number of lower postcanine loci (ordered): (0) Eight or more; (1) Six; (2) Five or less.

***Yubaatar* = 2**

53. (Luo168 originally from Butler, 2000) U-shaped transverse ridge(s) between b1-l1, or b2-l2 cusps in lower molars: (0) Absent; (1) Present; (2) Sharp crest closes the narrow central valley.

***Yubaatar* = 0**

54. (Luo169 originally from Butler, 2000) Lower molar 1 longitudinal cusp row(s) (the buccal row in case of multi-rows) – relative cusp height: (0) Second mesial cusp (b2 of Butler 2000) highest; (1) All cusps are of equal height.

***Yubaatar* = 1**

55. M1 lingual row cusp count (applicable only to teeth with multi-row of multicusps) (Ordered): (0) 4 or fewer; (1) 5; (2) 7-9 or more.

Note: This character was not used by Mao et al. [in press]; instead, they used the one from Rougier et al. (1997) and Kielan-Jarworowska and Hurum (2001): (R38; KJ-H10) Ml cusp formula 2-3:2-4:0 (0), 4-5:4-5:0-1 (1), 5-7:5-8:2-5 (2), or 5-11:7-10:6-11 (3).

***Yubaatar* = 2**

56. (Luo170 originally from Butler, 2000) Penultimate upper molar 1 (M1) of multirowed upper molar (or postcanines) - cusp height ratio in the lingual row: (0) Distal cusp or second distal cusp highest, with a gradient of anteriorly decreasing height; (1) Cusps in same row of equal height.

***Yubaatar* = 1**

57. (KJ-H 12) M1 posterolingual wing (applicable to molars with multi-rows of multiple cusps): (0) Absent; (1) Present.

***Yubaatar* = 1**

58. (W18, KJ-H11) M1 posterolingual wing length to M1 length (applicable to M1with multi-rows) (ordered): (0) Present and below 0.2; (1) Present and between 0.2 and 0.5; (2) Present and more than 0.5.

***Yubaatar* = 2**

59. (KJ-H12) M1 posterolingual wing morphology (applicable only to M1s with posterolingual wing) (ordered): (0) Wing smooth; (1) Wing is crested; (2) Wing cuspate with 3-4 cuspules; (3) Wing cuspate with 5 or more cuspules.

***Yubaatar* = 3**

60. (Luo171) Off-set alignment of main cusp-row(s) of the first and second upper molars: (0)

Absent; (1) Present.

***Yubaatar* = 1**

61. (C2) Upper M2 (or ultimate upper molar) middle row cusp count (ordered): (0) Two cusps; (1) Three cusps; (2) Four cusps or more (modified for the reason that some specimens of *Taeniolabis* have more than 4 cusps in the middle row).

***Yubaatar* = 1**

62. (C9-modified) Anterior end of middle valley of Upper M2 (or ultimate upper molar): (0) Middle valley absent; (1) Present as a uniform groove along most length but anterior end closed by a crescentic rim; (2) Uniform valley posteriorly (anteriorly) open.

***Yubaatar* = 2**

63. (Y63) Middle valley of Upper M1 (or penultimate molar): (0) Middle valley absent; (1) Valley present along the tooth length but its posterior end closed by a rim or rim with tiny cuspules; (2) Valley present along most of the tooth length but posterior end closed by a single enlarged cusp in mid position; (3) Uniform valley posteriorly open; (4) Valley indistinctive owing to inflation of tooth cusps.

***Yubaatar* = 3**

64. (C12-modified) Curvature of lingual cusp row of M2 (applicable to multi-rowed M2): (0) Straight; (1) Curved posterolabially.

***Yubaatar* = 0**

65. (Y65) M2 middle valley posterior end (applicable to multi-rowed M2): (0) Valley posteriorly open; (1) Valley closed by a posterior cusp; (2) Valley absent due to inflation of tooth cusps.

***Yubaatar* = 0**

66. M2 anterobuccal ridge (sensu Butler and Hooker, 2005 = crista anterobuccalis of Hahn and Hahn, 1998a) (This was not listed in Yuan et al. [2013], but we think it should be Hahn and Hahn [1998a, b].) (State 2 adopted from Cifelli et al., 2013 character 8): (0) Absent; (1) Present; (2) Present and expanded to mesial margin of main labial cusp row.

***Yubaatar* = 1**

67. (KJ-H36) m1 main lingual row cusp count (distribution revised): (0) 4 or fewer; (1) 5; (2) 6 or higher.

***Yubaatar* = 2**

68. Lower m1 middle valley: (0) Middle valley absent; (1) Present and posteriorly open and completely separating distal cusps of lingual vs. labial rows; (2) Middle valley present, but rimmed posteriorly and projected distally.

***Yubaatar* = 1**

69. (KJ-H33, modified) m2 middle longitudinal valley: (0) Middle valley absent; (1) Present and completely separating two rows of cusps; (2) Middle valley present, incomplete and rimmed anteriorly and/or posteriorly.

***Yubaatar* = 1**

70. Coalescence of m2 labial row cusps (applicable only to teeth with multi-rows of multiple cusps): (0) Absent; (1) Present.

***Yubaatar* = 0**

71. (R17) Complete rim of m2 basin (applicable only to teeth with multi-rows of multiple cusps): (0) Absent; (1) Present.

***Yubaatar* = 0**

72. (KJ-H 37, modified to restrict to lingual row, switched states 1 vs. 2) m2 lingual row cusp count (ordered): (0) One trenchant anterior cusp; (1) 2-3; (2) 4-5.

***Yubaatar* = 2**

73. (Luo 171) Ultimate lower molar (or m2) with multi-rows – ratio of row length: (0) Labial cusp row about equal as lingual cusp row; (1) Labial row is longer (by at least half-cusp length) than lingual row.

***Yubaatar* = 0**

74. (KJ-H38) Cusp shape on lower molars: (0) Conical; (1) Crescentic; (2) inflated.

***Yubaatar* = 0**

75. (Hahn and Hahn, 1998) (This was not listed in Yuan et al. [2013], but we think it should be Hahn and Hahn, [1998a, b].) Enlarged trenchant second cusp of lingual row enclosed

into the basin on lower m1 (applicable only to molars with multi-rows of multiple cusps) (0) Absent; (1) b2 cusp enlarged; (2) b2 cusp enlarged and encircled into the basin.

***Yubaatar* = 0**

76. (KJ-H 39; C1) Molar enamel surface: (0) Not ornamented; (1) Covered with grooves, pits and ridges.

***Yubaatar* = 0**

77. (KJ-H1) Enamel microstructure: (0) prismless (preprismatic of Simmons 1993): (1) Gigantoprismatic; (2) Small prismatic.

***Yubaatar* = ?**

78. Differentiation of premolars vs. molars: (0) Absent; (1) Present.

***Yubaatar* = 1**

79. Diphyodont dental replacement: (0) Absent; (1) Present.

***Yubaatar* = 1**

Cranial Features (from R 1997 and KJ-H 2001)

80. (R43; KJ-H16) Infraorbital foramen: (0) Double; (1) Single.

***Yubaatar* = 1**

81. (KJ-H17) Palatal vacuities: (0) Absent; (1) Present and single; (2) Present and double.

***Yubaatar* = 1 / 2**

82. (KJ-H40) Sharp ridge between the palate and lateral walls of premaxilla (0) Absent; (1) Present.

***Yubaatar* = 1**

83. Curvature of anterior zygomatic root (best viewed in dorsal view) (R48; Modified from KJ-H41): (0) Zygomatic root aligned with (or lightly incurved into) facial part of rostrum; (1) Zygomatic root is more transverse and deeply incurved with facial part of rostrum.

***Yubaatar* = 1**

84. (KJ-H42) Number of pairs of vascular foramina on nasal: (0) Absent; (1) One; (2) Two;

(3) Three or more.

***Yubaatar* = 3**

85. (R44; KJ-H43) Posterior-most infraorbital foramen positions (ordered): (0) Dorsal to P3 or P4, or more posterior: (1) Dorsal to P2; (2) Dorsal to P1.

***Yubaatar* = 0**

86. (R49; KJ-H44) Base of zygomatic arch as marked by posterior edge: (0) Dorsal to P4 or more anterior; (1) Dorsal or posterior to P5/M1 or P4/M1 embrasure, or further posterior.

***Yubaatar* = 1**

87. (R47) Bony roof over anterior orbital space: (0) Absent; (1) Present.

***Yubaatar* = 0**

88. (R50, KJ-H45) Postorbital process: (0) Absent: (1) Present and short; (2) Present and long.

***Yubaatar* = 1**

89. (R52, KJ-H46) Snout length: (0) < 49 percent of total skull length; (1) > 50 percent of skull length.

***Yubaatar* = ?**

90. (KJ-H47, modified) Frontal/nasal suture pattern: (0) With subtransverse anterior margins or zigzag line; (1) Pointed anteriorly and not deeply inserted between the nasals; (2) Frontal deeply inserted between the nasals.

***Yubaatar* = 2**

91. (R53, KJ-H48) Frontal-parietal suture: (0) Roughly V-shaped parietal lappet into frontal; (1) U-shaped.

***Yubaatar* = 0**

92. (R54; KJ-H 49) Contacts between nasals and parietals: (0) Absent; (1) Present.

***Yubaatar* = 0**

93. (KJ-H50) Facial exposure of lacrimal: (0) Very small and arcuate; (1) Large, roughly rectangular.

***Yubaatar* = 1**

94. (R56; KJ-H51) Thickening in palatal process of premaxilla: (0) Absent; (1) Present.

***Yubaatar* = 0**

95. (KJ-H52) Incisive foramen positioned: (0) Within premaxilla; (1) Limited posteriorly by maxilla.

***Yubaatar* =?**

96. (R61; KJ-H53) Foramen ovale inferium placement: (0) Medial to foramen masticatorium: (1) Posterior to foramen masticatorium:

***Yubaatar* =?**

97. (R62; KJ-H54) Jugular fossa: (0) Small and shallow: (1) Large and deep.

***Yubaatar* = 1**

98. (KJ-H55) Anterior part of promontorium (sensu Hurum et al. 1996): (0) Oval; (1) Irregular within curvatures on both sides.

***Yubaatar* = 1**

99. (KJ-H56) Glenoid fossa length-width ratio (length as measured as maximum dimension from anterolateral to posteromedial end of glenoid for multituberculates) (re-measured and distribution modified): (0) 1.5 or more; (1) Below 1.49.

***Yubaatar* = 1**

100. (KJ-H59) Post-temporal fossa: (0) Large: (1) Reduced to a small foramen.

***Yubaatar* = 1**

101. (KJ-H61) Width of the snout: skull length ratio (change of character state partition: (0) Above 0.4; (1) Below 0.39.

***Yubaatar* =?**

102. (KJ-H62) Skull width/length ratio: (0) 0.79 and below; (1) Above 0.8.

***Yubaatar* =?**

IV. Phylogenetic Analyses with all characters unordered

```
Consensus of 18 trees

Source of trees from which consensus(es) calculated...
Heuristic search settings:
  Optimality criterion = parsimony
    Character-status summary:
      Of 102 total characters:
        All characters are of type 'unord'
        All characters have equal weight
        2 characters are parsimony-uninformative
        Number of parsimony-informative characters = 100
    Gaps are treated as "missing"
    Multistate taxa interpreted as polymorphism
  Starting tree(s) obtained via stepwise addition
  Addition sequence: random
    Number of replicates = 1000
    Starting seed = 1310845992
  Number of trees held at each step during stepwise addition = 10
  Branch-swapping algorithm: tree-bisection-reconnection (TBR)
  Steepest descent option not in effect
  Initial 'MaxTrees' setting = 10000 (will be auto-increased by 100)
  Branches collapsed (creating polytomies) if maximum branch length is zero
  'MulTrees' option not in effect; only 1 tree will be saved per replicate
  Topological constraints not enforced
  Trees are unrooted

Heuristic search completed
   Total number of rearrangements tried = 48382575
   Score of best tree(s) found = 355
   Number of trees retained = 18
   Time used = 51.61 sec
```

Tree description:

```
  Unrooted tree(s) rooted using outgroup method
  Optimality criterion = parsimony
    Character-status summary:
      Of 102 total characters:
        All characters are of type 'unord'
```

All characters have equal weight
2 characters are parsimony-uninformative
Number of parsimony-informative characters = 100

Gaps are treated as "missing"
Multistate taxa interpreted as uncertainty
Character-state optimization: Accelerated transformation (ACCTRAN)

Figure 1 - Strict Consensus Tree (of 18 EMTs) with all characters unordered

Tree length = 349
Consistency index (CI) = 0.4556
Homoplasy index (HI) = 0.5444
CI excluding uninformative characters = 0.4524
HI excluding uninformative characters = 0.5476
Retention index (RI) = 0.7552
Rescaled consistency index (RC) = 0.3440

```
/---------------------------------------------------------- Sinoconodon
|
+---------------------------------------------------------- Morganucodon
|
|   /------------------------------------------------------ Thomasia
|   |
|   +------------------------------------------------------ Haramiyavia
|   |
75  |                                      /-------- Meketichoffatia
|   |                                      |
|   |                                      |   /---- Henkelodon
|   |                                      +--45
|   |                                      |   \---- Kuehneodon
|   |                                      |
|   |  /----------------------------------------46-------- Rugosodon
\--74  |                                   |
    |  |                                   +-------- Paulchoffatia
    |  |                                   |
    |  |                                   +-------- Meketibolodon
    |  |                                   |
    |  |                                   \-------- Guimarotodon
    |  |
    |  |                                      /---- Ctenacodon
    |  |                                      |
    |  |                                   /--47---- Glirodon
    |  |                                   |  |
   \--73                                /--48  \---- Zofiabaatar
       |                                |  |
       |  /---------------------------------49  \-------- Plagiaulax
       |  |                             |
       |  |                             \------------ Bolodon
       |  |
       |  |                                   /---- Sinobaatar
       |  |  /--------------------------------50
       |  |  |                                \---- Eobaatar
       |  |  |
       |  |  |   /--------------------------------------- Arginbaatar
```

```
\--72   |   |
    |   |   |                               /---- Cimexomys
    |   |   |          /--------------------51
    |   |   |          |                    \---- Meniscoessus
    |   |   |          |
    |   |   |          |   /------------------------ Boffius
    |   |   |          |   |
    |   |   |          |   +------------------------ Buginbaatar
    \--71   |          |   |
        |   |          |   |                /---- Pentacosmodon
        |   |          |   +-----------------52
        |   |     /---64   |                   \---- Microcosmodon
        |   |     |    |   |
        |   |     |    |   |                /---- Catopsbaatar
        |   |     |    |   |            /--53
        |   |     |    |   |            |   \---- Kryptobaatar
        |   |     |    |   |         /--54
        |   |     |    |   |         |   \-------- Nemegtbaatar
        |   |     |    |   +---------56
        \--70     |    \--63         |      /---- Kamptobaatar
            |     |        |         \------55
            |     |        |                \---- Chulsanbaatar
            |     |        |
            |     |        +------------------------ Eucosmodon
            |     |        |
            |     |        +------------------------ Stygimys
            |     |        |
            |     |        |  /-------------------- Kogaionon
            |     |        |  |
            |  /--68       |  |             /---- Taeniolabis
            |  |  |        |  |      /------57
            |  |  |        |  |      |      \---- Catopsalis
            |  |  |        \--62      |
            |  |  |           |  /--60      /---- Lambdopsalis
            |  |  |           |  |   |  /--58
            |  |  |           |  |   |  |   \---- Sphenopsalis
            |  |  |           \--61  \--59
            |  |  |              |      \-------- Prionessus
            |  |  |              |
            \--69 |              \---------------- Yubaatar
               |  |
               |  |                         /---- Ectypodus
               |  |                     /--65
               |  |                     |   \---- Ptilodus
               |  |                  /--66
               |  |                  |   \-------- Neoliotomus
               |  \------------------67
               |                     \------------ Mesodma
               |
               \-------------------------------------- Cimolodon
```

Apomorphy lists:

Branch	Character	Steps	CI	Change
node_75 --> Sinoconodon	78	1	1.000	1 ==> 0
	79	1	1.000	1 ==> 0
node_75 --> Morganucodon	84	1	0.667	1 ==> 0
node_75 --> node_74	4	1	0.500	0 --> 1

	5	1	0.125	0 --> 1
	12	1	1.000	0 ==> 1
	22	1	0.667	0 ==> 1
	42	1	0.600	0 --> 1
	53	1	0.667	0 ==> 1
	62	1	0.667	0 ==> 1
	63	1	1.000	0 ==> 3
	66	1	0.667	0 --> 1
	68	1	0.667	0 ==> 1
	69	1	0.667	0 ==> 2
	77	1	0.500	0 ==> 1
	89	1	0.500	1 --> 0
	93	1	0.333	0 --> 1
	95	1	0.500	0 --> 1
	101	1	0.500	0 --> 1
node_74 --> Thomasia	26	1	0.500	0 ==> 3
	32	1	0.429	0 ==> 1
	66	1	0.667	1 --> 0
node_74 --> Haramiyavia	18	1	0.333	0 ==> 1
	42	1	0.600	1 --> 0
node_74 --> node_73	1	1	1.000	0 ==> 1
	2	1	1.000	0 ==> 1
	3	1	0.400	0 ==> 1
	8	1	1.000	0 ==> 1
	10	1	1.000	0 ==> 1
	11	1	1.000	0 ==> 1
	14	1	1.000	0 ==> 1
	17	1	1.000	0 ==> 1
	19	1	0.167	0 ==> 1
	20	1	0.667	0 ==> 1
	24	1	1.000	0 ==> 1
	28	1	0.500	0 --> 1
	29	1	0.286	2 ==> 0
	36	1	0.500	0 ==> 1
	38	1	0.600	0 --> 1
	41	1	0.500	0 ==> 1
	45	1	0.500	0 ==> 1
	47	1	0.333	0 --> 2
	48	1	0.231	0 ==> 1
	49	1	1.000	0 ==> 1
	50	1	1.000	0 ==> 1
	51	1	0.600	0 ==> 1

	52	1	1.000	0	==>	1
	56	1	0.500	0	==>	1
	60	1	1.000	0	==>	1
	61	1	0.200	1	-->	0
	72	1	0.222	2	-->	0
	76	1	0.250	0	==>	1
node_73 --> node_46	23	1	1.000	0	==>	1
	46	1	1.000	1	==>	0
	63	1	1.000	3	==>	1
	64	1	0.500	0	==>	1
	65	1	1.000	0	==>	1
	66	1	0.667	1	-->	2
	68	1	0.667	1	==>	2
	70	1	0.500	0	==>	1
	71	1	1.000	0	==>	1
	75	1	1.000	0	==>	2
	94	1	0.333	0	-->	1
node_46 --> Meketich.	56	1	0.500	1	==>	0
node_46 --> node_45	4	1	0.500	1	-->	0
	13	1	0.333	0	-->	1
	20	1	0.667	1	==>	2
	63	1	1.000	1	==>	2
	72	1	0.222	0	-->	1
node_45 --> Henkelodon	22	1	0.667	1	==>	2
node_45 --> Kuehneodon	26	1	0.500	0	==>	1
	51	1	0.600	1	==>	2
node_46 --> Rugosodon	13	1	0.333	0	==>	1
	75	1	1.000	2	==>	1
node_46 --> Meketib.	47	1	0.333	2	-->	3
node_46 --> Guimarot.	47	1	0.333	2	-->	3
node_73 --> node_72	5	1	0.125	1	-->	0
	7	1	0.143	0	-->	1
	13	1	0.333	0	==>	1
	38	1	0.600	1	-->	2
	39	1	1.000	0	-->	1
	53	1	0.667	1	==>	0
	54	1	1.000	0	==>	1
	57	1	0.500	0	==>	1
	62	1	0.667	1	==>	2
	69	1	0.667	2	==>	1
	72	1	0.222	0	-->	1
	73	1	0.333	0	==>	1

	82	1	0.667	0 ==> 1
	99	1	0.500	0 --> 1
node_72 --> node_49	9	1	0.167	0 --> 1
	30	1	0.750	0 ==> 1
	77	1	0.500	1 ==> 0
	85	1	0.400	0 ==> 1
node_49 --> node_48	76	1	0.250	1 ==> 0
node_48 --> node_47	38	1	0.600	2 ==> 1
node_47 --> Ctenacodon	55	1	0.300	0 ==> 1
node_47 --> Glirodon	16	1	0.250	0 ==> 1
	57	1	0.500	1 ==> 0
node_47 --> Zofiabaat.	48	1	0.231	1 ==> 3
node_48 --> Plagiaulax	33	1	0.500	0 ==> 1
	43	1	0.222	0 ==> 1
node_49 --> Bolodon	5	1	0.125	0 --> 1
node_72 --> node_71	17	1	1.000	1 ==> 2
	18	1	0.333	0 ==> 1
	21	1	0.667	0 --> 1
	22	1	0.667	1 ==> 2
	28	1	0.500	1 --> 0
	32	1	0.429	0 ==> 1
	33	1	0.500	0 ==> 1
	37	1	0.500	0 --> 1
	42	1	0.600	1 ==> 2
	43	1	0.222	0 --> 1
	44	1	0.250	0 ==> 1
	46	1	1.000	1 ==> 2
	47	1	0.333	2 --> 0
	52	1	1.000	1 ==> 2
	61	1	0.200	0 --> 1
	80	1	0.333	0 --> 1
	86	1	0.200	0 --> 1
node_71 --> node_50	19	1	0.167	1 --> 0
	47	1	0.333	0 --> 1
	48	1	0.231	1 --> 2
	58	1	0.222	0 ==> 1
	70	1	0.500	0 ==> 1
node_50 --> Sinobaatar	30	1	0.750	0 ==> 2
	55	1	0.300	0 ==> 1
	77	1	0.500	1 ==> 0
node_50 --> Eobaatar	16	1	0.250	0 ==> 2
	37	1	0.500	1 --> 0

node_71 --> node_70	3	1	0.400	1 ==> 2
	7	1	0.143	1 --> 0
	36	1	0.500	1 ==> 0
	40	1	1.000	0 ==> 1
	43	1	0.222	1 --> 2
node_70 --> Arginbaat.	48	1	0.231	1 ==> 3
	61	1	0.200	1 --> 0
	76	1	0.250	1 ==> 0
	80	1	0.333	1 --> 0
node_70 --> node_69	26	1	0.500	0 ==> 1
	29	1	0.286	0 ==> 1
	30	1	0.750	0 ==> 2
	31	1	0.286	0 ==> 1
	34	1	0.500	0 ==> 1
	38	1	0.600	2 ==> 3
	51	1	0.600	1 ==> 2
	55	1	0.300	0 ==> 2
	58	1	0.222	0 ==> 2
	59	1	0.429	0 --> 2
	67	1	0.286	0 ==> 2
node_69 --> node_68	5	1	0.125	0 ==> 1
	46	1	1.000	2 ==> 3
	59	1	0.429	2 --> 3
node_68 --> node_64	43	1	0.222	2 --> 1
	45	1	0.500	1 ==> 0
	48	1	0.231	1 --> 0
	97	1	1.000	0 --> 1
node_64 --> node_51	25	1	0.400	0 --> 2
	32	1	0.429	1 ==> 2
	67	1	0.286	2 ==> 1
	81	1	0.400	0 --> 2
node_51 --> Cimexomys	34	1	0.500	1 ==> 0
	48	1	0.231	0 --> 2
	59	1	0.429	3 ==> 2
node_51 --> Meniscoes.	29	1	0.286	1 ==> 2
	31	1	0.286	1 ==> 0
	61	1	0.200	1 ==> 2
	72	1	0.222	1 ==> 2
	74	1	0.500	0 ==> 1
	80	1	0.333	1 ==> 0
node_64 --> node_63	16	1	0.250	0 ==> 2
	35	1	0.333	0 ==> 1

	76	1	0.250	1 ==> 0
	84	1	0.667	1 ==> 3
	86	1	0.200	1 --> 0
	94	1	0.333	0 --> 1
node_63 --> Boffius	16	1	0.250	2 ==> 1
	55	1	0.300	2 ==> 3
node_63 --> Buginbaat.	3	1	0.400	2 ==> 1
	16	1	0.250	2 ==> 0
	32	1	0.429	1 ==> 3
	43	1	0.222	1 ==> 0
	44	1	0.250	1 ==> 0
	55	1	0.300	2 ==> 3
	58	1	0.222	2 ==> 1
	61	1	0.200	1 ==> 2
node_63 --> node_52	5	1	0.125	1 ==> 0
	9	1	0.167	0 ==> 1
	16	1	0.250	2 ==> 1
	32	1	0.429	1 --> 2
	38	1	0.600	3 --> 2
	43	1	0.222	1 ==> 0
	47	1	0.333	0 ==> 1
	58	1	0.222	2 ==> 1
	83	1	0.500	0 --> 1
node_52 --> Pentacos.	7	1	0.143	0 ==> 1
	31	1	0.286	1 ==> 0
	67	1	0.286	2 ==> 0
	82	1	0.667	1 ==> 2
node_52 --> Microcos.	35	1	0.333	1 ==> 0
	48	1	0.231	0 --> 2
node_63 --> node_56	19	1	0.167	1 ==> 0
	21	1	0.667	1 ==> 2
	32	1	0.429	1 ==> 2
	35	1	0.333	1 ==> 0
	48	1	0.231	0 --> 1
	55	1	0.300	2 --> 1
	59	1	0.429	3 --> 1
	67	1	0.286	2 ==> 0
	82	1	0.667	1 ==> 2
	85	1	0.400	0 --> 1
	87	1	1.000	0 ==> 1
	90	1	1.000	0 ==> 1
	91	1	1.000	0 ==> 1

	99	1	0.500	1 ==> 0
node_56 --> node_54	59	1	0.429	1 --> 2
	61	1	0.200	1 ==> 2
node_54 --> node_53	83	1	0.500	0 ==> 2
	84	1	0.667	3 ==> 2
	88	1	0.500	0 ==> 1
	89	1	0.500	0 ==> 1
	98	1	0.500	0 ==> 1
	101	1	0.500	1 ==> 0
	102	1	0.500	0 ==> 1
node_53 --> Catopsbaat.	5	1	0.125	1 ==> 0
	7	1	0.143	0 ==> 1
	9	1	0.167	0 ==> 1
	26	1	0.500	1 ==> 2
	29	1	0.286	1 ==> 2
	42	1	0.600	2 ==> 1
	43	1	0.222	1 ==> 0
	44	1	0.250	1 ==> 0
	48	1	0.231	1 ==> 2
	51	1	0.600	2 ==> 3
	55	1	0.300	1 --> 2
	59	1	0.429	2 --> 3
	61	1	0.200	2 ==> 0
	85	1	0.400	1 --> 0
	86	1	0.200	0 ==> 1
	95	1	0.500	1 ==> 0
node_53 --> Kryptob.	58	1	0.222	2 ==> 0
node_54 --> Nemegtb.	25	1	0.400	0 ==> 1
	81	1	0.400	0 ==> 2
	96	1	0.333	1 ==> 0
node_56 --> node_55	58	1	0.222	2 ==> 0
	85	1	0.400	1 --> 2
node_55 --> Kamptob.	6	1	0.500	0 ==> 1
	9	1	0.167	0 ==> 1
	16	1	0.250	2 ==> 1
	96	1	0.333	1 ==> 0
node_55 --> Chulsanb.	7	1	0.143	0 ==> 1
	84	1	0.667	3 ==> 2
node_63 --> Eucosm.	7	1	0.143	0 ==> 1
	26	1	0.500	1 ==> 2
	43	1	0.222	1 --> 2
	48	1	0.231	0 --> 1

	67	1	0.286	2 ==> 1
	72	1	0.222	1 ==> 2
node_63 --> Stygimys	3	1	0.400	2 ==> 1
	25	1	0.400	0 ==> 1
	31	1	0.286	1 ==> 2
	43	1	0.222	1 --> 2
	47	1	0.333	0 ==> 1
	48	1	0.231	0 --> 1
	58	1	0.222	2 ==> 1
	81	1	0.400	0 ==> 2
	85	1	0.400	0 ==> 2
node_63 --> node_62	5	1	0.125	1 --> 0
	9	1	0.167	0 --> 1
	19	1	0.167	1 --> 0
	25	1	0.400	0 --> 1
	30	1	0.750	2 ==> 3
	31	1	0.286	1 ==> 0
	41	1	0.500	1 --> 0
	44	1	0.250	1 --> 0
	47	1	0.333	0 --> 1
	73	1	0.333	1 --> 0
	83	1	0.500	0 ==> 1
	94	1	0.333	1 --> 0
	98	1	0.500	0 --> 1
	100	1	1.000	0 --> 1
node_62 --> Kogaionon	29	1	0.286	1 ==> 0
	55	1	0.300	2 ==> 1
	59	1	0.429	3 ==> 2
	61	1	0.200	1 ==> 0
node_62 --> node_61	18	1	0.333	1 --> 0
	20	1	0.667	1 --> 0
	21	1	0.667	1 --> 0
	25	1	0.400	1 --> 2
	32	1	0.429	1 ==> 3
	86	1	0.200	0 ==> 1
	88	1	0.500	0 ==> 1
	90	1	1.000	0 ==> 2
	102	1	0.500	0 --> 1
node_61 --> node_60	26	1	0.500	1 ==> 3
	27	1	1.000	0 ==> 1
	29	1	0.286	1 ==> 2
	42	1	0.600	2 ==> 3

	51	1	0.600	2 ==> 3
	55	1	0.300	2 ==> 3
	67	1	0.286	2 --> 0
	92	1	1.000	0 ==> 1
	93	1	0.333	1 ==> 0
node_60 --> node_57	19	1	0.167	0 --> 1
	63	1	1.000	3 ==> 4
	65	1	1.000	0 ==> 2
	74	1	0.500	0 ==> 2
node_57 --> Taeniolab.	67	1	0.286	0 --> 2
	72	1	0.222	1 ==> 2
node_57 --> Catopsalis	31	1	0.286	0 ==> 1
node_60 --> node_59	6	1	0.500	0 --> 1
	7	1	0.143	0 --> 1
	47	1	0.333	1 --> 0
	61	1	0.200	1 ==> 0
	62	1	0.667	2 --> 1
node_59 --> node_58	53	1	0.667	0 ==> 2
	68	1	0.667	1 ==> 2
	69	1	0.667	1 ==> 2
	73	1	0.333	0 ==> 1
	74	1	0.500	0 ==> 1
node_61 --> Yubaatar	3	1	0.400	2 ==> 1
	72	1	0.222	1 ==> 2
	81	1	0.400	0 ==> {12}
	84	1	0.667	3 ==> 4
node_68 --> node_67	15	1	0.500	0 --> 1
	58	1	0.222	2 --> 1
	77	1	0.500	1 ==> 2
	81	1	0.400	0 --> 1
	84	1	0.667	1 --> 2
	93	1	0.333	1 --> 0
node_67 --> node_66	19	1	0.167	1 ==> 0
	31	1	0.286	1 ==> 2
	48	1	0.231	1 ==> 3
	55	1	0.300	2 --> 3
	72	1	0.222	1 ==> 2
node_66 --> node_65	5	1	0.125	1 ==> 0
	64	1	0.500	0 ==> 1
node_65 --> Ectypodus	9	1	0.167	0 ==> 1
	83	1	0.500	0 ==> 2
node_65 --> Ptilodus	29	1	0.286	1 ==> 0

	48	1	0.231	3 ==> 2
	86	1	0.200	1 --> 0
	96	1	0.333	1 ==> 0
node_66 --> Neoliot.	15	1	0.500	1 --> 0
	16	1	0.250	0 ==> 2
	58	1	0.222	1 --> 2
	74	1	0.500	0 ==> 1
node_69 --> Cimolodon	61	1	0.200	1 --> 2
	72	1	0.222	1 ==> 2

Character diagnostics:

Character	Range	Min steps	Tree steps	Max steps	CI	RI	RC	HI	G-fit
1	1	1	1	3	1.000	1.000	1.000	0.000	1.000
2	1	1	1	3	1.000	1.000	1.000	0.000	1.000
3	2	2	5	17	0.400	0.800	0.320	0.600	0.500
4	1	1	2	3	0.500	0.500	0.250	0.500	0.750
5	1	1	8	17	0.125	0.563	0.070	0.875	0.300
6	1	1	2	2	0.500	0.000	0.000	0.500	0.750
7	1	1	7	11	0.143	0.400	0.057	0.857	0.333
8	1	1	1	3	1.000	1.000	1.000	0.000	1.000
9	1	1	6	12	0.167	0.545	0.091	0.833	0.375
10	1	1	1	3	1.000	1.000	1.000	0.000	1.000
11	1	1	1	4	1.000	1.000	1.000	0.000	1.000
12	1	1	1	2	1.000	1.000	1.000	0.000	1.000
13	1	1	3	6	0.333	0.600	0.200	0.667	0.600
14	1	1	1	3	1.000	1.000	1.000	0.000	1.000
15	1	1	2	3	0.500	0.500	0.250	0.500	0.750
16	2	2	8	19	0.250	0.647	0.162	0.750	0.333
17	2	2	2	9	1.000	1.000	1.000	0.000	1.000
18	1	1	3	9	0.333	0.750	0.250	0.667	0.600
19	1	1	6	12	0.167	0.545	0.091	0.833	0.375
20	2	2	3	10	0.667	0.875	0.583	0.333	0.750
21	2	2	3	12	0.667	0.900	0.600	0.333	0.750
22	2	2	3	9	0.667	0.857	0.571	0.333	0.750
23	1	1	1	3	1.000	1.000	1.000	0.000	1.000
24	1	1	1	3	1.000	1.000	1.000	0.000	1.000
25	2	2	5	8	0.400	0.500	0.200	0.600	0.500
26	3	3	6	19	0.500	0.813	0.406	0.500	0.500
27	1	1	1	4	1.000	1.000	1.000	0.000	1.000

28	1	1	2	6	0.500	0.800	0.400	0.500	0.750
29	2	2	7	21	0.286	0.737	0.211	0.714	0.375
30	3	3	4	14	0.750	0.909	0.682	0.250	0.750
31	2	2	7	16	0.286	0.643	0.184	0.714	0.375
32	3	3	7	23	0.429	0.800	0.343	0.571	0.429
33	1	1	2	12	0.500	0.909	0.455	0.500	0.750
34	1	1	2	17	0.500	0.938	0.469	0.500	0.750
35	1	1	3	10	0.333	0.778	0.259	0.667	0.600
36	1	1	2	11	0.500	0.900	0.450	0.500	0.750
37	1	1	2	14	0.500	0.923	0.462	0.500	0.750
38	3	3	5	18	0.600	0.867	0.520	0.400	0.600
39	1	1	1	4	1.000	1.000	1.000	0.000	1.000
40	1	1	1	15	1.000	1.000	1.000	0.000	1.000
41	1	1	2	10	0.500	0.889	0.444	0.500	0.750
42	3	3	5	20	0.600	0.882	0.529	0.400	0.600
43	2	2	9	18	0.222	0.563	0.125	0.778	0.300
44	1	1	4	18	0.250	0.824	0.206	0.750	0.500
45	1	1	2	16	0.500	0.933	0.467	0.500	0.750
46	3	3	3	16	1.000	1.000	1.000	0.000	1.000
47	3	3	9	17	0.333	0.571	0.190	0.667	0.333
48	3	3	13	22	0.231	0.474	0.109	0.769	0.231
49	1	1	1	3	1.000	1.000	1.000	0.000	1.000
50	1	1	1	3	1.000	1.000	1.000	0.000	1.000
51	3	3	5	18	0.600	0.867	0.520	0.400	0.600
52	2	2	2	12	1.000	1.000	1.000	0.000	1.000
53	2	2	3	8	0.667	0.833	0.556	0.333	0.750
54	1	1	1	8	1.000	1.000	1.000	0.000	1.000
55	3	3	10	24	0.300	0.667	0.200	0.700	0.300
56	1	1	2	5	0.500	0.750	0.375	0.500	0.750
57	1	1	2	7	0.500	0.833	0.417	0.500	0.750
58	2	2	9	15	0.222	0.462	0.103	0.778	0.300
59	3	3	7	12	0.429	0.556	0.238	0.571	0.429
60	1	1	1	4	1.000	1.000	1.000	0.000	1.000
61	2	2	10	16	0.200	0.429	0.086	0.800	0.273
62	2	2	3	10	0.667	0.875	0.583	0.333	0.750
63	4	4	4	8	1.000	1.000	1.000	0.000	1.000
64	1	1	2	4	0.500	0.667	0.333	0.500	0.750
65	2	2	2	4	1.000	1.000	1.000	0.000	1.000
66	2	2	3	5	0.667	0.667	0.444	0.333	0.750
67	2	2	7	13	0.286	0.545	0.156	0.714	0.375
68	2	2	3	9	0.667	0.857	0.571	0.333	0.750
69	2	2	3	10	0.667	0.875	0.583	0.333	0.750

70	1	1	2	6	0.500	0.800	0.400	0.500	0.750
71	1	1	1	4	1.000	1.000	1.000	0.000	1.000
72	2	2	9	14	0.222	0.417	0.093	0.778	0.300
73	1	1	3	9	0.333	0.750	0.250	0.667	0.600
74	2	2	4	6	0.500	0.500	0.250	0.500	0.600
75	2	2	2	5	1.000	1.000	1.000	0.000	1.000
76	1	1	4	17	0.250	0.813	0.203	0.750	0.500
77	2	2	4	12	0.500	0.800	0.400	0.500	0.600
78	1	1	1	1	1.000	0/0	0/0	0.000	1.000
79	1	1	1	1	1.000	0/0	0/0	0.000	1.000
80	1	1	3	11	0.333	0.800	0.267	0.667	0.600
81	2	2	5	6	0.400	0.250	0.100	0.600	0.500
82	2	2	3	9	0.667	0.857	0.571	0.333	0.750
83	2	2	4	8	0.500	0.667	0.333	0.500	0.600
84	4	4	6	10	0.667	0.667	0.444	0.333	0.600
85	2	2	5	7	0.400	0.400	0.160	0.600	0.500
86	1	1	5	9	0.200	0.500	0.100	0.800	0.429
87	1	1	1	5	1.000	1.000	1.000	0.000	1.000
88	1	1	2	5	0.500	0.750	0.375	0.500	0.750
89	1	1	2	4	0.500	0.667	0.333	0.500	0.750
90	2	2	2	9	1.000	1.000	1.000	0.000	1.000
91	1	1	1	5	1.000	1.000	1.000	0.000	1.000
92	1	1	1	2	1.000	1.000	1.000	0.000	1.000
93	1	1	3	5	0.333	0.500	0.167	0.667	0.600
94	1	1	3	7	0.333	0.667	0.222	0.667	0.600
95	1	1	2	3	0.500	0.500	0.250	0.500	0.750
96	1	1	3	3	0.333	0.000	0.000	0.667	0.600
97	1	1	1	5	1.000	1.000	1.000	0.000	1.000
98	1	1	2	5	0.500	0.750	0.375	0.500	0.750
99	1	1	2	6	0.500	0.800	0.400	0.500	0.750
100	1	1	1	3	1.000	1.000	1.000	0.000	1.000
101	1	1	2	4	0.500	0.667	0.333	0.500	0.750
102	1	1	2	4	0.500	0.667	0.333	0.500	0.750

Figure 2 - 50% Majority Rule Consensus Tree with all characters unordered

Tree length = 334

Consistency index (CI) = 0.4760

Homoplasy index (HI) = 0.5240

CI excluding uninformative characters = 0.4729

HI excluding uninformative characters = 0.5271

```
Retention index (RI) = 0.7745
Rescaled consistency index (RC) = 0.3687

/---------------------------------------------------------- Sinoconodon
|
+---------------------------------------------------------- Morganucodon
|
|   /----------------------------------------------------- Thomasia
|   |
84  | /--------------------------------------------------- Haramiyavia
|   | |
|   | |                                    /------- Meketichoffatia
|   | |                                    |
|   | |                                    +------- Paulchoffatia
\--83 |                                 /--46
    | |                                 |  |  /---- Meketibolodon
    | |                                 |  \-45
    | |                               /-47    \---- Guimarotodon
    | |                               | |
    | |                               | \----------- Rugosodon
    \-82 /---------------------------------49
      |  |                            |       /---- Henkelodon
      |  |                            \-------48
      |  |                                    \---- Kuehneodon
      |  |
      |  |                                    /---- Ctenacodon
      |  |                                    |
      |  |                                  /-50---- Glirodon
      |  |                                  | |
      |  |                              /--51 \---- Zofiabaatar
      \--81                             |   |
         |  /--------------------------------52  \------- Plagiaulax
         |  |                           |
         |  |                           \----------- Bolodon
         |  |
         |  |                                 /---- Sinobaatar
         |  |  /-----------------------------------53
         |  |  |                              \---- Eobaatar
         |  |  |
         |  |  |  /------------------------------------- Arginbaatar
         \-80  |  |
            |  |  |                           /---- Cimexomys
            |  |  |       /----------------------54
            |  |  |       |                   \---- Meniscoessus
            |  |  |       |
            |  |  |       |                 /------- Boffius
            |  |  |       |                 |
            |  |  |       |     /------------56  /---- Pentacosmodon
            \--79 |       |     |           \-55
               |  |       |     |             \---- Microcosmodon
               |  |       |     |
               |  |       |     |  /----------------- Kogaionon
               |  |     /-72  /-63 |
               |  |     | |   | |  |           /---- Taeniolabis
               |  |     | |   | |  |     /-----57
               |  |     | |   | |  |     |     \---- Catopsalis
               |  |     | |   | \--62    |
               |  |     | |   |    |  /-60     /---- Lambdopsalis
               |  |     | |   |    |  |  |  /-58
               \--78    | |   |    |  |  |  |  \---- Sphenopsalis
                  |     | |   |    \--61 \--59
                  |     | |   |       |     \------- Prionessus
```

```
 |      |  \--71         |
 |      |     |         \-------------- Yubaatar
 |      |     |
 |      |     |                  /---- Buginbaatar
 |      |     |               /-64
 |      |     |               |  \---- Stygimys
 |      |     |         /-----65
 |  /--76     |         |      \------- Eucosmodon
 |  |   |     |         |
 |  |   |     |         |        /---- Catopsbaatar
 |  |   |     \---------70     /-66
 |  |   |               |      |  \---- Kryptobaatar
 |  |   |               |  /--67
 |  |   |               |  |   \------- Nemegtbaatar
 |  |   |               \-69
 |  |   |                  |      /---- Kamptobaatar
 |  |   |                  \-----68
 \-77   |                         \---- Chulsanbaatar
    |   |
    |   |                        /---- Ectypodus
    |   |                     /-73
    |   |                     |  \---- Ptilodus
    |   |                 /--74
    |   |                 |   \------- Neoliotomus
    |   \-----------------75
    |                     \----------- Mesodma
    |
    \---------------------------------- Cimolodon
```

Apomorphy lists:

Branch	Character	Steps	CI	Change
node_84 --> Sinoconod.	78	1	1.000	1 ==> 0
	79	1	1.000	1 ==> 0
node_84 --> Morganuc.	84	1	0.667	1 ==> 0
node_84 --> node_83	4	1	0.500	0 --> 1
	5	1	0.143	0 --> 1
	12	1	1.000	0 ==> 1
	22	1	0.667	0 --> 1
	42	1	0.600	0 --> 1
	53	1	0.667	0 ==> 1
	62	1	0.667	0 ==> 1
	63	1	1.000	0 ==> 3
	68	1	0.667	0 --> 1
	69	1	0.667	0 ==> 2
	77	1	0.500	0 --> 1
	89	1	0.500	1 --> 0
	93	1	0.333	0 --> 1
	95	1	0.500	0 --> 1
	101	1	0.500	0 --> 1
node_83 --> Thomasia	26	1	0.500	0 ==> 3

	32	1	0.429	0	==>	1
node_83 --> node_82	28	1	0.500	0	-->	1
	29	1	0.286	2	-->	0
	66	1	1.000	0	==>	1
node_82 --> Haramiy.	18	1	0.333	0	==>	1
	42	1	0.600	1	-->	0
node_82 --> node_81	1	1	1.000	0	==>	1
	2	1	1.000	0	==>	1
	3	1	0.500	0	==>	1
	8	1	1.000	0	==>	1
	10	1	1.000	0	==>	1
	11	1	1.000	0	==>	1
	13	1	0.500	0	==>	1
	14	1	1.000	0	==>	1
	17	1	1.000	0	==>	1
	19	1	0.167	0	==>	1
	20	1	0.667	0	==>	1
	24	1	1.000	0	==>	1
	36	1	0.500	0	==>	1
	38	1	0.600	0	-->	1
	41	1	0.500	0	==>	1
	45	1	0.500	0	==>	1
	47	1	0.429	0	-->	2
	48	1	0.250	0	==>	1
	49	1	1.000	0	==>	1
	50	1	1.000	0	==>	1
	51	1	0.600	0	==>	1
	52	1	1.000	0	==>	1
	56	1	0.500	0	==>	1
	60	1	1.000	0	==>	1
	61	1	0.200	1	-->	0
	72	1	0.250	2	==>	1
	76	1	0.250	0	==>	1
node_81 --> node_49	23	1	1.000	0	==>	1
	46	1	1.000	1	==>	0
	63	1	1.000	3	-->	1
	64	1	0.500	0	==>	1
	65	1	1.000	0	==>	1
	66	1	1.000	1	==>	2
	68	1	0.667	1	==>	2
	70	1	0.500	0	==>	1
	71	1	1.000	0	==>	1

	75	1	1.000	0 ==> 1
	94	1	0.500	0 --> 1
node_49 --> node_47	72	1	0.250	1 ==> 0
node_47 --> node_46	13	1	0.500	1 ==> 0
	56	1	0.500	1 --> 0
	75	1	1.000	1 --> 2
node_46 --> node_45	47	1	0.429	2 ==> 3
node_49 --> node_48	4	1	0.500	1 --> 0
	20	1	0.667	1 ==> 2
	63	1	1.000	1 --> 2
node_48 --> Henkelodon	22	1	0.667	1 ==> 2
node_48 --> Kuehneodon	26	1	0.500	0 ==> 1
	51	1	0.600	1 ==> 2
node_81 --> node_80	5	1	0.143	1 --> 0
	7	1	0.143	0 --> 1
	38	1	0.600	1 --> 2
	39	1	1.000	0 --> 1
	53	1	0.667	1 ==> 0
	54	1	1.000	0 ==> 1
	57	1	0.500	0 ==> 1
	62	1	0.667	1 ==> 2
	69	1	0.667	2 ==> 1
	73	1	0.333	0 ==> 1
	82	1	0.667	0 ==> 1
	99	1	0.500	0 --> 1
node_80 --> node_52	9	1	0.200	0 --> 1
	30	1	0.750	0 ==> 1
	77	1	0.500	1 ==> 0
	85	1	0.500	0 ==> 1
node_52 --> node_51	76	1	0.250	1 ==> 0
node_51 --> node_50	38	1	0.600	2 ==> 1
node_50 --> Ctenacodon	55	1	0.300	0 ==> 1
node_50 --> Glirodon	16	1	0.286	0 ==> 1
	57	1	0.500	1 ==> 0
node_50 --> Zofiabaat.	48	1	0.250	1 ==> 3
node_51 --> Plagiaulax	33	1	0.500	0 ==> 1
	43	1	0.250	0 ==> 1
node_52 --> Bolodon	5	1	0.143	0 --> 1
node_80 --> node_79	17	1	1.000	1 ==> 2
	18	1	0.333	0 ==> 1
	21	1	0.667	0 --> 1
	22	1	0.667	1 ==> 2

	28	1	0.500	1 --> 0
	32	1	0.429	0 ==> 1
	33	1	0.500	0 ==> 1
	37	1	0.500	0 --> 1
	42	1	0.600	1 ==> 2
	43	1	0.250	0 --> 1
	44	1	0.250	0 ==> 1
	46	1	1.000	1 ==> 2
	47	1	0.429	2 --> 0
	52	1	1.000	1 ==> 2
	61	1	0.200	0 --> 1
	80	1	0.333	0 --> 1
	86	1	0.200	0 --> 1
node_79 --> node_53	19	1	0.167	1 --> 0
	47	1	0.429	0 --> 1
	48	1	0.250	1 --> 2
	58	1	0.250	0 ==> 1
	70	1	0.500	0 ==> 1
node_53 --> Sinobaatar	30	1	0.750	0 ==> 2
	55	1	0.300	0 ==> 1
	77	1	0.500	1 ==> 0
node_53 --> Eobaatar	16	1	0.286	0 ==> 2
	37	1	0.500	1 --> 0
node_79 --> node_78	3	1	0.500	1 ==> 2
	7	1	0.143	1 --> 0
	36	1	0.500	1 ==> 0
	40	1	1.000	0 ==> 1
	43	1	0.250	1 --> 2
node_78 --> Arginbaat.	48	1	0.250	1 ==> 3
	61	1	0.200	1 --> 0
	76	1	0.250	1 ==> 0
	80	1	0.333	1 --> 0
node_78 --> node_77	26	1	0.500	0 ==> 1
	29	1	0.286	0 ==> 1
	30	1	0.750	0 ==> 2
	31	1	0.286	0 ==> 1
	34	1	0.500	0 ==> 1
	38	1	0.600	2 ==> 3
	51	1	0.600	1 ==> 2
	55	1	0.300	0 ==> 2
	58	1	0.250	0 ==> 2
	59	1	0.429	0 --> 2

	67	1	0.286	0 ==> 2
node_77 --> node_76	5	1	0.143	0 ==> 1
	46	1	1.000	2 ==> 3
	59	1	0.429	2 --> 3
node_76 --> node_72	43	1	0.250	2 ==> 1
	45	1	0.500	1 ==> 0
	48	1	0.250	1 --> 0
	97	1	1.000	0 --> 1
node_72 --> node_54	25	1	0.400	0 --> 2
	32	1	0.429	1 ==> 2
	67	1	0.286	2 ==> 1
	81	1	0.400	0 --> 2
node_54 --> Cimexomys	34	1	0.500	1 ==> 0
	48	1	0.250	0 --> 2
	59	1	0.429	3 ==> 2
node_54 --> Meniscoes.	29	1	0.286	1 ==> 2
	31	1	0.286	1 ==> 0
	61	1	0.200	1 ==> 2
	72	1	0.250	1 ==> 2
	74	1	0.500	0 ==> 1
	80	1	0.333	1 ==> 0
node_72 --> node_71	16	1	0.286	0 ==> 2
	35	1	0.333	0 --> 1
	76	1	0.250	1 ==> 0
	84	1	0.667	1 ==> 3
	86	1	0.200	1 --> 0
node_71 --> node_63	5	1	0.143	1 ==> 0
	9	1	0.200	0 ==> 1
	38	1	0.600	3 --> 2
	47	1	0.429	0 ==> 1
	83	1	0.667	0 ==> 1
	98	1	0.500	0 --> 1
	100	1	1.000	0 --> 1
node_63 --> node_56	16	1	0.286	2 ==> 1
	43	1	0.250	1 --> 0
node_56 --> Boffius	55	1	0.300	2 ==> 3
node_56 --> node_55	32	1	0.429	1 --> 2
	58	1	0.250	2 ==> 1
node_55 --> Pentacos.	7	1	0.143	0 ==> 1
	31	1	0.286	1 ==> 0
	67	1	0.286	2 ==> 0
	82	1	0.667	1 ==> 2

node_55 --> Microcos.	35	1	0.333	1 --> 0
	48	1	0.250	0 ==> 2
node_63 --> node_62	19	1	0.167	1 --> 0
	25	1	0.400	0 --> 1
	30	1	0.750	2 ==> 3
	31	1	0.286	1 ==> 0
	41	1	0.500	1 --> 0
	44	1	0.250	1 --> 0
	73	1	0.333	1 --> 0
node_62 --> Kogaionon	29	1	0.286	1 ==> 0
	55	1	0.300	2 ==> 1
	59	1	0.429	3 ==> 2
	61	1	0.200	1 ==> 0
node_62 --> node_61	18	1	0.333	1 --> 0
	20	1	0.667	1 --> 0
	21	1	0.667	1 --> 0
	25	1	0.400	1 --> 2
	32	1	0.429	1 ==> 3
	86	1	0.200	0 ==> 1
	88	1	0.500	0 ==> 1
	90	1	1.000	0 ==> 2
	102	1	0.500	0 --> 1
node_61 --> node_60	26	1	0.500	1 ==> 3
	27	1	1.000	0 ==> 1
	29	1	0.286	1 ==> 2
	42	1	0.600	2 ==> 3
	51	1	0.600	2 ==> 3
	55	1	0.300	2 ==> 3
	67	1	0.286	2 --> 0
	92	1	1.000	0 ==> 1
	93	1	0.333	1 ==> 0
node_60 --> node_57	19	1	0.167	0 --> 1
	63	1	1.000	3 ==> 4
	65	1	1.000	0 ==> 2
	74	1	0.500	0 ==> 2
node_57 --> Taeniolab.	67	1	0.286	0 --> 2
	72	1	0.250	1 ==> 2
node_57 --> Catopsalis	31	1	0.286	0 ==> 1
node_60 --> node_59	6	1	0.500	0 --> 1
	7	1	0.143	0 --> 1
	47	1	0.429	1 ==> 0
	61	1	0.200	1 ==> 0

	62	1	0.667	2 --> 1
node_59 --> node_58	53	1	0.667	0 ==> 2
	68	1	0.667	1 ==> 2
	69	1	0.667	1 ==> 2
	73	1	0.333	0 ==> 1
	74	1	0.500	0 ==> 1
node_61 --> Yubaatar	3	1	0.500	2 ==> 1
	72	1	0.250	1 ==> 2
	81	1	0.400	0 ==> {12}
	84	1	0.667	3 ==> 4
node_71 --> node_70	48	1	0.250	0 --> 1
	85	1	0.500	0 ==> 2
	87	1	1.000	0 --> 1
	90	1	1.000	0 --> 1
	91	1	1.000	0 --> 1
	94	1	0.500	0 ==> 1
	99	1	0.500	1 --> 0
node_70 --> node_65	25	1	0.400	0 --> 1
	43	1	0.250	1 ==> 2
	58	1	0.250	2 --> 1
	81	1	0.400	0 --> 2
node_65 --> node_64	3	1	0.500	2 ==> 1
node_64 --> Buginbaat.	16	1	0.286	2 ==> 0
	32	1	0.429	1 ==> 3
	43	1	0.250	2 ==> 0
	44	1	0.250	1 ==> 0
	48	1	0.250	1 ==> 0
	55	1	0.300	2 ==> 3
	61	1	0.200	1 ==> 2
node_64 --> Stygimys	31	1	0.286	1 ==> 2
	47	1	0.429	0 ==> 1
node_65 --> Eucosmodon	7	1	0.143	0 ==> 1
	26	1	0.500	1 ==> 2
	67	1	0.286	2 ==> 1
	72	1	0.250	1 ==> 2
node_70 --> node_69	19	1	0.167	1 ==> 0
	21	1	0.667	1 ==> 2
	32	1	0.429	1 ==> 2
	35	1	0.333	1 --> 0
	55	1	0.300	2 --> 1
	59	1	0.429	3 --> 1
	67	1	0.286	2 ==> 0

	82	1	0.667	1 ==> 2
node_69 --> node_67	59	1	0.429	1 --> 2
	61	1	0.200	1 ==> 2
	85	1	0.500	2 ==> 1
node_67 --> node_66	83	1	0.667	0 ==> 2
	84	1	0.667	3 ==> 2
	88	1	0.500	0 ==> 1
	89	1	0.500	0 ==> 1
	98	1	0.500	0 ==> 1
	101	1	0.500	1 ==> 0
	102	1	0.500	0 ==> 1
node_66 --> Catopsb.	5	1	0.143	1 ==> 0
	7	1	0.143	0 ==> 1
	9	1	0.200	0 ==> 1
	26	1	0.500	1 ==> 2
	29	1	0.286	1 ==> 2
	42	1	0.600	2 ==> 1
	43	1	0.250	1 ==> 0
	44	1	0.250	1 ==> 0
	48	1	0.250	1 ==> 2
	51	1	0.600	2 ==> 3
	55	1	0.300	1 --> 2
	59	1	0.429	2 --> 3
	61	1	0.200	2 ==> 0
	85	1	0.500	1 ==> 0
	86	1	0.200	0 ==> 1
	95	1	0.500	1 ==> 0
node_66 --> Kryptob.	58	1	0.250	2 ==> 0
node_67 --> Nemegtb.	25	1	0.400	0 ==> 1
	81	1	0.400	0 ==> 2
	96	1	0.333	1 ==> 0
node_69 --> node_68	58	1	0.250	2 ==> 0
node_68 --> Kamptob.	6	1	0.500	0 ==> 1
	9	1	0.200	0 ==> 1
	16	1	0.286	2 ==> 1
	96	1	0.333	1 ==> 0
node_68 --> Chulsanb.	7	1	0.143	0 ==> 1
	84	1	0.667	3 ==> 2
node_76 --> node_75	15	1	0.500	0 --> 1
	58	1	0.250	2 --> 1
	77	1	0.500	1 ==> 2
	81	1	0.400	0 --> 1

	84	1	0.667	1 --> 2
	93	1	0.333	1 --> 0
node_75 --> node_74	19	1	0.167	1 ==> 0
	31	1	0.286	1 ==> 2
	48	1	0.250	1 ==> 3
	55	1	0.300	2 --> 3
	72	1	0.250	1 ==> 2
node_74 --> node_73	5	1	0.143	1 ==> 0
	64	1	0.500	0 ==> 1
node_73 --> Ectypodus	9	1	0.200	0 ==> 1
	83	1	0.667	0 ==> 2
node_73 --> Ptilodus	29	1	0.286	1 ==> 0
	48	1	0.250	3 ==> 2
	86	1	0.200	1 --> 0
	96	1	0.333	1 ==> 0
node_74 --> Neoliotom.	15	1	0.500	1 --> 0
	16	1	0.286	0 ==> 2
	58	1	0.250	1 --> 2
	74	1	0.500	0 ==> 1
node_77 --> Cimolodon	61	1	0.200	1 --> 2
	72	1	0.250	1 ==> 2

Character diagnostics:

Character	Range	Min steps	Tree steps	Max steps	CI	RI	RC	HI	G-fit
1	1	1	1	3	1.000	1.000	1.000	0.000	1.000
2	1	1	1	3	1.000	1.000	1.000	0.000	1.000
3	2	2	4	17	0.500	0.867	0.433	0.500	0.600
4	1	1	2	3	0.500	0.500	0.250	0.500	0.750
5	1	1	7	17	0.143	0.625	0.089	0.857	0.333
6	1	1	2	2	0.500	0.000	0.000	0.500	0.750
7	1	1	7	11	0.143	0.400	0.057	0.857	0.333
8	1	1	1	3	1.000	1.000	1.000	0.000	1.000
9	1	1	5	12	0.200	0.636	0.127	0.800	0.429
10	1	1	1	3	1.000	1.000	1.000	0.000	1.000
11	1	1	1	4	1.000	1.000	1.000	0.000	1.000
12	1	1	1	2	1.000	1.000	1.000	0.000	1.000
13	1	1	2	6	0.500	0.800	0.400	0.500	0.750
14	1	1	1	3	1.000	1.000	1.000	0.000	1.000
15	1	1	2	3	0.500	0.500	0.250	0.500	0.750

16	2	2	7	19	0.286	0.706	0.202	0.714	0.375
17	2	2	2	9	1.000	1.000	1.000	0.000	1.000
18	1	1	3	9	0.333	0.750	0.250	0.667	0.600
19	1	1	6	12	0.167	0.545	0.091	0.833	0.375
20	2	2	3	10	0.667	0.875	0.583	0.333	0.750
21	2	2	3	12	0.667	0.900	0.600	0.333	0.750
22	2	2	3	9	0.667	0.857	0.571	0.333	0.750
23	1	1	1	3	1.000	1.000	1.000	0.000	1.000
24	1	1	1	3	1.000	1.000	1.000	0.000	1.000
25	2	2	5	8	0.400	0.500	0.200	0.600	0.500
26	3	3	6	19	0.500	0.813	0.406	0.500	0.500
27	1	1	1	4	1.000	1.000	1.000	0.000	1.000
28	1	1	2	6	0.500	0.800	0.400	0.500	0.750
29	2	2	7	21	0.286	0.737	0.211	0.714	0.375
30	3	3	4	14	0.750	0.909	0.682	0.250	0.750
31	2	2	7	16	0.286	0.643	0.184	0.714	0.375
32	3	3	7	23	0.429	0.800	0.343	0.571	0.429
33	1	1	2	12	0.500	0.909	0.455	0.500	0.750
34	1	1	2	17	0.500	0.938	0.469	0.500	0.750
35	1	1	3	10	0.333	0.778	0.259	0.667	0.600
36	1	1	2	11	0.500	0.900	0.450	0.500	0.750
37	1	1	2	14	0.500	0.923	0.462	0.500	0.750
38	3	3	5	18	0.600	0.867	0.520	0.400	0.600
39	1	1	1	4	1.000	1.000	1.000	0.000	1.000
40	1	1	1	15	1.000	1.000	1.000	0.000	1.000
41	1	1	2	10	0.500	0.889	0.444	0.500	0.750
42	3	3	5	20	0.600	0.882	0.529	0.400	0.600
43	2	2	8	18	0.250	0.625	0.156	0.750	0.333
44	1	1	4	18	0.250	0.824	0.206	0.750	0.500
45	1	1	2	16	0.500	0.933	0.467	0.500	0.750
46	3	3	3	16	1.000	1.000	1.000	0.000	1.000
47	3	3	7	17	0.429	0.714	0.306	0.571	0.429
48	3	3	12	22	0.250	0.526	0.132	0.750	0.250
49	1	1	1	3	1.000	1.000	1.000	0.000	1.000
50	1	1	1	3	1.000	1.000	1.000	0.000	1.000
51	3	3	5	18	0.600	0.867	0.520	0.400	0.600
52	2	2	2	12	1.000	1.000	1.000	0.000	1.000
53	2	2	3	8	0.667	0.833	0.556	0.333	0.750
54	1	1	1	8	1.000	1.000	1.000	0.000	1.000
55	3	3	10	24	0.300	0.667	0.200	0.700	0.300
56	1	1	2	5	0.500	0.750	0.375	0.500	0.750
57	1	1	2	7	0.500	0.833	0.417	0.500	0.750

58	2	2	8	15	0.250	0.538	0.135	0.750	0.333
59	3	3	7	12	0.429	0.556	0.238	0.571	0.429
60	1	1	1	4	1.000	1.000	1.000	0.000	1.000
61	2	2	10	16	0.200	0.429	0.086	0.800	0.273
62	2	2	3	10	0.667	0.875	0.583	0.333	0.750
63	4	4	4	8	1.000	1.000	1.000	0.000	1.000
64	1	1	2	4	0.500	0.667	0.333	0.500	0.750
65	2	2	2	4	1.000	1.000	1.000	0.000	1.000
66	2	2	2	5	1.000	1.000	1.000	0.000	1.000
67	2	2	7	13	0.286	0.545	0.156	0.714	0.375
68	2	2	3	9	0.667	0.857	0.571	0.333	0.750
69	2	2	3	10	0.667	0.875	0.583	0.333	0.750
70	1	1	2	6	0.500	0.800	0.400	0.500	0.750
71	1	1	1	4	1.000	1.000	1.000	0.000	1.000
72	2	2	8	14	0.250	0.500	0.125	0.750	0.333
73	1	1	3	9	0.333	0.750	0.250	0.667	0.600
74	2	2	4	6	0.500	0.500	0.250	0.500	0.600
75	2	2	2	5	1.000	1.000	1.000	0.000	1.000
76	1	1	4	17	0.250	0.813	0.203	0.750	0.500
77	2	2	4	12	0.500	0.800	0.400	0.500	0.600
78	1	1	1	1	1.000	0/0	0/0	0.000	1.000
79	1	1	1	1	1.000	0/0	0/0	0.000	1.000
80	1	1	3	11	0.333	0.800	0.267	0.667	0.600
81	2	2	5	6	0.400	0.250	0.100	0.600	0.500
82	2	2	3	9	0.667	0.857	0.571	0.333	0.750
83	2	2	3	8	0.667	0.833	0.556	0.333	0.750
84	4	4	6	10	0.667	0.667	0.444	0.333	0.600
85	2	2	4	7	0.500	0.600	0.300	0.500	0.600
86	1	1	5	9	0.200	0.500	0.100	0.800	0.429
87	1	1	1	5	1.000	1.000	1.000	0.000	1.000
88	1	1	2	5	0.500	0.750	0.375	0.500	0.750
89	1	1	2	4	0.500	0.667	0.333	0.500	0.750
90	2	2	2	9	1.000	1.000	1.000	0.000	1.000
91	1	1	1	5	1.000	1.000	1.000	0.000	1.000
92	1	1	1	2	1.000	1.000	1.000	0.000	1.000
93	1	1	3	5	0.333	0.500	0.167	0.667	0.600
94	1	1	2	7	0.500	0.833	0.417	0.500	0.750
95	1	1	2	3	0.500	0.500	0.250	0.500	0.750
96	1	1	3	3	0.333	0.000	0.000	0.667	0.600
97	1	1	1	5	1.000	1.000	1.000	0.000	1.000
98	1	1	2	5	0.500	0.750	0.375	0.500	0.750
99	1	1	2	6	0.500	0.800	0.400	0.500	0.750

100	1	1	1	3	1.000	1.000	1.000	0.000	1.000
101	1	1	2	4	0.500	0.667	0.333	0.500	0.750
102	1	1	2	4	0.500	0.667	0.333	0.500	0.750

IV. Phylogenetic Analyses with 19 characters ordered.

```
Consensus of 69 trees

Source of trees from which consensus(es) calculated...
Heuristic search settings:
  Optimality criterion = parsimony
    Character-status summary:
      Of 102 total characters:
        19 characters are of type 'ord' (Wagner)
        83 characters are of type 'unord'
        All characters have equal weight
        2 characters are parsimony-uninformative
        Number of parsimony-informative characters = 100
    Gaps are treated as "missing"
    Multistate taxa interpreted as polymorphism
  Starting tree(s) obtained via stepwise addition
  Addition sequence: random
    Number of replicates = 1000
    Starting seed = 1506288718
  Number of trees held at each step during stepwise addition = 10
  Branch-swapping algorithm: tree-bisection-reconnection (TBR)
  Steepest descent option not in effect
  Initial 'MaxTrees' setting = 10000 (will be auto-increased by 100)
  Branches collapsed (creating polytomies) if maximum branch length is zero
  'MulTrees' option not in effect; only 1 tree will be saved per replicate
  Topological constraints not enforced
  Trees are unrooted
```

Tree description:

```
  Unrooted tree(s) rooted using outgroup method
  Optimality criterion = parsimony
    Character-status summary:
      Of 102 total characters:
        19 characters are of type 'ord' (Wagner)
        83 characters are of type 'unord'
        All characters have equal weight
        2 characters are parsimony-uninformative
        Number of parsimony-informative characters = 100
    Gaps are treated as "missing"
```

Multistate taxa interpreted as polymorphism ("min" values for CI, RI, and
RC are minimum-possible character lengths)

Character-state optimization: Accelerated transformation (ACCTRAN)

Figure 3 - Strict Consensus Tree (of 69 EMTs) with 19 characters ordered

Tree length = 421

Consistency index (CI) = 0.4276

Homoplasy index (HI) = 0.6223

CI excluding uninformative characters = 0.4248

HI excluding uninformative characters = 0.5752

Retention index (RI) = 0.7217

Rescaled consistency index (RC) = 0.3086

```
/------------------------------------------------------------- Sinoconodon
|
+------------------------------------------------------------- Morganucodon
|
|   /--------------------------------------------------------- Thomasia
|   |
|   +--------------------------------------------------------- Haramiyavia
|   |
|   |                                       /--------- Meketichoffatia
|   |                                       |
68  |                                       |   /---- Henkelodon
|   |                                       +---45
|   |                                       |   \---- Kuehneodon
|   |                                       |
|   |   /-----------------------------------------46--------- Rugosodon
|   |   |                                   |
|   |   |                                   +--------- Paulchoffatia
|   |   |                                   |
|   |   |                                   +--------- Meketibolodon
\--67   |                                   |
    |   |                                   \--------- Guimarotodon
    |   |
    |   |  /------------------------------------------------ Ctenacodon
    |   |  |
    |   |  +------------------------------------------------ Glirodon
    |   |  |
    |   |  +------------------------------------------------ Bolodon
    |   |  |
    |   |  |   /-------------------------------------------- Plagiaulax
    |   |  |   |
    |   |  |   |                                /---- Sinobaatar
    |   |  |   |   /---------------------------------47
    |   |  |   |   |                            \---- Eobaatar
    |   |  |   |   |
    \---66 |   |   |   /----------------------------------- Arginbaatar
        |  +--64   |   |
        |  |   |   |   |      /-------------------------- Cimexomys
        |  |   |   |   |      |
        |  |   |   |   |      +-------------------------- Boffius
        |  |   |   |   |      |
```

```
 |    |    |     |    |        +------------------------- Meniscoessus
 |    |    |     |    |        |
 |    |    \---63     |        |   /---------------------- Buginbaatar
 |    |          |    |        |   |
 |    |          |    |        |   |              /---- Taeniolabis
 |    |          |    |        |   |       /-------48
 |    |          |    |        |   |       |      \---- Catopsalis
 |    |          |    |        +--53       |
 |    |          |    |        |   |   /--51       /---- Lambdopsalis
 |    |          |    |        |   |   |   |   /---49
 |    |          |    |        |   |   |   |   |    \---- Sphenopsalis
 |    |          |    |        |   \---52  \--50
 |    |          |    |        |       |      \--------- Prionessus
 |    |          \--62         |       |
 \--65                |        |       \----------------- Yubaatar
      |               |        |
      |               |        +------------------------- Cimolodon
      |               |        |
      |               |        +------------------------- Ectypodus
      |               |    /--60
      |               |    |   +------------------------- Mesodma
      |               |    |   |
      |               |    |   +------------------------- Ptilodus
      |               |    |   |
      |               |    |   +------------------------- Neoliotomus
      |               |    |   |
      |               |    |   |                  /---- Pentacosmodon
      |               |    |   |             /---54
      |               |    |   |             |    \---- Catopsbaatar
      |               |    |   |         /--55
      |               |    |   |         |   \--------- Kryptobaatar
      |               |    |   |     /--57
      |               \---61   |     |   |       /---- Kamptobaatar
      |                    |   |     |   \-------56
      |                    |   +-------58          \---- Chulsanbaatar
      |                    |   |     |
      |                    |   |     \----------------- Nemegtbaatar
      |                    |   |
      |                    |   |                  /---- Eucosmodon
      |                    |   +-------------------59
      |                    |   |                  \---- Stygimys
      |                    |   |
      |                    |   \------------------------- Microcosmodon
      |                    |
      |                    \------------------------------ Kogaionon
      |
      \---------------------------------------------- Zofiabaatar
```

Apomorphy lists:

Branch	Character	Steps	CI	Change
node_68 --> Sinoconod.	78	1	1.000	1 ==> 0
	79	1	1.000	1 ==> 0
node_68 --> Morganuc.	84	1	0.571	1 ==> 0
node_68 --> node_67	4	1	0.500	0 --> 1
	5	1	0.100	0 --> 1
	12	1	1.000	0 ==> 1

	22	1	0.667	0 ==> 1
	42	1	0.600	0 --> 1
	53	1	0.667	0 ==> 1
	62	1	0.750	0 ==> 1
	63	1	1.000	0 ==> 3
	66	1	0.667	0 --> 1
	68	1	0.667	0 ==> 1
	69	1	0.667	0 ==> 2
	77	1	0.250	0 --> 1
	89	1	0.500	1 --> 0
	93	1	0.333	0 --> 1
	95	1	0.500	0 --> 1
	101	1	0.500	0 --> 1
node_67 --> Thomasia	26	3	0.333	0 ==> 3
	32	1	0.444	0 ==> 1
	66	1	0.667	1 --> 0
node_67 --> Haramiy.	18	1	0.600	0 ==> 1
	42	1	0.600	1 --> 0
node_67 --> node_66	1	1	1.000	0 ==> 1
	2	1	1.000	0 ==> 1
	3	1	0.400	0 ==> 1
	8	1	1.000	0 ==> 1
	10	1	1.000	0 ==> 1
	11	1	1.000	0 ==> 1
	14	1	1.000	0 ==> 1
	17	1	1.000	0 ==> 1
	19	1	0.143	0 ==> 1
	20	1	0.667	0 ==> 1
	24	1	1.000	0 ==> 1
	28	1	0.500	0 ==> 1
	29	2	0.286	2 ==> 0
	36	1	0.500	0 ==> 1
	38	1	0.600	0 ==> 1
	41	1	0.500	0 ==> 1
	45	1	0.143	0 ==> 1
	47	2	0.273	0 ==> 2
	48	1	0.176	0 ==> 1
	49	1	1.000	0 ==> 1
	50	1	1.000	0 ==> 1
	51	1	0.600	0 ==> 1
	52	1	1.000	0 ==> 1
	56	1	0.500	0 ==> 1

	60	1	1.000	0 ==> 1
	61	1	0.273	1 ==> 0
	72	1	0.182	2 ==> 1
node_66 --> node_46	23	1	1.000	0 ==> 1
	46	1	0.750	1 ==> 0
	63	1	1.000	3 ==> 1
	64	1	0.333	0 ==> 1
	65	1	1.000	0 ==> 1
	66	1	0.667	1 --> 2
	68	1	0.667	1 ==> 2
	70	1	0.500	0 ==> 1
	71	1	1.000	0 ==> 1
	72	1	0.182	1 ==> 0
	75	1	1.000	0 ==> 2
	76	1	0.111	0 ==> 1
	94	1	0.333	0 --> 1
node_46 --> Meketich.	56	1	0.500	1 ==> 0
node_46 --> node_45	4	1	0.500	1 --> 0
	13	1	0.333	0 --> 1
	20	1	0.667	1 ==> 2
	63	1	1.000	1 ==> 2
	72	1	0.182	0 --> 1
node_45 --> Henkelodon	22	1	0.667	1 ==> 2
node_45 --> Kuehneodon	26	1	0.333	0 ==> 1
	51	1	0.600	1 ==> 2
node_46 --> Rugosodon	13	1	0.333	0 ==> 1
	75	1	1.000	2 ==> 1
node_46 --> Meketib.	47	1	0.273	2 ==> 3
node_46 --> Guimarot.	47	1	0.273	2 ==> 3
node_66 --> node_65	5	1	0.100	1 --> 0
	7	1	0.167	0 ==> 1
	9	1	0.250	0 ==> 1
	13	1	0.333	0 ==> 1
	30	1	0.667	0 ==> 1
	39	1	1.000	0 --> 1
	53	1	0.667	1 ==> 0
	54	1	1.000	0 ==> 1
	57	1	0.500	0 ==> 1
	62	1	0.750	1 ==> 2
	69	1	0.667	2 ==> 1
	73	1	0.500	0 ==> 1
	77	1	0.250	1 --> 0

```
                         82           1  1.000  0 ==> 1
                         85           1  0.286  0 --> 1
                         97           1  0.333  0 --> 1
                         99           1  0.500  0 --> 1
node_65 --> Ctenacodon   55           1  0.533  0 ==> 1
node_65 --> Glirodon     16           1  0.200  0 ==> 1
                         57           1  0.500  1 ==> 0
node_65 --> Bolodon      5            1  0.100  0 ==> 1
                         38           1  0.600  1 ==> 2
                         76           1  0.111  0 ==> 1
node_65 --> node_64      17           1  1.000  1 --> 2
                         18           1  0.600  0 --> 1
                         19           1  0.143  1 --> 0
                         21           1  0.667  0 --> 1
                         22           1  0.667  1 --> 2
                         28           1  0.500  1 --> 0
                         30           1  0.667  1 --> 0
                         32           1  0.444  0 --> 1
                         33           1  1.000  0 ==> 1
                         38           1  0.600  1 ==> 2
                         43           1  0.200  0 ==> 1
                         48           1  0.176  1 --> 2
                         58           1  0.154  0 --> 1
                         80           1  0.333  0 --> 1
                         85           1  0.286  1 --> 0
node_64 --> node_63      9            1  0.250  1 --> 0
                         37           1  0.500  0 --> 1
                         42           1  0.600  1 ==> 2
                         44           1  0.333  0 ==> 1
                         46           1  0.750  1 ==> 2
                         47           1  0.273  2 ==> 1
                         52           1  1.000  1 ==> 2
                         77           1  0.250  0 --> 1
node_63 --> node_47      61           1  0.273  0 ==> 1
                         70           1  0.500  0 ==> 1
                         76           1  0.111  0 ==> 1
                         86           1  0.167  0 --> 1
node_47 --> Sinobaatar   30           1  0.667  0 --> 2
                         55           1  0.533  0 ==> 1
                         77           1  0.250  1 --> 0
node_47 --> Eobaatar     16           1  0.200  0 ==> 2
                         37           1  0.500  1 --> 0
```

node_63 --> node_62	3	1	0.400	1	==>	2
	7	1	0.167	1	-->	0
	25	1	0.286	0	-->	1
	36	1	0.500	1	==>	0
	40	1	1.000	0	==>	1
	43	1	0.200	1	==>	2
	47	1	0.273	1	==>	0
	84	1	0.571	1	-->	3
node_62 --> Arginbaat.	48	1	0.176	2	==>	3
	58	1	0.154	1	-->	0
	80	1	0.333	1	-->	0
node_62 --> node_61	5	1	0.100	0	-->	1
	26	1	0.333	0	==>	1
	30	1	0.667	0	-->	2
	34	1	0.500	0	-->	1
	38	1	0.600	2	-->	3
	45	1	0.143	1	-->	0
	46	1	0.750	2	-->	3
	48	1	0.176	2	-->	1
	51	1	0.600	1	==>	2
	55	1	0.533	0	==>	1
	58	1	0.154	1	-->	2
	59	2	0.444	0	==>	2
	67	1	0.375	0	-->	2
node_61 --> node_60	29	1	0.286	0	==>	1
	31	1	0.222	0	==>	1
	55	1	0.533	1	==>	2
	59	1	0.444	2	==>	3
	61	1	0.273	0	==>	1
	76	1	0.111	0	==>	1
	81	1	0.429	0	-->	2
node_60 --> Cimexomys	32	1	0.444	1	==>	2
	34	1	0.500	1	==>	0
	43	1	0.200	2	==>	1
	48	1	0.176	1	-->	2
	59	1	0.444	3	==>	2
	67	1	0.375	2	==>	1
	84	1	0.571	3	==>	1
	86	1	0.167	0	==>	1
node_60 --> Boffius	16	1	0.200	0	==>	1
	55	1	0.533	2	==>	3
	76	1	0.111	1	==>	0

node_60 --> Meniscoes.	19	1	0.143	0 ==> 1
	25	1	0.286	1 ==> 2
	29	1	0.286	1 ==> 2
	31	1	0.222	1 ==> 0
	32	1	0.444	1 ==> 2
	43	1	0.200	2 ==> 1
	48	1	0.176	1 ==> 0
	61	1	0.273	1 ==> 2
	67	1	0.375	2 ==> 1
	72	1	0.182	1 ==> 2
	74	1	0.500	0 ==> 1
	80	1	0.333	1 ==> 0
	86	1	0.167	0 ==> 1
node_60 --> node_53	3	1	0.400	2 --> 1
	9	1	0.250	0 --> 1
	18	1	0.600	1 --> 0
	20	1	0.667	1 --> 0
	21	1	0.667	1 --> 0
	25	1	0.286	1 --> 2
	32	2	0.444	1 ==> 3
	35	1	0.333	0 ==> 1
	43	1	0.200	2 ==> 1
	44	1	0.333	1 ==> 0
	48	1	0.176	1 ==> 0
	55	1	0.533	2 --> 3
	76	1	0.111	1 ==> 0
	83	1	0.400	0 --> 1
	86	1	0.167	0 --> 1
	88	1	0.500	0 --> 1
	90	1	1.000	0 --> 2
	98	1	0.500	0 --> 1
	100	1	1.000	0 --> 1
	102	1	0.500	0 --> 1
node_53 --> Buginbaat.	43	1	0.200	1 ==> 0
	58	1	0.154	2 --> 1
	61	1	0.273	1 ==> 2
node_53 --> node_52	5	1	0.100	1 --> 0
	16	1	0.200	0 ==> 2
	30	1	0.667	2 --> 3
	31	1	0.222	1 ==> 0
	41	1	0.500	1 ==> 0
	47	1	0.273	0 --> 1

	73	1	0.500	1	==>	0
node_52 --> node_51	3	1	0.400	1	-->	2
	26	2	0.333	1	==>	3
	27	1	1.000	0	==>	1
	29	1	0.286	1	==>	2
	42	1	0.600	2	==>	3
	51	1	0.600	2	==>	3
	67	1	0.375	2	-->	0
	81	1	0.429	2	-->	0
	92	1	1.000	0	==>	1
	93	1	0.333	1	==>	0
node_51 --> node_48	19	1	0.143	0	==>	1
	63	1	1.000	3	==>	4
	65	1	1.000	0	==>	2
	74	1	0.500	0	==>	2
node_48 --> Taeniolab.	67	1	0.375	0	-->	2
	72	1	0.182	1	==>	2
node_48 --> Catopsalis	31	1	0.222	0	==>	1
node_51 --> node_50	6	1	0.500	0	-->	1
	7	1	0.167	0	-->	1
	47	1	0.273	1	-->	0
	61	1	0.273	1	==>	0
	62	1	0.750	2	-->	1
node_50 --> node_49	53	1	0.667	0	==>	2
	68	1	0.667	1	==>	2
	69	1	0.667	1	==>	2
	73	1	0.500	0	==>	1
	74	1	0.500	0	==>	1
node_52 --> Yubaatar	55	1	0.533	3	-->	2
	72	1	0.182	1	==>	2
	84	1	0.571	3	==>	4
node_60 --> Cimolodon	5	1	0.100	1	-->	0
	45	1	0.143	0	-->	1
	46	1	0.750	3	==>	2
	59	1	0.444	3	==>	2
	61	1	0.273	1	==>	2
	72	1	0.182	1	==>	2
node_60 --> Ectypodus	5	1	0.100	1	-->	0
	9	1	0.250	0	-->	1
	15	1	0.333	0	==>	1
	25	1	0.286	1	-->	0
	31	1	0.222	1	==>	2

	45	1	0.143	0	-->	1
	48	2	0.176	1	==>	3
	58	1	0.154	2	-->	1
	64	1	0.333	0	==>	1
	72	1	0.182	1	==>	2
	77	1	0.250	1	==>	2
	81	1	0.429	2	==>	1
	83	1	0.400	0	==>	2
	86	1	0.167	0	==>	1
	97	1	0.333	1	-->	0
node_60 --> Mesodma	15	1	0.333	0	==>	1
	19	1	0.143	0	==>	1
	45	1	0.143	0	-->	1
	58	1	0.154	2	-->	1
	77	1	0.250	1	==>	2
node_60 --> Ptilodus	5	1	0.100	1	-->	0
	15	1	0.333	0	==>	1
	25	1	0.286	1	-->	0
	29	1	0.286	1	==>	0
	31	1	0.222	1	==>	2
	45	1	0.143	0	-->	1
	48	1	0.176	1	-->	2
	58	1	0.154	2	-->	1
	64	1	0.333	0	==>	1
	72	1	0.182	1	==>	2
	77	1	0.250	1	==>	2
	81	1	0.429	2	==>	1
	84	1	0.571	3	==>	2
	93	1	0.333	1	==>	0
	96	1	0.333	1	==>	0
	97	1	0.333	1	-->	0
node_60 --> Neoliotom.	16	1	0.200	0	==>	2
	31	1	0.222	1	==>	2
	45	1	0.143	0	-->	1
	48	2	0.176	1	==>	3
	55	1	0.533	2	==>	3
	72	1	0.182	1	==>	2
	74	1	0.500	0	==>	1
	77	1	0.250	1	==>	2
node_60 --> node_58	16	1	0.200	0	==>	2
	21	1	0.667	1	==>	2
	32	1	0.444	1	==>	2

	43	1	0.200	2 ==> 1
	55	1	0.533	2 --> 1
	59	1	0.444	3 ==> 2
	67	1	0.375	2 ==> 0
	76	1	0.111	1 ==> 0
	82	1	1.000	1 ==> 2
	85	1	0.286	0 ==> 1
	87	1	1.000	0 ==> 1
	90	1	1.000	0 ==> 1
	91	1	1.000	0 ==> 1
	94	1	0.333	0 ==> 1
	99	1	0.500	1 ==> 0
node_58 --> node_57	25	1	0.286	1 --> 0
	58	2	0.154	2 --> 0
	81	1	0.429	2 --> 0
	84	1	0.571	3 --> 2
node_57 --> node_55	83	1	0.400	0 ==> 2
	88	1	0.500	0 ==> 1
	89	1	0.500	0 ==> 1
	98	1	0.500	0 ==> 1
	101	1	0.500	1 ==> 0
	102	1	0.500	0 ==> 1
node_55 --> node_54	5	1	0.100	1 ==> 0
	7	1	0.167	0 ==> 1
	9	1	0.250	0 --> 1
	26	1	0.333	1 --> 2
	29	1	0.286	1 --> 2
	43	1	0.200	1 ==> 0
	51	1	0.600	2 --> 3
	55	1	0.533	1 --> 2
	58	1	0.154	0 --> 1
	59	1	0.444	2 --> 3
	61	1	0.273	1 --> 0
	85	1	0.286	1 --> 0
	86	1	0.167	0 --> 1
	95	1	0.500	1 --> 0
node_54 --> Pentacos.	16	1	0.200	2 ==> 1
	31	1	0.222	1 ==> 0
	35	1	0.333	0 ==> 1
	47	1	0.273	0 ==> 1
	48	1	0.176	1 ==> 0
node_54 --> Catopsb.	42	1	0.600	2 ==> 1

	44	1	0.333	1 ==> 0
	48	1	0.176	1 ==> 2
	58	1	0.154	1 ==> 2
node_55 --> Kryptob.	61	1	0.273	1 ==> 2
node_57 --> node_56	59	1	0.444	2 ==> 1
	85	1	0.286	1 ==> 2
node_56 --> Kamptob.	6	1	0.500	0 ==> 1
	9	1	0.250	0 --> 1
	16	1	0.200	2 ==> 1
	84	1	0.571	2 --> 3
	96	1	0.333	1 ==> 0
node_56 --> Chulsanb.	7	1	0.167	0 ==> 1
node_58 --> Nemegtb.	61	1	0.273	1 ==> 2
	96	1	0.333	1 ==> 0
node_60 --> node_59	16	1	0.200	0 ==> 2
	19	1	0.143	0 ==> 1
	31	1	0.222	1 --> 2
	35	1	0.333	0 ==> 1
	58	1	0.154	2 --> 1
	76	1	0.111	1 ==> 0
	85	2	0.286	0 --> 2
	94	1	0.333	0 --> 1
node_59 --> Eucosmod.	7	1	0.167	0 ==> 1
	26	1	0.333	1 ==> 2
	67	1	0.375	2 ==> 1
	72	1	0.182	1 ==> 2
node_59 --> Stygimys	3	1	0.400	2 ==> 1
	47	1	0.273	0 ==> 1
node_60 --> Microcos.	5	1	0.100	1 --> 0
	9	1	0.250	0 --> 1
	16	1	0.200	0 ==> 1
	19	1	0.143	0 ==> 1
	25	1	0.286	1 --> 0
	32	1	0.444	1 ==> 2
	38	1	0.600	3 ==> 2
	43	2	0.200	2 ==> 0
	47	1	0.273	0 ==> 1
	48	1	0.176	1 --> 2
	58	1	0.154	2 --> 1
	76	1	0.111	1 ==> 0
	81	1	0.429	2 --> 0
	83	1	0.400	0 ==> 1

```
node_61 --> Kogaionon   30          1  0.667  2 --> 3
                        83          1  0.400  0 ==> 1
node_65 --> Zofiabaatar    48          2  0.176  1 ==> 3
```

Character diagnostics:

Character	Range	Min steps	Tree steps	Max steps	CI	RI	RC	HI	G-fit
1	1	1	1	3	1.000	1.000	1.000	0.000	1.000
2	1	1	1	3	1.000	1.000	1.000	0.000	1.000
3	2	2	5	17	0.400	0.800	0.320	0.600	0.500
4	1	1	2	3	0.500	0.500	0.250	0.500	0.750
5	1	1	10	17	0.100	0.438	0.044	0.900	0.250
6	1	1	2	2	0.500	0.000	0.000	0.500	0.750
7	1	1	6	11	0.167	0.500	0.083	0.833	0.375
8	1	1	1	3	1.000	1.000	1.000	0.000	1.000
9	1	2	8	13	0.250	0.455	0.114	0.875	0.300
10	1	1	1	3	1.000	1.000	1.000	0.000	1.000
11	1	1	1	4	1.000	1.000	1.000	0.000	1.000
12	1	1	1	2	1.000	1.000	1.000	0.000	1.000
13	1	1	3	6	0.333	0.600	0.200	0.667	0.600
14	1	1	1	3	1.000	1.000	1.000	0.000	1.000
15	1	1	3	3	0.333	0.000	0.000	0.667	0.600
16	2	2	10	19	0.200	0.529	0.106	0.800	0.273
17	2	2	2	13	1.000	1.000	1.000	0.000	1.000
18	1	3	5	11	0.600	0.750	0.450	0.800	0.429
19	1	1	7	12	0.143	0.455	0.065	0.857	0.333
20	2	2	3	10	0.667	0.875	0.583	0.333	0.750
21	2	2	3	12	0.667	0.900	0.600	0.333	0.750
22	2	2	3	9	0.667	0.857	0.571	0.333	0.750
23	1	1	1	3	1.000	1.000	1.000	0.000	1.000
24	1	1	1	3	1.000	1.000	1.000	0.000	1.000
25	2	2	7	13	0.286	0.545	0.156	0.714	0.375
26	3	3	9	25	0.333	0.727	0.242	0.667	0.333
27	1	2	2	5	1.000	1.000	1.000	0.500	0.750
28	1	1	2	6	0.500	0.800	0.400	0.500	0.750
29	2	2	7	22	0.286	0.750	0.214	0.714	0.375
30	3	6	9	17	0.667	0.727	0.485	0.667	0.333
31	2	2	9	20	0.222	0.611	0.136	0.778	0.300
32	3	4	9	30	0.444	0.808	0.359	0.667	0.333
33	1	1	1	12	1.000	1.000	1.000	0.000	1.000

34	1	1	2	17	0.500	0.938	0.469	0.500	0.750
35	1	1	3	10	0.333	0.778	0.259	0.667	0.600
36	1	1	2	11	0.500	0.900	0.450	0.500	0.750
37	1	1	2	14	0.500	0.923	0.462	0.500	0.750
38	3	3	5	18	0.600	0.867	0.520	0.400	0.600
39	1	1	1	4	1.000	1.000	1.000	0.000	1.000
40	1	1	1	15	1.000	1.000	1.000	0.000	1.000
41	1	1	2	10	0.500	0.889	0.444	0.500	0.750
42	3	3	5	20	0.600	0.882	0.529	0.400	0.600
43	2	2	10	21	0.200	0.579	0.116	0.800	0.273
44	1	1	3	18	0.333	0.882	0.294	0.667	0.600
45	1	1	7	16	0.143	0.600	0.086	0.857	0.333
46	3	3	4	27	0.750	0.958	0.719	0.250	0.750
47	3	3	11	28	0.273	0.680	0.185	0.727	0.273
48	3	3	17	26	0.176	0.391	0.069	0.824	0.176
49	1	1	1	3	1.000	1.000	1.000	0.000	1.000
50	1	1	1	3	1.000	1.000	1.000	0.000	1.000
51	3	3	5	21	0.600	0.889	0.533	0.400	0.600
52	2	2	2	15	1.000	1.000	1.000	0.000	1.000
53	2	2	3	8	0.667	0.833	0.556	0.333	0.750
54	1	1	1	8	1.000	1.000	1.000	0.000	1.000
55	3	8	15	38	0.533	0.767	0.409	0.800	0.200
56	1	1	2	5	0.500	0.750	0.375	0.500	0.750
57	1	1	2	7	0.500	0.833	0.417	0.500	0.750
58	2	2	13	20	0.154	0.389	0.060	0.846	0.214
59	3	4	9	25	0.444	0.762	0.339	0.667	0.333
60	1	1	1	4	1.000	1.000	1.000	0.000	1.000
61	2	3	11	17	0.273	0.429	0.117	0.818	0.250
62	2	3	4	11	0.750	0.875	0.656	0.500	0.600
63	4	4	4	8	1.000	1.000	1.000	0.000	1.000
64	1	1	3	4	0.333	0.333	0.111	0.667	0.600
65	2	3	3	5	1.000	1.000	1.000	0.333	0.750
66	2	2	3	5	0.667	0.667	0.444	0.333	0.750
67	2	3	8	14	0.375	0.545	0.205	0.750	0.333
68	2	2	3	9	0.667	0.857	0.571	0.333	0.750
69	2	2	3	10	0.667	0.875	0.583	0.333	0.750
70	1	1	2	6	0.500	0.800	0.400	0.500	0.750
71	1	1	1	4	1.000	1.000	1.000	0.000	1.000
72	2	2	11	14	0.182	0.250	0.045	0.818	0.250
73	1	2	4	10	0.500	0.750	0.375	0.750	0.500
74	2	2	4	6	0.500	0.500	0.250	0.500	0.600
75	2	3	3	6	1.000	1.000	1.000	0.333	0.750

76	1	1	9	17	0.111	0.500	0.056	0.889	0.273
77	2	2	8	12	0.250	0.400	0.100	0.750	0.333
78	1	1	1	1	1.000	0/0	0/0	0.000	1.000
79	1	1	1	1	1.000	0/0	0/0	0.000	1.000
80	1	1	3	11	0.333	0.800	0.267	0.667	0.600
81	2	3	7	7	0.429	0.000	0.000	0.714	0.375
82	2	2	2	9	1.000	1.000	1.000	0.000	1.000
83	2	2	5	8	0.400	0.500	0.200	0.600	0.500
84	4	4	7	10	0.571	0.500	0.286	0.429	0.500
85	2	2	7	10	0.286	0.375	0.107	0.714	0.375
86	1	1	6	9	0.167	0.375	0.063	0.833	0.375
87	1	1	1	5	1.000	1.000	1.000	0.000	1.000
88	1	1	2	5	0.500	0.750	0.375	0.500	0.750
89	1	1	2	4	0.500	0.667	0.333	0.500	0.750
90	2	2	2	9	1.000	1.000	1.000	0.000	1.000
91	1	1	1	5	1.000	1.000	1.000	0.000	1.000
92	1	1	1	2	1.000	1.000	1.000	0.000	1.000
93	1	1	3	5	0.333	0.500	0.167	0.667	0.600
94	1	1	3	7	0.333	0.667	0.222	0.667	0.600
95	1	1	2	3	0.500	0.500	0.250	0.500	0.750
96	1	1	3	3	0.333	0.000	0.000	0.667	0.600
97	1	1	3	5	0.333	0.500	0.167	0.667	0.600
98	1	1	2	5	0.500	0.750	0.375	0.500	0.750
99	1	1	2	6	0.500	0.800	0.400	0.500	0.750
100	1	1	1	3	1.000	1.000	1.000	0.000	1.000
101	1	1	2	4	0.500	0.667	0.333	0.500	0.750
102	1	1	2	4	0.500	0.667	0.333	0.500	0.750

Figure 4 - 50% Majority Rule Consensus Tree with 19 characters ordered

Tree length = 373
Consistency index (CI) = 0.4826
Homoplasy index (HI) = 0.5737
CI excluding uninformative characters = 0.4798
HI excluding uninformative characters = 0.5202
Retention index (RI) = 0.7771
Rescaled consistency index (RC) = 0.3750

```
/------------------------------------------------------------ Sinoconodon
|
+------------------------------------------------------------ Morganucodon
|
|    /------------------------------------------------------ Thomasia
|    |
|    |                                    /---------- Meketichoffatia
```

```
|    |                                      |
|    |                                      +---------- Rugosodon
|    |                                   /-47
|    |                                   |  |  /------ Paulchoffatia
|    |                                   |  |  |
|    |                                   |  \--46  /--- Meketibolodon
|    |   /------------------------------------49     \-45
|    |   |                               |       \--- Guimarotodon
|    |   |                               |
|  /-80  |                               |       /--- Henkelodon
|  | |   |                               \--------48
|  | |   |                                       \--- Kuehneodon
|  | |   |
|  | |   |                                       /--- Ctenacodon
|  | |   |                                       |
|  | |   |  /------------------------------------------50--- Glirodon
|  | |   |  |                                    |
|  | |   |  |                                    \--- Bolodon
|  | |   |  |
82 | |   |  |    /------------------------------------------- Plagiaulax
|  | \--79  |    |
|  |     |  |    |                               /--- Sinobaatar
|  |     |  |    |  /---------------------------------51
|  |     |  |    |  |                            \--- Eobaatar
|  |     |  |    |  |
|  |     |  |    |  |  /------------------------------------ Arginbaatar
|  |     |  |    |  |  |
|  |     |  |  /-76  |  |                          /--- Cimexomys
|  |     |  |  |  |  |  |            /--------------52
|  |     |  |  |  |  |  |            |             \--- Meniscoessus
|  |     |  |  |  |  |  |            |
|  |     |  |  |  |  |  |            |  /---------------- Buginbaatar
|  |     |  |  |  |  |  |          /-59 |
|  |     |  |  |  |  |  |          |  | |          /--- Taeniolabis
|  |     \-78 |  |  |  |          |  | |     /-----53
|  |        |  |  \-75  |          |  | |     |     \--- Catopsalis
|  |        |  |     |  |          |  \-58    |
|  |        |  |     |  |          |    | /-56     /--- Lambdopsalis
|  |        |  |     |  |          |    | |  |   /-54
|  |        |  |     |  |          |    | |  |   | \--- Sphenopsalis
|  |        |  |     |  |          |    \-57 \--55
|  |        |  |     |  |          |      |     \------ Prionessus
|  |        |  |     |  |          |      |
|  |        |  |     |  |          |      \------------- Yubaatar
\-81        |  |     |  |      /--67
   |        |  |     |  |      |   |               /--- Pentacosmodon
   |        |  |     |  |      |   |            /-60
   |        |  |     |  |      |   |            |  \--- Catopsbaatar
   |        |  |    \--74      |   |         /--61
   |        |  |       |       |   |         |  \------ Kryptobaatar
   |        |  |       |       |   |      /-63
   |        |  |       |       |   |      |  |     /--- Kamptobaatar
   |       \-77        |       |   |      |  \-----62
   |          |       |       |   |   /-64        \--- Chulsanbaatar
   |          |       |       |   |   |  |
   |          |       |       |   |   |  \------------- Nemegtbaatar
   |          |       |       |  \----66
   |          |       |       |       |          /--- Eucosmodon
   |          |       |    /-71       \-----------65
   |          |       |    |  |                  \--- Stygimys
   |          |       |    |  |
   |          |       |    |  +------------------------- Boffius
   |          |       |    |  |
```

```
 |          |        |     |  |                      /--- Ectypodus
 |          |        |     |  |                   /-68
 |          |        |     |  |                   |  \--- Ptilodus
 |          |        |  /-72  |                /--69
 |          |        |  |  |  |                |   \------ Neoliotomus
 |          |        |  |  |  +---------------70
 |          |        |  |  |  |                \---------- Mesodma
 |          |        |  |  |  |
 |          |        \-73  |  \-------------------------- Microcosmodon
 |          |           |  |
 |          |           |  \----------------------------- Cimolodon
 |          |           |
 |          |           \-------------------------------- Kogaionon
 |          |
 |          \------------------------------------------- Zofiabaatar
 |
 \------------------------------------------------------ Haramiyavia
```

Apomorphy lists:

Branch	Character	Steps	CI	Change
node_82 --> Sinocon.	78	1	1.000	1 ==> 0
	79	1	1.000	1 ==> 0
node_82 --> Morgan.	84	1	0.571	1 ==> 0
node_82 --> node_81	4	1	0.500	0 --> 1
	5	1	0.125	0 --> 1
	12	1	1.000	0 ==> 1
	22	1	0.667	0 ==> 1
	53	1	0.667	0 ==> 1
	62	1	0.750	0 ==> 1
	63	1	1.000	0 ==> 3
	66	1	0.667	0 --> 1
	68	1	0.667	0 ==> 1
	69	1	0.667	0 ==> 2
	77	1	0.400	0 --> 1
	89	1	0.500	1 --> 0
	93	1	0.333	0 --> 1
	95	1	0.500	0 --> 1
	101	1	0.500	0 --> 1
node_81 --> node_80	1	1	1.000	0 --> 1
	2	1	1.000	0 --> 1
	3	1	0.400	0 --> 1
	8	1	1.000	0 --> 1
	10	1	1.000	0 --> 1
	13	1	0.500	0 --> 1
	14	1	1.000	0 --> 1
	24	1	1.000	0 --> 1

	42	1	0.750	0 ==> 1
	51	1	0.600	0 --> 1
	52	1	1.000	0 --> 1
node_80 --> Thomasia	26	3	0.333	0 ==> 3
	32	1	0.571	0 ==> 1
	66	1	0.667	1 --> 0
node_80 --> node_79	11	1	1.000	0 ==> 1
	17	1	1.000	0 ==> 1
	19	1	0.167	0 --> 1
	20	1	0.667	0 ==> 1
	28	1	0.500	0 --> 1
	29	2	0.286	2 ==> 0
	36	1	0.500	0 ==> 1
	38	1	0.600	0 ==> 1
	41	1	0.500	0 ==> 1
	45	1	0.333	0 ==> 1
	47	2	0.300	0 ==> 2
	48	1	0.214	0 ==> 1
	49	1	1.000	0 ==> 1
	50	1	1.000	0 ==> 1
	56	1	0.500	0 ==> 1
	60	1	1.000	0 ==> 1
	61	1	0.273	1 ==> 0
	72	1	0.250	2 ==> 1
node_79 --> node_49	23	1	1.000	0 ==> 1
	46	1	1.000	1 ==> 0
	63	1	1.000	3 --> 1
	64	1	0.500	0 ==> 1
	65	1	1.000	0 ==> 1
	66	1	0.667	1 --> 2
	68	1	0.667	1 ==> 2
	70	1	0.500	0 ==> 1
	71	1	1.000	0 ==> 1
	75	1	1.000	0 ==> 1
	76	1	0.167	0 ==> 1
	94	1	0.500	0 --> 1
node_49 --> node_47	72	1	0.250	1 ==> 0
node_47 --> Meketich.	56	1	0.500	1 ==> 0
node_47 --> node_46	13	1	0.500	1 ==> 0
	75	1	1.000	1 --> 2
node_46 --> node_45	47	1	0.300	2 ==> 3
node_49 --> node_48	4	1	0.500	1 --> 0

	20	1	0.667	1 ==> 2
	63	1	1.000	1 --> 2
node_48 --> Henkelodon	22	1	0.667	1 ==> 2
node_48 --> Kuehneodon	26	1	0.333	0 ==> 1
	51	1	0.600	1 ==> 2
node_79 --> node_78	5	1	0.125	1 --> 0
	7	1	0.167	0 ==> 1
	9	1	0.286	0 ==> 1
	39	1	1.000	0 --> 1
	53	1	0.667	1 ==> 0
	54	1	1.000	0 ==> 1
	57	1	0.500	0 ==> 1
	62	1	0.750	1 ==> 2
	69	1	0.667	2 ==> 1
	73	1	0.500	0 ==> 1
	77	1	0.400	1 --> 0
	82	1	1.000	0 ==> 1
	97	1	0.500	0 --> 1
	99	1	0.500	0 --> 1
node_78 --> node_50	30	1	0.750	0 ==> 1
	85	1	0.400	0 ==> 1
node_50 --> Ctenacodon	55	1	0.533	0 ==> 1
node_50 --> Glirodon	16	1	0.222	0 ==> 1
	57	1	0.500	1 ==> 0
node_50 --> Bolodon	5	1	0.125	0 ==> 1
	38	1	0.600	1 ==> 2
	76	1	0.167	0 ==> 1
node_78 --> node_77	17	1	1.000	1 --> 2
	18	1	0.600	0 --> 1
	19	1	0.167	1 --> 0
	21	1	0.667	0 --> 1
	22	1	0.667	1 --> 2
	28	1	0.500	1 --> 0
	32	1	0.571	0 --> 1
	48	1	0.214	1 ==> 2
	58	1	0.167	0 --> 1
	80	1	0.333	0 --> 1
node_77 --> node_76	33	1	1.000	0 ==> 1
	38	1	0.600	1 ==> 2
	43	1	0.250	0 ==> 1
node_76 --> node_75	37	1	0.500	0 --> 1
	42	1	0.750	1 ==> 2

	44	1	0.333	0 ==> 1
	46	1	1.000	1 ==> 2
	47	1	0.300	2 ==> 1
	52	1	1.000	1 ==> 2
	77	1	0.400	0 --> 1
node_75 --> node_51	9	1	0.286	1 --> 0
	61	1	0.273	0 ==> 1
	70	1	0.500	0 ==> 1
	76	1	0.167	0 ==> 1
	86	1	0.250	0 --> 1
node_51 --> Sinobaatar	30	1	0.750	0 ==> 2
	55	1	0.533	0 ==> 1
	77	1	0.400	1 --> 0
node_51 --> Eobaatar	16	1	0.222	0 ==> 2
	37	1	0.500	1 --> 0
node_75 --> node_74	3	1	0.400	1 ==> 2
	7	1	0.167	1 --> 0
	36	1	0.500	1 ==> 0
	40	1	1.000	0 ==> 1
	43	1	0.250	1 --> 2
	47	1	0.300	1 ==> 0
	84	1	0.571	1 --> 3
node_74 --> Arginbaat.	48	1	0.214	2 ==> 3
	58	1	0.167	1 --> 0
	80	1	0.333	1 --> 0
node_74 --> node_73	26	1	0.333	0 ==> 1
	30	1	0.750	0 --> 2
	34	1	0.500	0 --> 1
	38	1	0.600	2 --> 3
	48	1	0.214	2 --> 1
	51	1	0.600	1 ==> 2
	55	1	0.533	0 ==> 1
	58	1	0.167	1 --> 2
	59	2	0.500	0 ==> 2
	67	1	0.429	0 --> 2
node_73 --> node_72	19	1	0.167	0 --> 1
	29	1	0.286	0 ==> 1
	31	1	0.286	0 ==> 1
	55	1	0.533	1 ==> 2
	61	1	0.273	0 ==> 1
node_72 --> node_71	5	1	0.125	0 --> 1
	43	1	0.250	2 --> 1

	45	1	0.333	1 --> 0
	46	1	1.000	2 ==> 3
	58	1	0.167	2 --> 1
	59	1	0.500	2 ==> 3
node_71 --> node_67	25	1	0.500	0 ==> 1
	32	1	0.571	1 --> 2
	81	1	0.600	0 ==> 2
	90	1	1.000	0 --> 1
node_67 --> node_59	25	1	0.500	1 ==> 2
	48	1	0.214	1 --> 0
	58	1	0.167	1 --> 2
	86	1	0.250	0 ==> 1
	88	1	0.500	0 --> 1
	90	1	1.000	1 --> 2
	98	1	0.500	0 --> 1
	100	1	1.000	0 --> 1
	102	1	0.500	0 --> 1
node_59 --> node_52	67	1	0.429	2 ==> 1
	76	1	0.167	0 ==> 1
	84	1	0.571	3 --> 1
node_52 --> Cimexomys	34	1	0.500	1 ==> 0
	48	2	0.214	0 ==> 2
	59	1	0.500	3 ==> 2
node_52 --> Meniscoes.	29	1	0.286	1 ==> 2
	31	1	0.286	1 ==> 0
	61	1	0.273	1 ==> 2
	72	1	0.250	1 ==> 2
	74	1	0.500	0 ==> 1
	80	1	0.333	1 ==> 0
node_59 --> node_58	3	1	0.400	2 --> 1
	18	1	0.600	1 --> 0
	20	1	0.667	1 --> 0
	21	1	0.667	1 --> 0
	32	1	0.571	2 ==> 3
	35	1	0.333	0 ==> 1
	44	1	0.333	1 ==> 0
	55	1	0.533	2 --> 3
	83	1	0.400	0 --> 1
node_58 --> Buginbaat.	43	1	0.250	1 ==> 0
	58	1	0.167	2 --> 1
	61	1	0.273	1 ==> 2
node_58 --> node_57	5	1	0.125	1 ==> 0

	16	1	0.222	0 ==> 2
	30	1	0.750	2 --> 3
	31	1	0.286	1 ==> 0
	41	1	0.500	1 ==> 0
	47	1	0.300	0 --> 1
	73	1	0.500	1 ==> 0
node_57 --> node_56	3	1	0.400	1 --> 2
	26	2	0.333	1 ==> 3
	27	1	1.000	0 ==> 1
	29	1	0.286	1 ==> 2
	42	1	0.750	2 ==> 3
	51	1	0.600	2 ==> 3
	67	1	0.429	2 --> 0
	81	1	0.600	2 ==> 0
	92	1	1.000	0 ==> 1
	93	1	0.333	1 ==> 0
node_56 --> node_53	63	1	1.000	3 ==> 4
	65	1	1.000	0 ==> 2
	74	1	0.500	0 ==> 2
node_53 --> Taeniolab.	67	1	0.429	0 --> 2
	72	1	0.250	1 ==> 2
node_53 --> Catopsalis	31	1	0.286	0 ==> 1
node_56 --> node_55	6	1	0.500	0 --> 1
	7	1	0.167	0 --> 1
	19	1	0.167	1 --> 0
	47	1	0.300	1 --> 0
	61	1	0.273	1 ==> 0
	62	1	0.750	2 --> 1
node_55 --> node_54	53	1	0.667	0 ==> 2
	68	1	0.667	1 ==> 2
	69	1	0.667	1 ==> 2
	73	1	0.500	0 ==> 1
	74	1	0.500	0 ==> 1
node_57 --> Yubaatar	55	1	0.533	3 --> 2
	72	1	0.250	1 ==> 2
	84	1	0.571	3 ==> 4
node_67 --> node_66	9	1	0.286	1 ==> 0
	16	1	0.222	0 ==> 2
	59	1	0.500	3 --> 2
	85	1	0.400	0 ==> 1
	87	1	1.000	0 --> 1
	91	1	1.000	0 --> 1

	94	1	0.500	0 ==> 1
	99	1	0.500	1 --> 0
node_66 --> node_64	19	1	0.167	1 ==> 0
	21	1	0.667	1 ==> 2
	55	1	0.533	2 --> 1
	67	1	0.429	2 ==> 0
	82	1	1.000	1 ==> 2
node_64 --> node_63	25	1	0.500	1 ==> 0
	58	1	0.167	1 --> 0
	81	1	0.600	2 ==> 0
	84	1	0.571	3 --> 2
node_63 --> node_61	83	1	0.400	0 ==> 2
	88	1	0.500	0 ==> 1
	89	1	0.500	0 ==> 1
	98	1	0.500	0 ==> 1
	101	1	0.500	1 ==> 0
	102	1	0.500	0 ==> 1
node_61 --> node_60	5	1	0.125	1 ==> 0
	7	1	0.167	0 ==> 1
	9	1	0.286	0 ==> 1
	26	1	0.333	1 --> 2
	29	1	0.286	1 --> 2
	43	1	0.250	1 ==> 0
	51	1	0.600	2 --> 3
	55	1	0.533	1 --> 2
	58	1	0.167	0 --> 1
	59	1	0.500	2 --> 3
	61	1	0.273	1 --> 0
	85	1	0.400	1 --> 0
	86	1	0.250	0 --> 1
	95	1	0.500	1 --> 0
node_60 --> Pentacosm.	16	1	0.222	2 ==> 1
	31	1	0.286	1 ==> 0
	35	1	0.333	0 ==> 1
	47	1	0.300	0 ==> 1
	48	1	0.214	1 ==> 0
node_60 --> Catopsb.	42	1	0.750	2 ==> 1
	44	1	0.333	1 ==> 0
	48	1	0.214	1 ==> 2
	58	1	0.167	1 ==> 2
node_61 --> Kryptob.	61	1	0.273	1 ==> 2
node_63 --> node_62	59	1	0.500	2 ==> 1

	85	1	0.400	1 ==> 2
node_62 --> Kamptob.	6	1	0.500	0 ==> 1
	9	1	0.286	0 ==> 1
	16	1	0.222	2 ==> 1
	84	1	0.571	2 --> 3
	96	1	0.333	1 ==> 0
node_62 --> Chulsanb.	7	1	0.167	0 ==> 1
node_64 --> Nemegtb.	58	1	0.167	1 --> 2
	61	1	0.273	1 ==> 2
	96	1	0.333	1 ==> 0
node_66 --> node_65	31	1	0.286	1 --> 2
	32	1	0.571	2 --> 1
	35	1	0.333	0 ==> 1
	43	1	0.250	1 --> 2
	85	1	0.400	1 --> 2
node_65 --> Eucosmodon	7	1	0.167	0 ==> 1
	26	1	0.333	1 ==> 2
	67	1	0.429	2 ==> 1
	72	1	0.250	1 ==> 2
node_65 --> Stygimys	3	1	0.400	2 ==> 1
	47	1	0.300	0 ==> 1
node_71 --> Boffius	16	1	0.222	0 ==> 1
	55	1	0.533	2 ==> 3
	58	1	0.167	1 --> 2
node_71 --> node_70	15	1	0.500	0 --> 1
	43	1	0.250	1 --> 2
	45	1	0.333	0 --> 1
	76	1	0.167	0 ==> 1
	77	1	0.400	1 ==> 2
	81	1	0.600	0 --> 1
	84	1	0.571	3 --> 2
	93	1	0.333	1 --> 0
	97	1	0.500	1 --> 0
node_70 --> node_69	19	1	0.167	1 ==> 0
	31	1	0.286	1 ==> 2
	48	2	0.214	1 --> 3
	55	1	0.533	2 --> 3
	72	1	0.250	1 ==> 2
node_69 --> node_68	5	1	0.125	1 --> 0
	64	1	0.500	0 ==> 1
node_68 --> Ectypodus	83	1	0.400	0 ==> 2
	86	1	0.250	0 ==> 1

node_68 --> Ptilodus	9	1	0.286	1 ==> 0
	29	1	0.286	1 ==> 0
	48	1	0.214	3 --> 2
	96	1	0.333	1 ==> 0
node_69 --> Neoliotom.	15	1	0.500	1 --> 0
	16	1	0.222	0 ==> 2
	58	1	0.167	1 --> 2
	74	1	0.500	0 ==> 1
node_71 --> Microcos.	5	1	0.125	1 --> 0
	16	1	0.222	0 ==> 1
	32	1	0.571	1 ==> 2
	38	1	0.600	3 ==> 2
	43	1	0.250	1 ==> 0
	47	1	0.300	0 ==> 1
	48	1	0.214	1 --> 2
	83	1	0.400	0 ==> 1
node_72 --> Cimolodon	61	1	0.273	1 ==> 2
	72	1	0.250	1 ==> 2
	76	1	0.167	0 ==> 1
node_73 --> Kogaionon	25	1	0.500	0 ==> 1
	30	1	0.750	2 --> 3
	83	1	0.400	0 ==> 1
node_77 --> Zofiabaat.	48	1	0.214	2 ==> 3
node_81 --> Haramiy.	18	1	0.600	0 ==> 1

Character diagnostics:

Character	Range	Min steps	Tree steps	Max steps	CI	RI	RC	HI	G-fit
1	1	1	1	3	1.000	1.000	1.000	0.000	1.000
2	1	1	1	3	1.000	1.000	1.000	0.000	1.000
3	2	2	5	17	0.400	0.800	0.320	0.600	0.500
4	1	1	2	3	0.500	0.500	0.250	0.500	0.750
5	1	1	8	17	0.125	0.563	0.070	0.875	0.300
6	1	1	2	2	0.500	0.000	0.000	0.500	0.750
7	1	1	6	11	0.167	0.500	0.083	0.833	0.375
8	1	1	1	3	1.000	1.000	1.000	0.000	1.000
9	1	2	7	13	0.286	0.545	0.156	0.857	0.333
10	1	1	1	3	1.000	1.000	1.000	0.000	1.000
11	1	1	1	4	1.000	1.000	1.000	0.000	1.000
12	1	1	1	2	1.000	1.000	1.000	0.000	1.000

13	1	1	2	6	0.500	0.800	0.400	0.500	0.750
14	1	1	1	3	1.000	1.000	1.000	0.000	1.000
15	1	1	2	3	0.500	0.500	0.250	0.500	0.750
16	2	2	9	19	0.222	0.588	0.131	0.778	0.300
17	2	2	2	13	1.000	1.000	1.000	0.000	1.000
18	1	3	5	11	0.600	0.750	0.450	0.800	0.429
19	1	1	6	12	0.167	0.545	0.091	0.833	0.375
20	2	2	3	10	0.667	0.875	0.583	0.333	0.750
21	2	2	3	12	0.667	0.900	0.600	0.333	0.750
22	2	2	3	9	0.667	0.857	0.571	0.333	0.750
23	1	1	1	3	1.000	1.000	1.000	0.000	1.000
24	1	1	1	3	1.000	1.000	1.000	0.000	1.000
25	2	2	4	13	0.500	0.818	0.409	0.500	0.600
26	3	3	9	25	0.333	0.727	0.242	0.667	0.333
27	1	2	2	5	1.000	1.000	1.000	0.500	0.750
28	1	1	2	6	0.500	0.800	0.400	0.500	0.750
29	2	2	7	22	0.286	0.750	0.214	0.714	0.375
30	3	6	8	17	0.750	0.818	0.614	0.625	0.375
31	2	2	7	20	0.286	0.722	0.206	0.714	0.375
32	3	4	7	30	0.571	0.885	0.505	0.571	0.429
33	1	1	1	12	1.000	1.000	1.000	0.000	1.000
34	1	1	2	17	0.500	0.938	0.469	0.500	0.750
35	1	1	3	10	0.333	0.778	0.259	0.667	0.600
36	1	1	2	11	0.500	0.900	0.450	0.500	0.750
37	1	1	2	14	0.500	0.923	0.462	0.500	0.750
38	3	3	5	18	0.600	0.867	0.520	0.400	0.600
39	1	1	1	4	1.000	1.000	1.000	0.000	1.000
40	1	1	1	15	1.000	1.000	1.000	0.000	1.000
41	1	1	2	10	0.500	0.889	0.444	0.500	0.750
42	3	3	4	20	0.750	0.941	0.706	0.250	0.750
43	2	2	8	21	0.250	0.684	0.171	0.750	0.333
44	1	1	3	18	0.333	0.882	0.294	0.667	0.600
45	1	1	3	16	0.333	0.867	0.289	0.667	0.600
46	3	3	3	27	1.000	1.000	1.000	0.000	1.000
47	3	3	10	28	0.300	0.720	0.216	0.700	0.300
48	3	3	14	26	0.214	0.522	0.112	0.786	0.214
49	1	1	1	3	1.000	1.000	1.000	0.000	1.000
50	1	1	1	3	1.000	1.000	1.000	0.000	1.000
51	3	3	5	21	0.600	0.889	0.533	0.400	0.600
52	2	2	2	15	1.000	1.000	1.000	0.000	1.000
53	2	2	3	8	0.667	0.833	0.556	0.333	0.750
54	1	1	1	8	1.000	1.000	1.000	0.000	1.000

55	3	8	15	38	0.533	0.767	0.409	0.800	0.200
56	1	1	2	5	0.500	0.750	0.375	0.500	0.750
57	1	1	2	7	0.500	0.833	0.417	0.500	0.750
58	2	2	12	20	0.167	0.444	0.074	0.833	0.231
59	3	4	8	25	0.500	0.810	0.405	0.625	0.375
60	1	1	1	4	1.000	1.000	1.000	0.000	1.000
61	2	3	11	17	0.273	0.429	0.117	0.818	0.250
62	2	3	4	11	0.750	0.875	0.656	0.500	0.600
63	4	4	4	8	1.000	1.000	1.000	0.000	1.000
64	1	1	2	4	0.500	0.667	0.333	0.500	0.750
65	2	3	3	5	1.000	1.000	1.000	0.333	0.750
66	2	2	3	5	0.667	0.667	0.444	0.333	0.750
67	2	3	7	14	0.429	0.636	0.273	0.714	0.375
68	2	2	3	9	0.667	0.857	0.571	0.333	0.750
69	2	2	3	10	0.667	0.875	0.583	0.333	0.750
70	1	1	2	6	0.500	0.800	0.400	0.500	0.750
71	1	1	1	4	1.000	1.000	1.000	0.000	1.000
72	2	2	8	14	0.250	0.500	0.125	0.750	0.333
73	1	2	4	10	0.500	0.750	0.375	0.750	0.500
74	2	2	4	6	0.500	0.500	0.250	0.500	0.600
75	2	3	3	6	1.000	1.000	1.000	0.333	0.750
76	1	1	6	17	0.167	0.688	0.115	0.833	0.375
77	2	2	5	12	0.400	0.700	0.280	0.600	0.500
78	1	1	1	1	1.000	0/0	0/0	0.000	1.000
79	1	1	1	1	1.000	0/0	0/0	0.000	1.000
80	1	1	3	11	0.333	0.800	0.267	0.667	0.600
81	2	3	5	7	0.600	0.500	0.300	0.600	0.500
82	2	2	2	9	1.000	1.000	1.000	0.000	1.000
83	2	2	5	8	0.400	0.500	0.200	0.600	0.500
84	4	4	7	10	0.571	0.500	0.286	0.429	0.500
85	2	2	5	10	0.400	0.625	0.250	0.600	0.500
86	1	1	4	9	0.250	0.625	0.156	0.750	0.500
87	1	1	1	5	1.000	1.000	1.000	0.000	1.000
88	1	1	2	5	0.500	0.750	0.375	0.500	0.750
89	1	1	2	4	0.500	0.667	0.333	0.500	0.750
90	2	2	2	9	1.000	1.000	1.000	0.000	1.000
91	1	1	1	5	1.000	1.000	1.000	0.000	1.000
92	1	1	1	2	1.000	1.000	1.000	0.000	1.000
93	1	1	3	5	0.333	0.500	0.167	0.667	0.600
94	1	1	2	7	0.500	0.833	0.417	0.500	0.750
95	1	1	2	3	0.500	0.500	0.250	0.500	0.750
96	1	1	3	3	0.333	0.000	0.000	0.667	0.600

97	1	1	2	5	0.500	0.750	0.375	0.500	0.750
98	1	1	2	5	0.500	0.750	0.375	0.500	0.750
99	1	1	2	6	0.500	0.800	0.400	0.500	0.750
100	1	1	1	3	1.000	1.000	1.000	0.000	1.000
101	1	1	2	4	0.500	0.667	0.333	0.500	0.750
102	1	1	2	4	0.500	0.667	0.333	0.500	0.750

V. References

Cifelli, R.L., Gordon, C.L., Lipka, T.R., and Scott, C.S. 2013. New multituberculate mammal from the Early Cretaceous of eastern North America. *Canadian Journal of Earth Sciences* 50: 315–323.

Kielan-Jaworowska, Z. and Hurum, J.H. 2001. Phylogeny and systematics of multituberculate mammals. *Palaeontology* 44 (3): 389–429.

Luo, Z.-X., Kielan-Jaworowska, Z., and Cifelli, R.L. 2002. In quest for a phylogeny of Mesozoic mammals. *Acta Palaeontologica Polonica* 47 (1): 1–78.

Mao, F-Y, Wang Y-Q, Meng J. In press. New material of *Sphenopsalis* (Multituberculate, Mammalia) from Inner Mongolia of China and its implications for phylogeny and biology of taeniolabidoid multituberculates. Acta Palaeontologica Polonica. Doi: http://dx.doi.org/10.4202/app.00117.2014

Rougier, G.W., Novacek, M.J., and Dashzeveg, D. 1997. A new multituberculate from the late Cretaceous locality Ukhaa Tolgod, Mongolia: considerations on multituberculate interrelationships. *American Museum Novitates* 3191: 1–26.

Weil, A. 1998. A new species of Microcosmodon (Mammalia: Multituberculata) from the Paleocene Tullock Formation of Montana, and an argument for the Microcosmodontinae. PaleoBios 18 (2&3):1-15.

Yuan, C.-X., Ji, Q., Meng, Q.-J., Tabrum, A.R., and Luo, Z.-X. 2013. Earliest Evolution of Multituberculate Mammals Revealed by a New Jurassic Fossil. *Science* 341 (6147): 779–783.
